# European Heart Rhythm Association (EHRA)/Heart Rhythm Society (HRS)/Asia Pacific Heart Rhythm Society (APHRS)/Latin American Heart Rhythm Society (LAHRS) expert consensus on risk assessment in cardiac arrhythmias: use the right tool for the right outcome, in the right population

**DOI:** 10.1093/europace/euaa065

**Published:** 2020-06-15

**Authors:** Jens Cosedis Nielsen, Yenn-Jiang Lin, Marcio Jansen de Oliveira Figueiredo, Alireza Sepehri Shamloo, Alberto Alfie, Serge Boveda, Nikolaos Dagres, Dario Di Toro, Lee L Eckhardt, Kenneth Ellenbogen, Carina Hardy, Takanori Ikeda, Aparna Jaswal, Elizabeth Kaufman, Andrew Krahn, Kengo Kusano, Valentina Kutyifa, Han S Lim, Gregory Y H Lip, Santiago Nava-Townsend, Hui-Nam Pak, Gerardo Rodríguez Diez, William Sauer, Anil Saxena, Jesper Hastrup Svendsen, Diego Vanegas, Marmar Vaseghi, Arthur Wilde, T Jared Bunch, ESC Scientific Document Group, Alfred E Buxton, Gonzalo Calvimontes, Tze-Fan Chao, Lars Eckardt, Heidi Estner, Anne M Gillis, Rodrigo Isa, Josef Kautzner, Philippe Maury, Joshua D Moss, Gi-Byung Nam, Brian Olshansky, Luis Fernando Pava Molano, Mauricio Pimentel, Mukund Prabhu, Wendy S Tzou, Philipp Sommer, Janice Swampillai, Alejandro Vidal, Thomas Deneke, Gerhard Hindricks, Christophe Leclercq

**Affiliations:** e1 Department of Cardiology, Aarhus University Hospital, Skejby, Denmark; e2 Division of Cardiology, Department of Medicine, Taipei Veterans General Hospital, Taipei, Taiwan; e3 Department of Internal Medicine, Electrophysiology Service, University of Campinas Hospital, Campinas, Brazil; e4 Department of Electrophysiology, Leipzig Heart Center at University of Leipzig, Leipzig, Germany; e5 Division of Electrophysiology, Instituto Cardiovascular Adventista, Clinica Bazterrica, Buenos Aires, Argentina; e6 Department of Cardiology, Clinique Pasteur, Toulouse, France; e7 Department of Cardiology, Division of Electrophysiology, Argerich Hospital and CEMIC, Buenos Aires, Argentina; e8 Department of Medicine, University of Wisconsin-Madison, Madison, WI, USA; e9 Division of Cardiology, Virginia Commonwealth University School of Medicine, Richmond, USA; e10 Arrhythmia Unit, Heart Institute, University of São, Paulo Medical School, Instituto do Coração -InCor- Faculdade de Medicina de São Paulo-São Paulo, Brazil; e11 Department of Cardiovascular Medicine, Faculty of Medicine, Toho University, Japan; e12 Department of Cardiac Electrophysiology, Fortis Escorts Heart Institute, Okhla Road, New Delhi, India; e13 The Heart and Vascular Research Center, Metrohealth Campus of Case Western Reserve University, Cleveland, OH, USA; e14 Division of Cardiology, Department of Medicine, University of British Columbia, Vancouver, Canada; e15 Division of Arrhythmia and Electrophysiology, Department of Cardiovascular Medicine, National Cerebral and Cardiovascular Center, Osaka, Japan; e16 University of Rochester, Medical Center, Rochester, USA; e17 Semmelweis University, Heart and Vascular Center, Budapest, Hungary; e18 Department of Cardiology, Austin Health, Melbourne, VIC, Australia; e19 University of Melbourne, Melbourne, VIC, Australia; e20 Liverpool Centre for Cardiovascular Science, University of Liverpool and Liverpool Heart & Chest Hospital, Liverpool, UK; e21 Aalborg Thrombosis Research Unit, Department of Clinical Medicine, Aalborg University, Aalborg, Denmark; e22 Department of Electrocardiology, National Institute of Cardiology “Ignacio Chavez,” Mexico City, Mexico; e23 Division of Cardiology, Department of Internal Medicine, Yonsei University Health System, Seoul, Republic of Korea; e24 Department of Electrophysiology and Hemodynamic, Arrhytmias Unity, CMN 20 de Noviembre, ISSSTE, Mexico City, Mexico; e25 Cardiovascular Division, Brigham and Women s Hospital and Harvard Medical School, Boston, USA; e27 Department of Cardiology, Rigshospitalet, University of Copenhagen, Copenhagen, Denmark; e28 Department of Clinical Medicine, Faculty of Health and Medical Sciences, University of Copenhagen, Copenhagen, Denmark; e29 Hospital Militar Central, Fundarritmia, Bogotá, Colombia; e30 Los Angeles UCLA Cardiac Arrhythmia Center, UCLA Health System, David Geffen School of Medicine, at UCLA, USA; e31 Amsterdam UMC, University of Amsterdam, Heart Center; Department of Clinical and Experimental Cardiology, Meibergdreef 9, 1105 AZ, Amsterdam, The Netherlands; e32 Department of Medicine, Intermountain Heart Institute, Intermountain Medical Center, Salt Lake City, USA; e33 Department of Medicine, The Richard A. and Susan F. Smith Center for Outcomes Research in Cardiology, Beth Israel Deaconess Medical Center, Boston, MA, USA; e34 Asociacion Guatemalteca de Cardiologia, Guatemala, Guatemala; e35 Department for Cardiology, Electrophysiology, University Hospital Münster, Münster, Germany; e36 Department of Medicine, I, University Hospital Munich, Ludwig-Maximilians University, Munich, Germany; e37 University of Calgary - Libin Cardiovascular Institute of Alberta, Calgary, AB, Canada; e38 Clínica RedSalud Vitacura and Hospital el Carmen de Maipú, Santiago, Chile; e39 Institute for Clinical and Experimental Medicine, Prague, Czech Republic; e40 Rangueil University Hospital, Toulouse, France; e41 Department of Cardiac Electrophysiology, University of California San Francisco, San Francisco, USA; e42 Division of Cardiology, Asan Medical Center, University of Ulsan, College of Medicine, Seoul, Republic of Korea; e43 University of Iowa Carver College of Medicine, Iowa City, USA; e44 Fundación Valle del Lili, Cali, Colombia; e45 Cardiology Division, Hospital de Clínicas de Porto Alegre, Porto Alegre, RS, Brazil; e46 Department of Cardiology, Sree Chitra Tirunal Institute for Medical Sciences and Technology, Trivandrum, India; e47 Department of Cardiology/Cardiac Electrophysiology, University of Colorado Anschutz Medical Campus, Aurora, USA; e48 Clinic for Electrophysiology, Herz- und Diabeteszentrum, Clinic for Electrophysiology, Ruhr-Universität Bochum, Bad Oeynhausen, Germany; e49 Waikato Hospital, Hamilton, New Zealand; e50 Division of Cardiology, McGill University Health Center, Montreal, Canada; e51 Clinic for Cardiology II (Interventional Electrophysiology), Heart Center Bad Neustadt, Bad Neustadt a.d. Saale, Germany; e52 Univ Rennes, CHU Rennes, INSERM, Rennes, France

## Table of contents

Introduction  1148a
 Evidence review  1148a
 Relationships with industry and other conflicts  1148a
General tools for risk assessment, strengths, limitations, and pretest probability  1148b
 Value of clinical history and characteristics including clinical risk scores such as CHA_2_DS_2_-VASc  1148b
 Electrocardiographic methods including monitoring  1148c
  Electrocardiographic methods  1148c
  P wave and PR interval  1148c
  QRS, QT interval, and T-wave  1148d
  Ambulatory electrocardiogram monitoring  1148e
 Imaging  1148e
  Risk assessment of ventricular tachyarrhythmia using imaging modalities  1148e
  Imaging modalities for atrial arrhythmias  1148e
 Invasive electrophysiological study  1148f
 Implantable loop recorders  1148g
  Implantable loop recorder to diagnose unexplained syncope/atrial fibrillation with cryptogenic stroke  1148g
  Implantable loop recorder to diagnose atrial and ventricular arrhythmia events  1148g
 Wearables/direct to consumer  1148g
 Biomarkers, tissue, genetics  1148h
  Biomarkers  1148h
  Tissue diagnostics  1148i
  Genetics  1148i
 Artificial intelligence  1148i
How to assess risk for atrial fibrillation in specific populations  1148i
 Patients of advanced age  1148i
 Patients with heart failure  1148k
  Clinical risk factors  1148l
  Electrocardiography  1148l
  Biomarkers  1148l
  Imaging  1148l
  Genetics  1148l
 Patients with obesity, hypertension, diabetes, sleep apnoea or structural heart disease  1148m
 Patients who have undergone cardiac surgery  1148n
 Patients with cryptogenic stroke  1148n
 How to assess high risk of atrial fibrillation in professional athletes  1148o
  Atrial fibrillation risk in athletes—general  1148o
  Atrial fibrillation risk in athletes—exercise paradox  1148o
  Atrial fibrillation risk in athletes—structural cardiac changes  1148p
 Patients with inherited rhythm disease (long QT syndrome/short QT syndrome/catecholaminergic polymorphic ventricular tachyarrhythmia/Brugada syndrome)  1148p
How to assess risk for adverse outcomes in patients with atrial fibrillation  1148q
 Risk assessment for stroke/transient ischaemic attack/cognitive decline  1148q
 Risk assessment for stroke/transient ischaemic attack status post-left atrial appendage occlusion/ligation  1148q
 Risk for heart failure incidence and progression  1148r
 Risk for death in atrial fibrillation patients  1148s
 Risk of adverse outcomes in patients treated with catheter ablation  1148t
  Post-ablation atrial fibrillation recurrence  1148t
  Other adverse outcomes  1148t
  Catheter ablation in Wolff–Parkinson–White patients  1148u
 Risk of adverse outcomes in patients treated with surgical Maze  1148u
  Atrial fibrillation surgery  1148u
  Surgical Maze in patients with concomitant heart surgery  1148u
  Stand-alone surgical Maze  1148u
  Left atrial appendage exclusion or removal during surgical Maze  1148u
How to assess risk for ventricular tachyarrhythmia in specific populations  1148u
 Patients with ischaemic heart disease  1148u
  Secondary prevention of ventricular tachyarrhythmia/ ventricular fibrillation in patients with ICM  1148v
  Primary prevention of ventricular tachyarrhythmia/ventricular fibrillation in patients with ICM and a left ventricular ejection fraction ≤35%  1148v
  Primary prevention of ventricular tachyarrhythmia/ ventricular fibrillation in patients with ICM and left ventricular ejection fraction > 35%  1148v
 Patients with non-ischaemic heart failure  1148w
 Patients with inflammatory cardiomyopathies  1148x
 Patients with congenital heart disease  1148x
 Patients with inherited arrhythmia diseases (Inherited channelopathies and inherited structural diseases including arrhythmogenic right ventricular cardiomyopathy)  1148y
 Risk stratification in patients with arrhythmogenic cardiomyopathy, specified for arrhythmogenic right ventricular cardiomyopathy  1148z
 Patients with Chagas disease  1148aa
How to assess risk for adverse outcomes in patients with ventricular tachyarrhythmia  1148aa
 Risk for appropriate and inappropriate implantable cardioverter-defibrillator therapies  1148aa
  Appropriate shock predictors  1148ab
  Inappropriate shock predictors  1148ab
 Risk for heart failure incidence and progression  1148ab
 Risk for death in ventricular tachyarrhythmia patients  1148ac
 Risk of adverse outcomes in patients treated with catheter ablation  1148ad
How to assess risk for adverse outcome in patients with other specific cardiac conditions  1148ae
 Patients with ventricular premature contractions  1148ae
  Premature ventricular complex frequency  1148ae
  Premature ventricular complex morphology  1148ae
  Premature ventricular complex coupling interval  1148ae
 Patients with supraventricular tachyarrhythmia such as Wolff–Parkinson–White syndrome and focal atrial tachycardia  1148ae
Summary  1148af
References  1148ah

## Introduction

Patients with cardiac diseases or conditions with high risk of developing cardiac diseases undergo risk assessment by cardiologists, primary care physicians, and scientists based on referral for more advanced risk assessment strategies, institution of preventive treatments, counselling of patients and their relatives, and selection of patients for scientific trials. The various methods used for risk assessment differ with respect to availability, complexity, and usefulness in different patient populations. Parameters associated with increased risk of e.g. death may also be associated with higher risk of other adverse outcomes. However, risk assessment strategies including specific methods for risk assessment and risk scores should be used only for the purposes for which they are validated. 

This expert consensus statement of the European Heart Rhythm Association (EHRA), Heart Rhythm Society (HRS), Asia Pacific Heart Rhythm Society (APHRS), and the Latin American Heart Rhythm Society (LAHRS) summarizes the consensus of the international writing group based on a thorough review of the medical literature regarding risk assessment in cardiac arrhythmias. To create a tool for clinicians to perform rational and evidence-based risk stratification, this task force was set down by EHRA, HRS, LAHRS, and APHRS, including representatives from each of the four societies.

With this document, we intend to describe and review status of performing risk assessment in different patient populations with cardiac diseases or conditions with high risk of developing such. Our objectives are to raise awareness of using the right risk assessment tool for a given outcome in a given population, and to provide physicians with practical proposals that may lead to improvement of patient care in this regard. For quick reference, sub-chapters start with a short section on consensus statements. The document concludes with a summary of consensus statements.

## 

### Evidence review

Members of the Task Force were asked to perform a detailed literature review using PubMed and EMBASE, weigh the strength of evidence for or against a particular treatment or procedure, and include estimates of expected health outcomes for which data exist. Patient-specific modifiers, comorbidities, and issues of patient preference that might influence the choice of particular tests are considered, as are frequency of follow-up and cost-effectiveness. In controversial areas, or with regard to issues without evidence other than usual clinical practice, consensus was achieved by agreement of the expert panel after thorough deliberations. This document was prepared by the Task Force and peer-reviewed by official external reviewers representing EHRA, HRS, APHRS, and LAHRS.

Consensus statements are evidence-based and derived primarily from published data or determined through consensus opinion if no data available. Current systems of ranking level of evidence are becoming complicated in a way that might compromise their practical utility.^1^ In contrast to guidelines, we opted for an easier user-friendly system of ranking using ‘coloured hearts’ that should allow physicians to easily assess the current status of the evidence and consequent guidance (*Table 1*). This EHRA grading of consensus statements does not have separate definitions of the level of evidence. The categorization used for consensus statements must not be considered directly similar to the one used for official society guideline recommendations which apply a classification (Class I–III) and level of evidence (A, B, and C) to recommendations used in official guidelines.

**Table 1:** Scientific rationale of consensus statements

Definitions related to a treatment or procedure	Consensus statement instruction	Symbol
Scientific evidence that a treatment or procedure is beneficial and effective. Requires at least one randomized trial, or is supported by strong observational evidence and authors’ consensus (as indicated by an asterisk).	‘Should do this’	
General agreement and/or scientific evidence favour the usefulness/efficacy of a treatment or procedure. May be supported by randomized trials based on a small number of patients or not widely applicable.	‘May do this’	
Scientific evidence or general agreement not to use or recommend a treatment or procedure.	‘Do not do this’	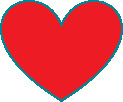

The categorization for our consensus document should not be considered directly similar to the one used for official society guideline recommendations which apply a classification (I–III) and level of evidence (A, B, and C) to recommendations.

Thus, a green heart indicates a ‘should do this’ consensus statement or indicated risk assessment strategy based on at least one randomized trial or supported by strong observational evidence that it is beneficial and effective. A yellow heart indicates general agreement and/or scientific evidence favouring a ‘may do this’ statement or the usefulness/efficacy of a risk assessment strategy or procedure. A ‘yellow heart’ symbol may be supported by randomized trials based on a small number of patients or not widely applicable. Risk assessment strategies for which there is scientific evidence of no benefit or potential harm and should not be used (‘do not do this’) are indicated by a red heart.

Finally, this consensus document includes evidence and expert opinions from several countries. The risk assessment approaches discussed may therefore include tests not approved by governmental regulatory agencies in all countries.

### Relationships with industry and other conflicts

All members of the writing group, as well as reviewers, have disclosed any potential conflicts of interest. Details are available in Supplementary material online.

All consensus statements were voted upon by the writing committee independently and reached the predefined level of ≥75% consensus for inclusion in consensus statement tables. Each partner society officially reviewed the document, and all reviewer comments were addressed. Each partner society approved the final document and consensus statements.

## General tools for risk assessment, strengths, limitations, and pretest probability

### Value of clinical history and characteristics including clinical risk scores such as CHA_2_DS_2_-VASc

Clinical assessment of the patient with cardiac arrhythmias starts with a good clinical history and basic investigations for an underlying aetiological factor for the arrhythmia or its associated complication(s). In addition, an assessment of the risks and benefits of any therapeutic intervention should be made, and appropriate management initiated.

Following on from clinical history and assessment, there is a proposal toward a more integrated and holistic approach to arrhythmia management, as evident in guidelines. Such an integrated approach requires multidisciplinary teams of healthcare professionals, patient involvement, access to treatment options, and decision-support tools to optimize the patient journey. Many proposals have been made towards the operationalization of such an integrated approach to risk assessment and practical management in cardiac arrhythmias, which has been of varying complexity. As an example, the management of atrial fibrillation (AF) has been simplified into the ABC pathway (‘A’ Avoid stroke with Anticoagulation; ‘B’ Better symptom management, with patient-centred and symptom-directed decisions on rate or rhythm control; ‘C’ Cardiovascular and comorbidity risk management), which has been shown to be associated with improved clinical outcomes and reduced healthcare costs.^2–6^

This makes a strong argument for using the right approaches and clinical tools for patient assessment, but using them appropriately for the reasons they were first proposed (e.g. stroke risk scores to assess stroke risk, and not other outcomes).

Taking AF as an *illustrative example* with regard to using the right score for the right reason there are many risk factors for stroke (but the more common and validated ones have been used to formulate risk stratification tools).^7^ The most common in use is the CHA_2_DS_2_-VASc score^8^ but it is not meant to include every possible stroke risk factor, and was designed to be simple, reductionist and practical to help decision-making for stroke risk. As with all clinical scores based on clinical factors, the CHA_2_DS_2_-VASc score only performs modestly for predicting high-risk patients who sustain events. The use of more clinical factors and biomarkers improves prediction (at least statistically) but the practical added value is marginal, and less impressive in real-world cohorts.^9^^,^^10^ Use of simplified scores to artificially categorize patients into low-, moderate- and high-risk strata can be problematic, as in the real-world patients do not necessarily fall into three neat categories of risk. Also, not all risk factors carry equal weight, hence, the move to focus the initial decision-making on identifying low-risk patients who do not need antithrombotic therapy first, following which stroke prevention can be offered to AF patients with ≥1 stroke risk factors.^9^ Stroke risk is also highly dynamic, and although logistically challenging, a clinical reassessment may be needed every 4–6 months to optimize risk re-assessment.^11–13^ As the CHA_2_DS_2_-VASc is a cluster of common cardiovascular risk factors, it is predictive of death, cardiovascular hospitalizations, and other adverse outcomes that the CHA_2_DS_2_-VASc score was not designed for. Also, given that many components of the CHA_2_DS_2_-VASc score are associated with incident AF, the CHA_2_DS_2_-VASc score is used to predict new onset AF, again something it was not designed for. Another misuse of the CHA_2_DS_2_-VASc score is the prediction of bleeding risk. Nevertheless, formal comparisons show that the CHA_2_DS_2_-VASc (and older CHA_2_DS_2_) score are inferior to a formal bleeding risk score such as the HAS-BLED score, for the prediction of major bleeding in AF patients.^14^

Indeed, bleeding risk is also highly dynamic, and the appropriate use of bleeding risk scores such as HAS-BLED is to address modifiable bleeding risk factors (e.g. uncontrolled hypertension, labile INR, concomitant aspirin, or NSAID use) then to schedule the ‘high risk’ patients for early and more frequent follow-up visits (e.g. 4 weeks rather than 4 months).^15^ Only focusing on modifiable bleeding risk factors is an inferior strategy for bleeding risk assessment, compared to the HAS-BLED score.^8^

We should use the scores only for the purposes they were designed for. Attention to appropriate methodology, statistics, etc.—as well as other clinical states merits consideration e.g. sudden death prediction (or failed ablation, device infection, etc.), Charlson Comorbidity Index, frailty etc.—but using the right score designed for that purpose.

If appropriately used, some of these (simplified) tools help with clinical management. Indeed, the value of a medical test is measured by its accuracy as well as how it impacts medical decisions and ultimately patient health. As medical tests are considered and new ones emerge, they should be considered and evaluated in a framework of accuracy and patient impact.^16^ A test must not only be accurate, but also feasible. Tests that are difficult to reproduce, subject to technical failures, or difficult to interpret are likely to impact patient care as a consequence of a primary failure to produce a definitive and actionable result.

### Electrocardiographic methods including monitoring

**Table:** 

Electrocardiographic methods including monitoring	Class	References
Twelve-lead electrocardiogram (ECG) should be obtained in all patients undergoing evaluation for known or suspected heart disease.		^17^
The 12-lead ECG provides diagnostic and prognostic information in patients with inherited high-risk syndromes including long QT syndrome (LQTS), short QT syndrome, Brugada Syndrome, and arrhythmogenic cardiomyopathy (ACM) and should be obtained.		^17^
Exercise ECG provides diagnostic and prognostic information for patients with LQTS ACM, hypertrophic cardiomyopathy (HCM), catecholaminergic polymorphic ventricular tachycardia, and documented or suspected arrhythmias related to exertion, and should be obtained.		^17^
Ambulatory ECG evidence of non-sustained ventricular tachycardia provides prognostic information in ischaemic cardiomyopathy, ACM, and HCM and should be obtained.		^17^
The signal-averaged ECG and QRS fragmentation may aid in the diagnosis of ACM.		^18^
The signal-averaged ECG and QRS fragmentation may be useful in risk stratification of Brugada syndrome.		^18^
Heart rate variability, heart rate turbulence, signal-averaged ECG, and T wave alternans analysis, when used in combination with additional clinical, electrocardiographic, and structural measures, may be useful for identifying high- and low-risk groups among patients with acquired structural heart disease.		^19^

#### Electrocardiographic methods

The ECG is the gold standard for risk assessment in patients with or at risk of developing cardiac arrhythmias. The 12-lead ECG is inexpensive and widely available. Risk stratification with the ECG is limited in general by its low positive predictive value (PPV) determined to a large extent by the low prevalence of cardiovascular events in the general population. However, the prognostic significance of the ECG is enhanced in patients with heart disease.

#### P wave and PR interval

The prognostic value of P wave characteristics has been examined in subjects enrolled in clinical trials of AF for prediction of the development of AF, where maximum P wave duration was a significant independent risk marker for the development of AF over 10 years.^20^ This observation was confirmed by epidemiologic/population studies (including ARIC and the Copenhagen ECG studies) that showed increased risk of AF in patients with prolonged P wave duration and PR interval prolongation,^21–23^ and summarized in a review by Nikolaidou *et al.*^24^ Moreover, a prolonged P wave duration was determined as a sensitive predictor of post-operative AF in patients undergoing coronary artery bypass grafting (CABG).^25^ The definition of an abnormal P wave varies greatly depending on how it is measured, and definitions vary depending on whether P wave area, duration, terminal forces in lead V1 or signal-averaged P wave are analysed. Abnormal P wave morphology was associated with incident stroke in the Multi-Ethnic Study of Atherosclerosis.^26^ The prognostic significance of PR interval prolongation, which is variably defined as PR intervals greater than 196–220 ms, is controversial and depends on the patient population studied. Most studies show that PR interval prolongation is not associated with increased mortality in healthy middle-aged individuals during medium term follow-up. On the other hand, a number of reports show worse survival in patients with suspected heart failure (acute and chronic) or heart disease [coronary artery disease (CAD)]. Additionally, PR prolongation and P wave prolongation predict increased risk of AF and the greater degrees of PR prolongation and P wave duration predicted higher risks of AF.^27^^,^^28^ An increased PR interval is also associated with poor cardiovascular outcomes in patients with AF.^29^ Several studies have shown that PR prolongation in patients undergoing cardiac pacing or receiving cardiac resynchronization therapy (CRT) is an independent predictor of worse prognosis and lower probability of reverse remodelling as well as an increased risk of AF, death, and hospitalization.^30^^,^^31^ There are no data indicating whether the degree of PR prolongation portends a worse outcome compared to patients who have lesser degrees of PR prolongation, nor is there information on its prognostic value in acute inferior wall myocardial infarction (MI).

#### QRS, QT interval, and T-wave

Over the years, a number of ECG techniques have been developed to assess risk of ventricular tachyarrhythmias (VTs). These have the advantage of being non-invasive and, often, inexpensive. For almost all of these techniques, there are conflicting data, and not one technique has proven beneficial in patients with structural heart disease. Moreover, studies have varied in their reporting of sudden arrhythmic death vs. total mortality. Among the risk predictors shown to have value are QRS widening and fragmentation, QT prolongation, T-wave abnormalities, and ventricular ectopy. Although the prognostic value of each ECG parameter in isolation is limited, in combination with additional ECG, imaging, and genetic testing, these parameters can contribute to effective risk stratification.

##### QRS

QRS prolongation has been associated with all-cause mortality in heart failure patients, implantable cardioverter-defibrillator (ICD) shocks, and inducibility of sustained VT. QRS prolongation in patients on Class IC antiarrhythmic drugs is a predictor of pro-arrhythmia, and should be monitored, particularly during exercise. QRS prolongation predicts risk in patients with myotonic dystrophy and in Brugada Syndrome. Additional prognostic information from the QRS is obtained from the signal-averaged ECG, which amplifies the QRS, averages multiple complexes to reduce noise, and filters out the T-wave in order to detect late potentials, and provides evidence of slow conduction substrate that associates with risk of re-entry tachyarrhythmias.^17^ The signal-averaged ECG has been used to detect risk of ventricular arrhythmias in post-infarction patients, ACM, and Brugada Syndrome. Although its specificity is limited, its negative predictive value is high, particularly in survivors of inferior wall myocardial infarction. The signal-averaged ECG is not useful in patients with underlying bundle branch block. QRS fragmentation, which includes abnormally notched narrow and wide QRS complexes, is associated with the presence of myocardial scar and is also associated with mortality in patients with cardiomyopathy and with Brugada Syndrome.^32^ The presence of an unprovoked type 1 Brugada Syndrome pattern is associated with increased risk as is discussed later in the document.

##### QT interval

Measurement of the QT interval can be complicated by QRS prolongation and by the need to correct for heart rate, as has been described elsewhere.^33^ Despite these limitations, prolongation of the heart rate-corrected QT interval (QTc) has been associated with mortality in several population studies.^34^^,^^35^ In congenital long QT syndrome (LQTS), the length of the QT interval is a major predictor of risk of cardiac events, including sudden cardiac death (SCD). When initiating QT-prolonging drugs such as sotalol or dofetilide, a QT interval of 500 ms or higher should prompt reduction or discontinuation of the offending drug(s).

##### QT dispersion

This measure of ventricular repolarization heterogeneity is typically defined from the 12-lead ECG as the QT_max_ − QT_min_. It has been used to predict a wide variety of events, including ventricular pro-arrhythmia, VTs, although the sensitivity, specificity, and accuracy are poorly defined and highly dependent on the patient population studied.^36^

##### T wave

T wave inversions are common and may be non-specific or may signal important abnormalities such as ischaemia or hypertrophy. Widespread deep T wave inversions in combination with QT prolongation, such as may occur in acute stress cardiomyopathy, can be associated with torsades de pointes. Abnormal T wave notching can be a clue to abnormal repolarization and is often seen in patients with QT prolongation. Computerized T-wave analytic techniques such as principal component analysis, T-wave residuum, flatness, asymmetry, and notching have been developed in an effort to detect and quantify abnormal repolarization and may have particular value in identifying patients with LQTS.^37^^,^^38^ Moreover, it has been shown that adding T-wave morphology characterizations to age, gender, and QTc in a support vector machine model can improve LQTS diagnosis.^39^ However, these additional analytic techniques are not used in routine clinical practice.

The Tpeak-end interval, measured from the peak to the end of the T-wave, thought to reflect heterogeneity of repolarization in the heart, has been associated with arrhythmic risk in various populations.^40^ However, considerable controversy remains as to how it should be measured and applied.^41^

T-wave alternans is a beat-to-beat alternation of T wave morphology. When seen with the naked eye, it usually accompanies marked QT prolongation and is a harbinger of imminent torsades de pointes. Analysis of more subtle T-wave alternans has been used for assessing abnormal and heterogeneous repolarization to predict mortality and arrhythmic risk. Abnormal microvolt T-wave alternans assessed using the spectral method during graded exercise has a high negative predictive value and has been used to identify a subgroup of patients with reduced ejection fraction who are not likely to benefit from defibrillator implantation.^18^ Microvolt T-wave alternans analysis cannot be performed when the rhythm is AF, and patients with ventricular pacing have not been studied extensively.

##### Early repolarization

Early repolarization pattern, highly prevalent in the overall population, defined as an elevation of the J point of at least 0.1 mV, may occur in the anteroseptal or inferolateral leads. In 2008, Haissaguerre reported an association of inferolateral early repolarization with increased risk of idiopathic ventricular fibrillation (VF) in a case–control study^42^ and subsequently confirmed in other case–control studies. Exercise testing or isoproterenol testing improved the pattern of repolarization, and the pattern was accentuated with exposure to beta-adrenergic blockers. In a meta-analysis of population-based studies, inferolateral early repolarization was associated with increased risk of arrhythmic death, but the risk was still quite low in general (70/100 000 patient-years).^43^ It appears that individuals at highest risk have early repolarization in multiple (especially inferior) leads, with high voltage (at least 0.2 mV), and with notching or horizontal/down-sloping ST segments. Early repolarization is especially prevalent in young men, particularly young black men, and in athletes.^44^ Because the absolute risk of arrhythmic death is so low, asymptomatic individuals with early repolarization, even those with higher risk ECG patterns, do not require further evaluation except when there is a strong family history of sudden cardiac death or when the J point elevation is associated with Brugada syndrome (discussed later in this document) or short QT syndrome (SQT).

#### Ambulatory electrocardiographic monitoring

In 1984, Bigger *et al*. showed that ventricular ectopy recorded on a Holter monitor, especially when combined with a low left ventricular ejection fraction (LVEF), predicted a higher risk of mortality in post-infarction patients compared to those without ectopy.^45^ Non-sustained VT is also associated with increased risk in patients with arrhythmogenic and hypertrophic cardiomyopathy (HCM). Other data that can be extracted from ambulatory monitoring include heart rate, heart rate variability, and heart rate turbulence measurements, which can predict mortality risk at least in ischaemic cardiomyopathy (ICM), but have not been incorporated into clinical practice.^19^^,^^46^

### Imaging

**Table:** 

Imaging (echo, computed tomography (CT), magnetic resonance imaging (MRI), perfusion)	Class	References
Echocardiography should be used to evaluate EF for risk assessment for primary prevention of sudden cardiac death and the presence of structural heart disease. Alternatively, MRI or cardiac CT can be used.		^47^ ^,^ ^48^
Cardiac MRI is useful in assessing aetiology-driven risk of VT and for the presence of scar or myocardial inflammation.		^49–51^
Cardiac positron emission tomography may be useful for the assessment of aetiology-driven risk of ventricular arrhythmias and the presence of scar or myocardial inflammation in patients without CAD.		^52^ ^,^ ^53^

#### Risk assessment of ventricular tachyarrhythmia using imaging modalities

Evaluation for the presence of structural heart disease (SHD) is important for patients suspected of being at risk for sudden cardiac death. Left ventricular ejection fraction remains the key independent parameter for risk stratification of sudden cardiac death and to guide implantation of an ICD.^47^^,^^48^ Randomized controlled trials have shown a survival benefit from ICDs in patients with SHD and an EF ≤35%.^54–56^ Although EF is currently the only proven imaging modality demonstrated to risk stratify for sudden cardiac death, only 1–5% of patients with ICDs, implanted based upon a low EF, require therapies each year and the large majority of patients who receive ICDs will not have ICD therapies over the 3-year period after implantation.^57^^,^^58^ In addition, up to 70% of all sudden cardiac deaths in the community occur in individuals with EF >35%.^58–60^ Although the Efficacy of ICDs in Patients with Non-ischaemic Systolic Heart Failure (DANISH) trial showed that primary prevention ICD in the setting of severe non-ICM did not reduce all-cause mortality in patients on optimal medical therapy for heart failure, ICD implantation was associated with a 50% reduction in arrhythmic death. Of note, within this non-ICM population, younger patients (<68 years old) experienced a mortality benefit of 36% if treated with an ICD.^61^

Ejection fraction is most readily evaluated with echocardiography (recommendation level: green), given both lower cost, availability of equipment, and available expertise; however, cardiac MRI or CT can also be used to evaluate EF and SHD, particularly if obtained in combination of other assessment aims, such as CAD or if there is controversy over the quantified EF with echo (recommendation level: green). The imaging modality used to estimate EF has not been shown to determine benefit from ICD.^48^

Additional parameters beyond EF remain to be tested in large studies. Cardiac MRI with late gadolinium enhancement (LGE) can provide important prognostic information and may allow for more accurate assessment of scar. Presence and location of scar can portend a higher risk of sustained VT.^49–51^^,^^62^^,^^63^ In a study of 452 non-ICM patients with New York Heart Association Class II or II and EF <35%, ICD implantation was only associated with reduced mortality in the population that had presence of scar on cardiac MRI.^64^ Cardiac positron emission tomography (PET) may elucidate areas of inflammation which may identify inflammatory cardiomyopathies and sarcoidosis, a condition that is associated with higher risk of ventricular arrhythmias in patients without CAD (increased F-2-fluorodeoxyglucose uptake) or can be used to identify sympathetic denervation (carbon-11-metahydroxyephedrine imaging) or regions of inflammation. Greater sympathetic denervation on PET in a prospective study of ICM patients was a better predictor of ICD shocks than EF.^65^ Uptake of iodine-123 meta-iodobenzylguanidine (MIBG) to evaluate heart to mediastinum ration (H/M ratio) has shown mixed results in predicting arrhythmic death with some studies suggesting additional prognostic benefit for this parameter, while others have not demonstrated additional value.^66^^,^^67^ Importantly, the value of these additional parameters in determining risk of sustained VT, VF, or benefit from ICD in various population remains to be clarified. Finally, routine use of viability assessment using PET to guide revascularization in order to reduce risk of SCD remains an area of investigation. In patients with an EF <35% and CAD amenable to revascularization, routine use of PET to guide revascularization was not beneficial in reducing overall mortality.^68^

#### Imaging modalities for atrial arrhythmias

Echocardiography (transthoracic or transoesophageal) is a valuable tool in patients who present with atrial arrhythmias, specifically atrial flutter and AF, to evaluate for the presence of structural heart disease, left atrial enlargement, and valvular heart disease in order to better define treatment options. Cardiac MRI or CT may also be used if images obtained at echocardiography are not reliable. However, routine use of echocardiography, including atrial strain or atrial function in patients who do not have atrial arrhythmias to assess risk for the development of AF or atrial flutter is not warranted, unless other structural cardiac abnormalities are suspected.

### Invasive electrophysiological study

**Table:** 

Invasive electrophysiological study (EPS)	Class	References
EPS is indicated in patients with syncope and previous myocardial infarction, or other scar-related conditions when syncope remains unexplained after non-invasive evaluation.		^69^
EPS may be considered in patients with syncope and asymptomatic sinus bradycardia, in a few instances when non-invasive tests (e.g. ECG monitoring) have failed to show a correlation between syncope and bradycardia		^70–72^
EPS may be considered in patients with EF ≤ 40%, without a primary prophylactic ICD indication, and non-sustained VT in ICM (MUSTT criteria) to ascertain the presence of sustained VT events.		^73^
EPS may be helpful in patients with syncope and presence of a cardiac scar, including those with a previous myocardial infarction, or other scar-related conditions, when the mechanism of syncope remains unexplained after non-invasive evaluation.		^66^ ^,^ ^70^ ^,^ ^71^ ^,^ ^73^
EPS may be considered in patients with syncope and bifascicular block, when the mechanism of syncope remains unexplained after non-invasive evaluation.		^67^ ^,^ ^70^ ^,^ ^71^ ^,^ ^74^
EPS may be considered for risk stratification of SCD in patients with tetralogy of Fallot who have one or more risk factors among LV dysfunction, non-sustained VT and QRS duration exceeding 180 ms.		^67^ ^,^ ^70^ ^,^ ^71^ ^,^ ^74^
EPS may be considered in patients with congenital heart disease and non-sustained VT to determine the risk of sustained VT or identify SVT that could be ablate.		^67^ ^,^ ^70^ ^,^ ^71^ ^,^ ^74^
EPS may be considered in asymptomatic patients with spontaneous type 1 Brugada ECG pattern, or drug-induced type 1 ECG pattern and additional risk factors.		^75–77^
EPS is not recommended for additional risk stratification in patients with either long or short QT, catecholaminergic VT or early repolarization.	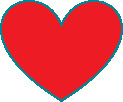	^70^ ^,^ ^71^
EPS is not recommended for risk stratification in patients with ischaemic or non-ischaemic DCM who meet criteria for ICD implantation.	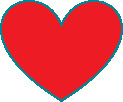	^70^ ^,^ ^71^

Currently, there are a few indications to perform an electrophysiological study (EPS) to further assess the risk of arrhythmias in at-risk cardiac patients. Such patients include those with structural heart disease, LVEF >35%, pre-syncope, syncope, palpitations, or markedly abnormal ECG suggesting severe conduction disease. These patients can be considered for an EPS to assess the risk of ventricular arrhythmias and sudden cardiac death to decide on need of an ICD, or to identify conduction disturbances or supraventricular tachycardias that can be treated with ablation or pacing.^70^^,^^71^

Patients withICM without a primary indication for an ICD, EF ≤40%, and non-sustained VT on ambulatory cardiac monitoring are candidates for an EPS according to the findings in the MUSTT trial,^73^ in which, 35% of patients with inducible sustained VT had a significantly lower risk of death with an ICD.^66^ The MADIT trial initially also utilized an EPS in post-MI patients with an EF ≤30%, and non-sustained VT events to implant an ICD, and showed survival benefit with the ICD.^54^ However, MADIT-II subsequently eliminated the need for an EPS in post-MI patients with an EF ≤30% and similarly showed the life-saving benefit of the ICD in a broader patient cohort.^55^ Therefore, post-MI patients with an EF ≤30% do not currently need to undergo an EPS to guide decisions on whether to implant an ICD.

In patients with heart failure and EF ≤ 35%, an EPS is not recommended for risk assessment for the decision on ICD indication. Some centres perform an EPS for inducibility to better characterize induced, sustained VT events, and their response to anti-tachycardia pacing, which may potentially help to tailor ICD programming. Furthermore, in patients who have syncope of uncertain origin, an EPS could identify ventricular arrhythmias or document electrical conduction disorders.^67^^,^^70^^,^^71^^,^^74^

In the case of channelopathies, there is no indication for an EPS, except for Brugada syndrome. In Brugada syndrome, EPS may be useful in asymptomatic patients with spontaneous or drug-induced type 1 pattern, especially when there is a family history of sudden death.^75–77^

### Implantable loop recorders

**Table:** 

Implantable cardiac devices	Class	References
An ILR is indicated in the evaluation of patients with infrequent recurrent syncope of uncertain origin especially when ambulatory monitoring is inconclusive.		^78–80^
An ILR is indicated in patients with syncope and high-risk criteria in whom a comprehensive evaluation did not demonstrate a cause of syncope or lead to a specific treatment, and who do not have conventional indications for primary prevention ICD or pacemaker.		^78–80^
An ILR can be considered in patients with palpitations, dizziness, pre-syncope, frequent premature ventricular complexes (PVCs)/non-sustained VT, and in those with suspected AF, and following AF ablation.		^78–80^

#### Implantable loop recorder to diagnose unexplained syncope/atrial fibrillation with cryptogenic stroke

The implantable loop recorder (ILR) provides long-term continuous monitoring and improves the diagnosis in patients with unexplained syncope.^81^ In a meta-analysis of 49 studies that included 4381 participants, the diagnostic yield for the detection of arrhythmogenic syncope was 26.5%.^78^ Moreover, the CRYSTAL-AF trial^80^ revealed that the ILR can detect subclinical AF following cryptogenic stroke. Still, any benefit of these findings needs to be confirmed in large randomized trials. Early use of the ILR has been advocated by the European guidelines^82^ and in the American guidelines following inconclusive non-invasive monitoring.^83^ The indications for ILR have been expanded in the current guidelines (*Table 2*).

**Table 2:** High-risk and low-risk criteria for syncope at initial evaluation (Adapted from 2018 ESC Guidelines for the diagnosis and management of syncope^82^)

Syncopal events
Low-risk
Associated with prodrome typical or reflex syncope (e.g. light-headedness, feeling of warmth, sweating, nausea, vomiting)
After sudden unexpected unpleasant sight, sound, smell, or pain^a^
After prolonged standing or crowded, hot places
During a meal or postprandial
Triggered by cough, defaecation, or micturition
With head rotation or pressure on carotid sinus (e.g. tumour, shaving, tight collars)
Standing from supine/sitting position
High-risk
Major
New onset of chest discomfort, breathlessness, abdominal pain, or headache
Syncope during exertion or when supine
Sudden onset palpitation immediately followed by syncope
Presence of structural heart disease especially left ventricular dysfunction and/or history of myocardial infarction
Minor (high-risk only if associated with structural heart disease or abnormal ECG):
No warning symptoms or short (<10 s) prodrome
Family history of sudden cardiac death at young age
Syncope in the sitting position

a Sudden loud sounds (as an alarm clock) may trigger VF in some long QT syndrome patients.

ECG, electrocardiogram; VF, ventricular fibrillation.

#### Implantable loop recorder to diagnose atrial and ventricular arrhythmia events

While the ILR can be useful to detect atrial and ventricular arrhythmias, a large cohort study indicated that most of the current use of ILRs is primarily in patients with unexplained syncope (84%), followed by palpitations (13%), and suspected AF (12%).^79^ Another smaller study specifically studying the risk of SCD and arrhythmias in patients with haemodialysis, found that 20% of these patients had SCD or bradyarrhythmia events necessitating pacemaker implantation, and 33% of these patients had an arrhythmic endpoint. Interestingly, the median time to event was 2.6 years, confirming the need for long-term monitoring. Surprisingly however, bradyarrhythmias were very commonly diagnosed in this cohort suspected to be at high risk for ventricular arrhythmias and sudden cardiac death.^84^ Further studies are needed to establish the role of ILR in risk stratification.

### Wearables/direct to consumer

**Table:** 

Wearables/direct to consumer	Class	References
Wearables may provide diagnostic data that contribute to disease detection and management when integrated into the clinical context and physician judgement.		^85^ ^,^ ^86^

The direct to consumer or wearable technology market, comprised of devices that monitor physiological parameters such as heart rate and sleep pattern, is anticipated to grow to 929 million connected devices by 2021.^87^ These devices encompass wristbands, glasses, in-ear monitors, chest straps, and smart phone-enabled recording electrode systems or electronic shirts, with varying capacity to monitor heart rate, heart rhythm, blood pressure, physical activity, respiratory rate, blood glucose, and sleep patterns.^88–90^ For heart rate monitoring, most wearable devices use photoplethysmography (PPG) technology, meaning they are inherently less accurate than conventional electrocardiography monitoring techniques. Accuracy of various devices varies, with correlation to reference standard ECG monitoring ranging from 0.76 to 0.99.^91^ Recent advances in wearable ECG acquisition include use of direct electrode recording that represents a regulatory approved medical device generating a lead I like rhythm strip, blurring the lines between consumer and medical devices.^92^

A growing body of evidence suggests that these technologies can be harnessed to facilitate arrhythmia detection in the appropriate context. Although marketed as consumer devices, many wearable devices may generate health data comparable to that of medical grade ECG monitors, with several devices migrating to approved medical use.^85^ Despite this promise, there are clear concerns regarding accuracy, particularly false positives in asymptomatic patients where device-based alerts can raise unwarranted concern and generate low yield screening for disease, with associated costs. Wearable technologies represent an important frontier in health evaluation, with the potential to provide readily accessible health data for large segments of the population, including those not captured by conventional monitoring techniques. Though intended for personal use focused on health promotion and physical activity, wearable technologies promise to invert the traditional paradigm of healthcare delivery, with data collection and health queries often initiated by consumers and not providers. Providers may see wearables as accessible risk stratification tools for detection of AF in high-risk cohorts (such as high CHADS_2_-VASC_2_ score patients), and patients may equally present for evaluation after device-based observations that call into question whether they are at risk. The confluence of these factors is illustrated in the recently presented Apple Heart Study, wherein 419 297 participants were recruited in only 8 months to participate in an AF screening study that deployed a PPG-based algorithm followed by a 7-day patch if AF was suspected.^93^ Using a complex tachogram algorithm, 2126 individuals were sent irregular pulse notifications and prompted for a telemedicine visit and 7-day ECG patch. The authors reported a PPV of 84% for each irregular pulse notification, and 71% for each irregular tachogram. The burden of notifications and the performance of the technology showed promise to inform AF detection in the broader public. Similarly, the Huawei Heart Study evaluated 187 912 individuals that used smart devices to monitor their pulse rhythm, with notification of suspected AF in 424 participants, with a strong relationship between advancing age and detecting AF. The predictive value of the algorithm in the 62% of notified participants that pursued medical evaluation was promising (87%).^94^

Studies evaluating PPG-based wearables in conjunction with machine-learning algorithms have shown promise in arrhythmia detection, such as AF.^86^ Studies to date have not focused on ventricular arrhythmia detection. Future wearables will benefit from improved reliability and accuracy, collect additional health and fitness parameters, support chronic disease management, and provide real-time connectivity and feedback that may supplant conventional medical monitoring. Wearables have the potential to become truly disruptive in our healthcare sector, with large segments of the population accessing cardiac monitoring that the physician must interpret. Currently, we have no data on how the information provided by PPG-based wearables will affect management and outcomes of patients, or how risk scores derived in other populations such as the CHA_2_DS_2_VASc score apply in these previously undetected subjects.

### Biomarkers, tissue, genetics

**Table:** 

Biomarkers, tissue, genetics	Class	References
Genetic testing should be considered in several inherited arrhythmic diseases associated with an increased risk of ventricular arrhythmia and SCD.		^95–97^
MRI with LGE to detect fibrosis and scar may be useful in assessing the risk of arrhythmic events in AF patients and patients with cardiomyopathies.		^98–100^
Plasma NT-proBNP may be useful in differentiating patients with higher vs. lower burden of AF.		^101–105^
Plasma CRP or other inflammatory markers may be useful in risk assessment, for identifying individuals with increased risk of future AF and for identifying individuals with high degree of atrial fibrosis.		^106–108^

The use of biomarkers, tissue biopsy, and genetic assessment can be used for risk assessment in patients suspected of specific arrhythmias or syndromes. The utility of using these tools broadly spans determining arrhythmic risk, refining a clinical diagnosis and estimating prognosis.

#### Biomarkers

Cardiac myocytes express and secrete natriuretic hormones that have a central function on blood pressure regulation, blood volume, and plasma sodium balance. Levels of B-type natriuretic peptide (BNP) and its stable N-terminal peptide pro-BNP (NT-proBNP) are increased in AF.^101^ AF burden has been shown to be associated with increased NT-proBNP.^102^ In a large meta-analysis consortium, BNP and C-reactive protein (CRP) associate with AF but only BNP was superior to well-known clinical variables in AF risk prediction.^103^ Inflammatory processes and fibrosis are central to pathogenesis of AF,^106^^,^^109^ and the inflammatory marker CRP is associated with longer AF duration and atrial remodelling.^110^ CRP levels are elevated in patients with permanent AF compared to persistent AF patients and are predictive of recurrent AF after catheter ablation,^111^^,^^112^ indicating that CRP levels can be used to identify AF subtypes and evaluate prognosis. Higher levels of CRP correlated to an increased risk of developing AF in general and after acute myocardial infarction.^107^^,^^113^ Similarly, the plasma protein YKL-40 may have diagnostic and prognostic use in AF patients^108^ because plasma serum chondrex (YKL-40) is associated with atrial fibrosis severity in patients with lone AF.^114^ Patients who experience recurrent AF following ablation have significantly increased YKL-40 baseline levels, although plasma YKL-40 is not an independent predictor of recurrent AF.^108^^,^^115^ Increasing levels of YKL-40 have been shown to associate with a two-fold increased risk of future AF.^116^ Other simple AF biomarkers include body weight and blood pressure, which are also major intervention targets.^117–122^

#### Tissue diagnostics

Tissue diagnostics can be beneficial to differentiate various infiltrative myopathic processes that can contribute to the risk for arrhythmic events. Fibrosis and scarring are well-recognized substrates for arrhythmia both in atria and ventricles.^109^ Fibrosis may be assessed in atria as well as in ventricular myocardium and its quantification can be used in evaluating the risk of arrhythmia in AF and cardiomyopathies.^98^^,^^99^ Specific patterns of scarring can assist in refinement of the diagnosis for infiltrative myopathies, hypertrophic cardiomyopathy, sarcoidosis, ACM, and amyloidosis. The development and validation of advanced imaging techniques including bio-metabolic imaging (sarcoid), and contrast enhanced cardiac MRI (amyloid) have largely replaced the need for invasive diagnostics.

#### Genetics

The majority of clinically applicable genetic testing is intended to be driven by phenotype and the pre-test probability of specific diagnosis determines the utility of genetic investigation.^95^ Due to incomplete penetrance of genetic arrhythmia syndromes, harbouring a genetic variant with known pathogenicity is almost never solely enough to meet diagnostic criteria for a particular syndrome.^123^

For LQTS, part of the diagnostic framework (along with the ECG biomarker of QT prolongation) can include a positive genetic test.^123^ Moreover, understanding the genetic diagnosis is important for treatment and prognostication. For example, patients with Jervell and Lange-Nielsen and Timothy Syndrome patients (LQT8) have more malignant clinical courses,^124^^,^^125^ and for LQT1 the arrhythmic risk depends partly on which region of the channel the mutation affects.^126^ In catecholaminergic polymorphic ventricular tachyarrhythmia (CPVT),^127^ genetic testing of suspected individuals has a moderately high yield.^95^ Identification of an at risk first-degree relative of a CPVT affected individual is essential due to the high penetrance but more so the lethality of this syndrome.^123^^,^^128^ Similar to LQT1, CPVT due to RYR2 mutations may have some degree of risk depending on where in the ryanodine receptor the mutation falls.^129^ Brugada syndrome can be particularly difficult to clinically diagnose and the utility of genetic testing for improving diagnosis is poor. For patients who are clinically diagnosed with Brugada Syndrome the yield of genetic testing is ∼30%,^130^ the majority of whom harbour SCN5a mutations, a gene associated with a plethora of arrhythmia syndromes.^131^^,^^132^ Genetic testing can be useful for family members of an appropriately genotype identified proband but is not recommended in the absence of a diagnostic ECG.^95^ Using genetics as part of diagnostic criteria for arrhythmogenic cardiomyopathies will be discussed later in the document. Lastly, genetics in AF is a developing area, but certain primary electrical sudden death syndromes have increased AF association as discussed in Patients with inherited rhythm disease (long QT syndrome/short QT syndrome/catecholaminergic polymorphic ventricular tachyarrhythmia/Brugada syndrome) section. For families with a substantial number of AF cases or in early onset AF, genetic testing can be considered but the yield is low.^133–136^

### Artificial intelligence

Machine learning is a broad term of artificial intelligence derived from the extraction of patterns from large data sets. The marriage with healthcare analytics and decision processes has been rapidly forwarded with computerized medical records and the creation of large data warehouses.

A deep neural network was created to analyse raw ECG data from an ambulatory heart monitor and classify it into 12 categories based upon the presence of arrhythmia. Machine learning performed very well with an average under the reviewer operating characteristic curve (ROC) of 0.97 and an average F1 score (mean of the PPV and sensitivity) of 0.837; a score better than an average cardiologist (0.780).^137^

Machine learning has been applied to standard ECG characteristics in sinus rhythm to predict incident AF using the eight independent ECG leads (leads I, II, V1–6) through a convolutional neural network.^138^ The ROC area under the curve for the detection of AF was 0.87 (0.86–0.88) using the internal validation dataset and 0.87 (0.86–0.88) using the testing dataset.

In an analysis of the Atrial Fibrillation Prediction Database, a machine learning approach based upon heart rate variability predicted onset of AF with sensitivity of 100%, specificity of 95.6%, and accuracy of 96.2%.^139^ Machine learning based upon ECG characteristics identified left ventricular dysfunction with an area under the curve of 0.93, sensitivity of 86.3%, and specificity of 85.7% including risk of left ventricular dysfunction in those without.^140^

Machine learning has shown accuracy in predicting mortality and risk stratification of patients with CAD.^141^ Machine learning has also been shown to accurately discriminate between athletic hearts compared to hypertrophic cardiomyopathy hearts.^142^ Machine learning has great potential in this area of risk assessment because of the large amount of data contained in the large ECG and clinical datasets available to determine rules.

## How to assess risk for atrial fibrillation in specific populations

### Patients of advanced age

**Figure 1:**
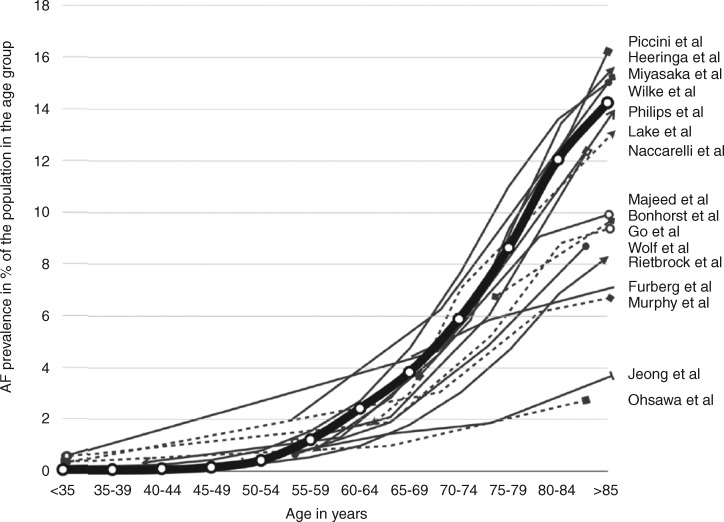
A depiction of the atrial fibrillation prevalence distribution found by each study published to date.^151^ This depiction uses the sex-specific average rates of AF prevalence, grouped by age. The thick line represents average AF prevalence rates by age group, as derived from a pooled analysis of the individual studies weighted by sample size. (Adapted from Andrade *et al. Circ Res* 2014.) AF, atrial fibrillation.

There is agreement that the prevalence of AF in the general population in the Western world is in the order of 1–2%.^143–145^ It is estimated that in 2010 there were 33.5 million people in the world with AF of which 20.9 million were men and 12.6 million were women.^146^ During the past 20 years, the age-adjusted prevalence rates of AF increased for both men and women and similarly the corresponding incidence rates have increased.^146–150^ Age is a major risk factor for the development of AF and in persons younger than 55 years a prevalence of AF around 0.5% is seen whereas in persons older than 85 years AF prevalence is around 15% (*Figure 1*).^144^ A stepwise increase in AF prevalence with increasing age has been found in several studies.^152^^,^^153^ Studies in a multi-ethnic cohort from the United States has shown large variation in AF prevalence among various race-ethnicity groups in which AF associated hospitalizations were lower in Hispanics, Chinese, and Black Americans compared to White Americans.^153^ The predominant contributor to the increasing AF prevalence is our aging populations, more widespread use and availability of screening tools, and improved treatment for various heart diseases that enhance longevity.

Among AF patients, those aged younger than 65 years are in general healthier than those older than 65 years.^154^ Life time risks of AF in 55-year-old subjects without a history of AF have been found to be 20–24% in the Rotterdam study^155^ but considerably higher at 37% in the Framingham study.^134^ The lifetime risk of AF in Asians older than 20 years (1 in 6 for men and 1 in 7 for women; i.e. 14–17%) was lower than the risk reported from Western countries.^156^

**Figure 2:**
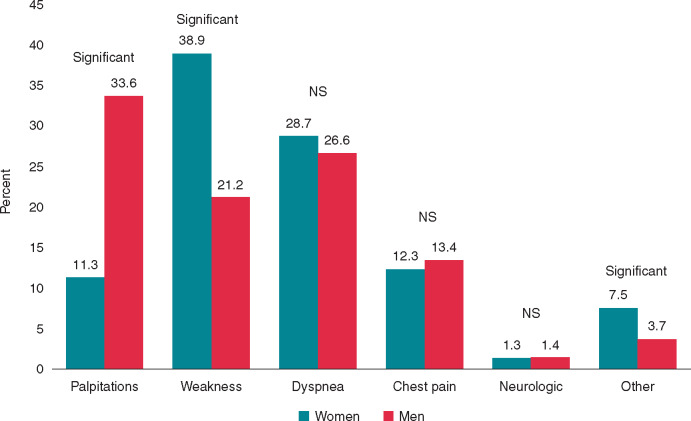
Sex differences in symptoms related to atrial fibrillation^144^ (Adapted from Andrade *et al. Can J Cardiol* 2018).

The incidence rates, prevalence, and lifetime risk of AF are higher for men than women. Despite this, the absolute number of women with AF exceeds the total number of men with AF because women live longer than men.^144^ Women have their first episode of AF about 5 years later than men and less commonly have lone AF.^144^ In general, women with AF are more likely to have hypertension or valvular heart disease compared to men.^144^ Women often present with atypical symptoms related to AF (*Figure 2*). On the other hand, compared to men, women are less likely to have asymptomatic AF, they have a higher symptom burden, they have higher average heart rate during AF and more often longer lasting episodes of AF.^144^ These factors contribute to the observation that women are more likely to contact their physician due to AF-related symptoms compared to men.

Conflicting results exist with respect to risk of stroke secondary to AF and its prognosis in women compared to men.^157–159^ There does not seem to be a gender difference with respect to development of dementia secondary to AF, although women have higher rates of dementia than men in general.^145^^,^^157^

Since both AF and stroke are highly associated with age and stroke may occur as a complication of AF it seems reasonable to consider screening for this arrhythmia in elderly populations. Several studies are ongoing and expected to be finalized within the next couple of years. These studies are expected to guide us with respect to cost-effectiveness of these screening strategies.

### Patients with heart failure

**Table:** 

Investigations needed to assess risk for AF in patients with heart failure	Class	References
A careful evaluation of clinical characteristics known to be associated with increased risk for AF should be performed.		^160^
Frequent interrogation or remote monitoring of stored arrhythmia episodes in device implanted HF patients should be performed in order to diagnose AF and allow its early management.		^161^
Echocardiography is useful in identifying cardiac characteristics associated with a higher risk for AF.		^162^
Cardiac MRI may be considered in identifying degree of atrial fibrosis and scar.		^163^
Use of biomarkers may be considered for identifying individuals with increased risk of future AF and for identifying individuals with high degree of atrial fibrosis.		^107^ ^,^ ^164^ ^,^ ^165^
Searching for common genetic variants associated with AF risk by genetic molecular analysis has not been found to be useful in a routine clinical setting.	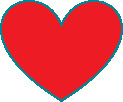	^166^

Due to common risk factors like age, hypertension, diabetes, obesity, and sleep apnoea, AF and HF are intricately linked and share common pathophysiologic mechanisms. Atrial fibrillation occurs in more than half of individuals with HF and presence of both carries greater mortality risk compared with those without either condition.^167^

In the particular case of cancer treatment, HF is also a common consequence of cardiotoxicity associated with some chemotherapeutic agents, including anthracyclines, human epidermal growth factor receptor 2 (HER2), and proteasome inhibitors. In this setting, isolated cases of AF have been reported. Even if the exact mechanism of these arrhythmias induced by such drugs remains largely unknown, it seems plausible that the negative effect on the cardiac systolic function also plays a central role.^168^

**Figure 3:**
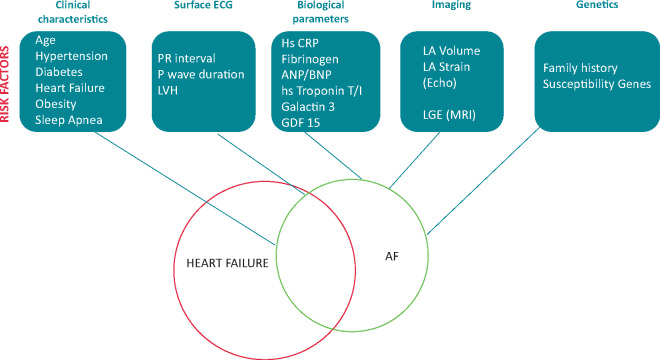
Investigations and associated risk factors useful to predict the development and progression of AF in HF patients. AF, atrial fibrillation; BNP, B-type natriuretic peptide; CRP, C-reactive protein; ECG, electrocardiogram; HF, heart failure; LA, left atrium; LGE, late gadolinium enhancement; LVH, left ventricular hypertrophy; MRI, magnetic resonance imaging.

Given the deleterious effects of AF in HF patients, significant interest has been directed to risk factors predicting the development and progression of this arrhythmia (*Figure 3*).

#### Clinical risk factors

Older age and male gender are associated with a higher risk of developing AF.^160^ Diabetes confers a 1.4- to 1.6-fold higher risk for AF.^160^ Because of its high prevalence in the general population, hypertension is responsible for more AF in the population (14%) than any other risk factor.^160^ Obesity and sleep apnoea are independent risk factors for AF.^169^ AF incidence also increases in case of renal or thyroid dysfunction.^170^^,^^171^

With regard to HF and the type of underlying heart disease, prevalence of AF increases significantly with the severity of HF symptomatology. Among the valvular diseases, the left-sided valve stenoses have the highest prevalence rates of AF. In addition, the presence of CAD or hypertrophic cardiomyopathy is a significant risk factor for incidence and progression of AF.^172^ Finally, in congenital heart disease patients, substantial AF rates appear decades before their onset in the general population.^173^

#### Electrocardiography

Electrocardiogram-derived variables, such as the PR interval, ECG-based left ventricular hypertrophy (LVH), P wave indices like P wave duration, area, and terminal force have been used in various AF prediction models but their additive value over other clinical risk factors is minimal.^174^ Short duration Holter monitoring is not useful for AF detection in asymptomatic patients. Longer duration monitoring with external or implantable loop recorders may help when paroxysmal AF is suspected. In addition, frequent interrogation or remote monitoring of Holter memories in device implanted HF patients is mandatory in order to diagnose AF and allow its early management.^161^

#### Biomarkers

Markers of inflammation (high-sensitivity CRP, fibrinogen), atrial overload (atrial and B-type natriuretic peptides), myocardial ischaemia (high-sensitivity troponin T and I), cardiac fibrosis (galectin-3), and others (soluble ST2, growth differentiation factor-15), have been studied to predict AF incidence.^165^ Of these, only natriuretic peptides have consistently demonstrated added predictive value beyond information on clinical variables.^164^^,^^165^

#### Imaging

Many echocardiographic variables have been associated with a significantly higher AF recurrence rate. Possibly, left atrial volume is superior to left atrial diameter in predicting progression to persistent AF. Speckle left atrial strain and stiffness index can also predict the maintenance of sinus rhythm after cardioversion for AF.^162^

Concerning MRI, the amount of left atrial enhancement quantified on MRI with LGE may be helpful to predict progression of AF,^163^ but the reproducibility of such findings remains controversial.

#### Genetics

A family history of AF in a first-degree relative independently increases AF risk two-fold.^175^ Recent research has identified several common genetic variants associated with the risk of AF.^136^ Further studies are required to evaluate whether genetic information improves our ability to predict AF on top of clinical variables.

Risk assessment of AF in patients with HF can be carried out at first by considering the clinical features, comorbidities, and underlying aetiologies. It can be further refined by more sophisticated investigations.

### Patients with obesity, hypertension, diabetes, sleep apnoea, or structural heart disease

**Table:** 

Patients with obesity, hypertension, diabetes, sleep apnoea, or structural heart disease	Class	References
Clinical risk factors should be assessed to help identify incident AF and its complications.		^176^
Clinical risk scores may be useful to identify risk for incident AF.		^177–179^

The assessment of underlying AF in people at higher risk for AF can be considered from opportunistic perspective, or the consideration of clinical risk prediction tools.^180^ Many patients with common conditions that may predispose to AF, such as obesity, sleep apnoea, hypertension, or SHD should or would be attending specialist clinics for their assessment and/or follow-up. Hence, an opportunistic strategy of pulse palpation and clinical assessment (e.g. symptoms) followed by appropriate ECG monitoring to confirm AF would be an appropriate and cost effective method for screening.^181^ In general, clinical scores have been less useful as most only have modest predictive value for identifying the population at risk; ultimately, these patients would also require their AF documented. A strategy of using risk scores to target high-risk patients for more intense screening efforts merits consideration.

The systematic review by Allan *et al.*^176^ found that in relation to the relative risk of incident AF:

- For every 1–10 kg/m^2^ increase in body mass index (BMI), or BMI ≥25–30 kg/m^2^, all 19 reports showed significant direct associations (from 1.04 [1.02–1.05] to 2.24 [1.41–3.58]).
- For every 10–22 mmHg increase in systolic blood pressure, or systolic blood pressure ≥160 mmHg, most reports showed significant direct associations (from 1.14 [1.05–1.25] to 2.63 [1.83–3.78]).
- For diabetes mellitus (type unspecified), eight reports showed a direct but non-significant (from 1.02 to 1.49) and six reports showed significant direct associations (from 1.17 [1.16–1.19] to 1.80 [1.30–2.60]).

Many of these conditions are present concomitantly. Also, obesity and hypertension are commonly associated with sleep apnoea, which is another risk for incident AF.

Obesity has been associated with incident AF,^182^ but clinical trial data have a suggestion of an ‘obesity paradox’ whereby overweight AF patients tended to have improved outcomes; however, the relationship between obesity and outcomes from real-world observational cohorts are less clear.^183–185^ In a systematic review of trial and real-world evidence, there was suggestion of an obesity paradox in AF patients, particularly for all-cause and cardiovascular death outcomes.^184^ An obesity paradox was also evident for stroke/systemic embolic event outcomes in the non-vitamin K antagonist oral anticoagulant (NOAC) trials, with a treatment effect favouring NOACs over warfarin for both efficacy and safety that was significant only for normal weight patients. Nonetheless, proactive management of obesity is part of the lifestyle advice for patients with AF.

On a population basis, hypertension is the most common aetiological factor for AF, and contributes to its complications. Indeed, AF can be regarded as a manifestation of hypertension target organ damage. The optimal blood pressure targets in AF patients have been described, being 120–129/<80 mmHg.^186^ Also, longer hypertension duration is associated with the increased risk of ischaemic stroke; however, this long-term effect of hypertension duration can be attenuated by long-term strict SBP control throughout the entire duration of hypertension.^187^

Poor diabetes control is associated with incident AF. In the diabetic AF patient, longer disease duration is related to a higher risk of stroke/thromboembolism in AF, but not with a higher risk of anticoagulant-related bleeding.^188^ These risks were similar for Type 1 and Type 2 diabetes.^189^ Evidence of other target organ damage such as diabetic retinopathy increased risk, although it did not add to the predictive value of risk assessment using the CHA_2_DS_2_-VASc score.^190^ Indeed, the ATRIA study also confirmed that duration of diabetes is a more important predictor of ischaemic stroke than glycaemic control in patients who have diabetes and AF.^191^

Unsurprisingly SHD is a potent risk factor for incident AF, as well as its complications, such as stroke and HF.^177^^,^^192^ Systolic HF is one of the components of the simple C2HEST score [Chronic obstructive pulmonary disease and CAD [1 point each]; hypertension [1 point]; elderly [age ≥75 years, 2 points]; systolic HF [2 points]; thyroid disease [hyperthyroidism, 1 point])] which has been derived and validated in a large cohort of AF patients.^177^ This score could potentially be considered to target the high-risk patients that may be suited for more intense screening for incident AF, e.g. post-stroke where the C2HEST score was superior to the other scores such as the Framingham score.^178^ The risks of AF with associated valvular heart disease are well recognized, as recently discussed in an EHRA position document.^193^ In terms of HF, there is a link between AF complications and HF, whether HF with a reduced EF (HFrEF) or HF with a preserved EF (HFpEF).^194^ In the CHA_2_DS_2_-VASc score, the ‘C’ component refers to recent decompensated HF, irrespective of the EF, or the presence of moderate-severe systolic dysfunction whether asymptomatic or not.^7^ Of note, the CHA_2_DS_2_-VASc score is predictive of stroke in HF, whether or not AF is present.^195^

### Patients who have undergone cardiac surgery

**Table:** 

Patients who have undergone cardiac surgery	Class	References
Heart rhythm monitoring for 4–7 days is recommended for detection of post-operative AF.		^196–198^
Patients with post-operative AF may undergo follow-up rhythm monitoring to assess for the presence of symptomatic and asymptomatic arrhythmias.		^196–199^

Post-operative AF remains the most common complication following cardiac surgery and its incidence ranges between 20–50% across numerous studies.^196^ This risk increases from isolated CABG surgery, to valvular surgery, and in turn to concomitant CABG/valvular surgery.

Risk factors for developing AF may be divided into procedural- and patient-related factors. Procedural-related risk factors include type of surgery, mitral valve surgery, use of intra-aortic balloon pump, longer cardiopulmonary bypass and aortic clamp times, and perioperative issues such as inflammation, infection, fluid overload, inotropic use, atrial ischaemia, hypokalaemia, and hypomagnesaemia. Patient-related risk factors include advanced age, history of AF, history of HF, renal failure, hypertension, chronic obstructive pulmonary disease, post-operative withdrawal or absence of beta-blocker, or angiotensin-converting enzyme inhibitor (ACE inhibitor) therapy.^197^^,^^200^ Left atrial remodelling predisposes to post-cardiac surgery AF, with risk factors such as enlarged left atrial size, diastolic dysfunction, LVH, obesity, obstructive sleep apnoea, and the CHADS_2_ and CHA_2_DS_2_-VASc score further predisposing to post-operative AF.^197^^,^^201^^,^^202^

The majority of post-cardiac surgical AF occurs within the first 4 post-operative days, and is most common on the 2nd post-operative day, while recurrences are most common on the 3rd post-operative day.^197^^,^^203^ In another study of CABG patients, 94% of post-operative AF occurred by the 7th post-operative day.^198^ Hence rhythm monitoring such as inpatient telemetry or ECG for post-operative AF should focus on this time frame.

While post-cardiac surgical AF likely occurs as a result of the interaction between acute perioperative triggers and the underlying atrial and cardiac substrate, its occurrence identifies a subset of patients associated with long-term morbidity and mortality. In a study of patients who underwent CABG, post-operative AF conferred an eight-fold increased risk of future AF and doubled cardiovascular mortality on long-term follow-up.^199^ Follow-up rhythm monitoring, for example with ECG or Holter monitoring is advisable in this subset of patients particularly in the setting of symptom development. There is emerging data on the use of implantable cardiac monitors for long-term monitoring of this subset of patients. While implantable cardiac monitors allow continuous long-term monitoring for arrhythmias and asymptomatic arrhythmias, the risk–benefit ratio is balanced by the arrhythmia detection rate beyond the immediate post-operative period and level of invasiveness of the monitoring device. Its routine use will depend on further results from prospective medium to long-term studies.

### Patients with cryptogenic stroke

**Table:** 

Patients with cryptogenic stroke	Class	References
Patients should initially undergo brain diffusion-weighted MRI imaging for the diagnosis of cryptogenic stroke.		^204^ ^,^ ^205^
AF is more likely to be detected after cryptogenic stroke with more intense investigation with longer and more sophisticated monitoring.		^205–207^
Long-term ECG monitoring techniques, such as trans-telephonic ECG monitoring or cardiac event recorders or ILR can increase yield of AF diagnosis after cryptogenic stroke in selected patients.		^205^ ^,^ ^206^
The use of an ILR should be considered for detecting AF in selected patients who are at higher risk of AF development, including the elderly, patients with cardiovascular risk factors or comorbidities.		^80^ ^,^ ^207^
TOE may lead to the reclassification of cryptogenic stroke because many cases are embolic and due to a cardiogenic source, mainly AF.		^205^ ^,^ ^206^

**Figure 4:**
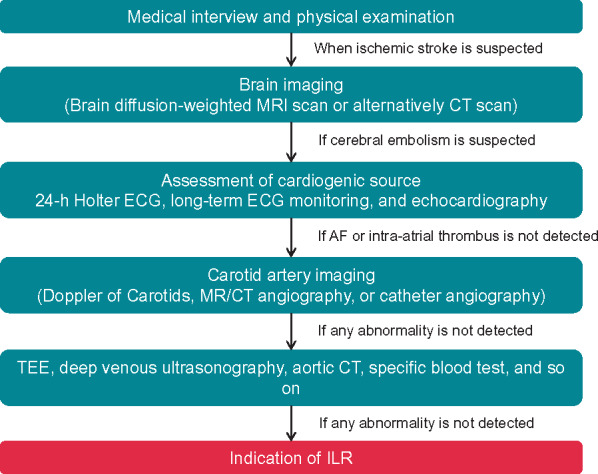
Proceeding of evaluation for cryptogenic stroke. AF, atrial fibrillation; CT, computed tomography; ECG, electrocardiogram; ILR, implantable loop recorder; MRI, magnetic resonance imaging; TOE, transoesophageal echocardiography.

Cryptogenic stroke is defined as ischaemic stroke of undetermined aetiology.^208^ The diagnosis of cryptogenic stroke is generally made by exclusion. Although cryptogenic stroke includes few potential causes, such as paradoxical embolism through a patent foramen ovale, atrial septal aneurysm, and aortic arch atheroma, the majority of cases are thought to be caused by cardio-embolism due to undetected paroxysmal AF.^205^ For the diagnosis of cryptogenic stroke or a suspected transient ischaemic attack (TIA), patients should initially undergo brain imaging. Diffusion-weighted MRI is more recommended than any other MRI sequence or CT as brain imaging, except when contraindicated.^204^^,^^205^ Advances in cardiac imaging techniques such as transoesophageal echocardiography (TOE) have prompted the reassessment of cryptogenic stroke because most cases are thought to be embolic due to a cardiogenic source, mainly AF. Transoesophageal echocardiography can easily detect a thrombus of the left atrial appendage, particularly with contrast enhancement, which cannot be detected using conventional transthoracic echocardiography. Transthoracic echocardiography with contrast could be useful to detect a left ventricular thrombus (*Figure 4*).

The detection of permanent or persistent AF is relatively easy, whereas that of paroxysmal AF is more difficult. Current guidelines recommend the use of ECG monitoring among patients with ischaemic stroke including cryptogenic stroke and TIA for whom transient (paroxysmal) AF is suspected and no other causes of stroke are identified.^205^^,^^206^ First, 24-h Holter ECG is performed to detect the AF burden. If undetected, other long-term ECG monitoring techniques such as trans-telephonic ECG monitoring or cardiac event recorders (a symptom event monitor or a ILR) may be attempted as alternative methods. A meta-analysis indicated that a longer duration of ECG monitoring is associated with an increased detection of new AF when examining monitoring time as a continuous variable. Studies with monitoring lasting ≤72 h detected new AF in 5.1% of cases, whereas monitoring lasting ≥7 days detected AF in 15% of cases.^209^ The proportion of new diagnosis of AF was increased to 29.1% with 3-months extended monitoring. Recently, smartphone-based ECG recording systems have been developed and conferred acceptable sensitivity and specificity of detecting AF^191^ (see Wearables/direct to consumer section).

The use of an ILR is indicated for detecting the presence of AF or arrhythmia burden that might cause ischaemic stroke in selected patients, for example those who are at higher risk of AF development including elderly, patients with cardiovascular risk factors or comorbidities. An ILR is a useful tool for detecting arrhythmias. In the CRYSTAL AF study, AF was newly detected in 8.9% of patients with an ILR by the 6th month compared with 1.4% among those receiving conventional ambulatory ECG monitoring, increasing further to 12.4% by 12 months compared with 2.0% in conventional monitoring.^80^ A similar outcome was observed in the EMBRACE trial, in which AF was newly detected in 16.1% of patients who received 30-day ILR compared with 3.2% who received ambulatory 24-h monitoring.^210^ A systematic review indicated that AF was newly detected in nearly one-quarter of patients with stroke or TIA by sequentially combining cardiac monitoring methods: 7.7% in phase 1 (emergency room), 5.1% in phase 2 (in-hospital), 10.7% in phase 3 (first ambulatory period), and 16.9% in phase 4 (second ambulatory period consisting of trans-telephonic ECG monitoring, cardiac event recorders, and ILR), and 23.7% in the overall detection after all phases of sequential ECG monitoring.^207^ Thus, if we ‘look harder, look longer and look in more sophisticated ways’ we are more likely to detect AF. It is possible that if we use clinical risk stratification (e.g. the C2HEST score) to identify patients post-stroke at high risk of incident AF, targeted intensive monitoring can be applied.^211^

### How to assess high risk of atrial fibrillation in professional athletes

**Table:** 

Atrial fibrillation in athletes	Class	References
In athletes who participate long term in endurance exercises with symptoms of arrhythmia screening for AF is recommended.		^212^
Risk assessment for AF risk in athletes may include the duration and intensity of exercise as a potential modifiable risk factor.		^213^ ^,^ ^214^

#### Atrial fibrillation risk in athletes—general

Paroxysmal or persistent AF is common in athletes and may be autonomically mediated or triggered by other supraventricular tachycardias.^215^ AF is the primary arrhythmia observed in middle-aged athletes.^216^ AF in athletes tends to be paroxysmal, vagally mediated, and highly symptomatic.^213^ The mechanism of increased AF risk at either end of the physical activity spectrum likely includes autonomic, structural, inflammatory, and fibrotic changes to the heart. For example, increased vagal tone, which is often observed in the endurance athlete, has been shown to result in a short atrial refractory period, and thus initiates AF.^217^

#### Atrial fibrillation risk in athletes—exercise paradox

Recent studies have observed a U-shaped risk relationship of physical activity to AF. At one end of the spectrum, a large observational study^218^^,^^219^ of people showed that those at the lowest levels of physical fitness had a 5-fold increased risk of AF.^220^ Increasing the physical activity of sedentary patients could help reduce the risk or burden of AF. Long-term endurance training, as well as a sedentary lifestyle,^221^ increase chronic systemic inflammation, which in turn could also facilitate AF.^106^ For example, one randomized study demonstrated that just 12 weeks of moderate-intensity physical activity decreased the AF burden by 41%.^222^ Of the physically inactive with AF, the obese might benefit the most from moderate levels of physical activity.^220^ In contrast, a meta-analysis of 655 endurance athletes also demonstrated a five-fold increased risk of AF.^212^ Of these studies, increased AF risk was generally only observed with the highest levels of physical activity that was maintained over a prolonged period of time.^213^^,^^214^ One uniform explanation for the exercise paradox is that both long-term endurance training and a sedentary lifestyle increase chronic systemic inflammation.

#### Atrial fibrillation risk in athletes—structural cardiac changes

Most studies have shown structural changes in endurance athletes, which have resulted in the term athlete’s heart. These changes include dilatation of all four heart chambers, increase in left ventricular mass, and mild right ventricular hypertrophy.^223^ Studies show that moderate physical activity might reduce inflammatory markers.^224–226^ Extreme levels of exercise are a known cause of cardiac fibrosis, particularly in hinge point locations of the heart, such as the right ventricle; however, the significance of MRI-detected fibrosis remains controversial.^227^ Athletes who experience higher levels of fibrosis also have higher levels of coronary calcium.^228^ In turn, fibrosis is a well-established risk factor of AF.^163^ In one study, the fibrotic changes caused by vigorous exercise were reversed after an 8-week period of physical activity cessation.^229^ Among young elite athletes, age, years of competition, and echocardiographically measured parameters, including left atrial anterior–posterior diameter and atrial strain, were associated with higher AF risk.^230^^,^^231^ Although increasing physical activity might reduce AF in sedentary patients, decreasing physical activity levels in elite endurance athletes may also reduce AF.^215^ Currently, the role of deconditioning to lower AF risk in elite athletes for primary or secondary prevention of arrhythmia requires prospective evaluation.

### Patients with inherited rhythm disease (long QT syndrome/short QT syndrome/catecholaminergic polymorphic ventricular tachyarrhythmia/Brugada syndrome)

**Table:** 

Patients with inherited rhythm disease	Class	References
Patients with certain inherited arrhythmia syndromes are at higher risk for AF and benefit from symptom-driven and periodic surveillance.		^123^
Evaluation should include non-invasive symptom-driven surveillance for patients at risk for AF and periodic non-invasive surveillance for asymptomatic patients.		^232–234^
EPS to determine atrial AF substrate or susceptibility is not useful.	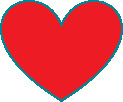	^123^

Some patients with primary electrical sudden death syndromes have an increased AF association, including Brugada Syndrome, LQTS, SQT, and catecholaminergic polymorphic ventricular tachycardia (CPVT). These patients are at risk for arrhythmia symptoms from AF and are vulnerable to AF consequences such as pro-arrhythmia and inappropriate ICD shocks.

Brugada Syndrome is characterized by ST-segment elevation in the precordial ECG leads and increased risk of SCD due to VF.^235^ Brugada Syndrome is associated with a higher incidence of SVTs, and AF is the most common SVT in these patients.^236^^,^^237^ AF susceptibility has been described with patients harbouring mutations in SCN5A, CACNA1C and patients without an identified genotype,^234^^,^^238^ suggesting a lack of genetic AF specific risk but AF may be more prevalent with more advanced disease.^239^^,^^240^ Importantly, AF events can be pro-arrhythmic for Brugada Syndrome patients^123^^,^^241^ and contribute to the high inappropriate ICD shock rates for Brugada Syndrome patients.^241^

Long QT syndrome is a genetically heterogeneous syndrome associated with mutations in 17 different genes with some unique phenotypic characteristics based on genotype and electrically results in prolonged repolarization and risk for fatal ventricular arrhythmia torsade de pointes. While generally, prolonged repolarization inhibits AF initiation and this is the mechanism for Vaughn–Williams Class III anti-arrhythmic drugs, rare patients with LQTS have also been noted to have AF.^242^^,^^243^ This has been limited to single case reports and unverified, 1.7% of patients in a LQTS cohort, which is a higher prevalence than the general population.^133^^,^^244^ Not surprisingly, some genes associated with AF in LQTS have overlap with familial AF: LQT1 (KCNQ1), LQT2 (KCNH2), LQT3 (SCN5a), and LQT7 (KCNJ2). However, for potassium channels, in LQTS the genetic defect results in ‘loss of function’ in contrast to a ‘gain of function’ in familial AF.^245^^,^^246^ It is less clear how prolonged repolarization results in AF susceptibility but it may involve similar mechanisms to torsade de pointes^247^ or perhaps dispersion of repolarization and induction of early afterdepolarizations.^248^^,^^249^

From an electrical substrate standpoint, it is easier to understand why SQTS and CPVT are associated with AF. Short QT syndrome is a rare disorder caused by a gain of function of potassium channels encoded by KCNQ1, KCNH2, and KCNJ2, causing a shortening of the action potential and manifests in the atrium by a decreased atrial refractory period and electrical substrate for AF.^250–252^ CPVT is an autosomal dominant disorder associated with polymorphic VT and bidirectional VT due to cellular calcium overload caused by mutations in calcium handling genes.^253–255^ A reciprocal condition can exist in the atria of patients with CPVT with AF susceptibility and has been shown to be more prevalent in patients with more dysfunctional ryanodine receptor2 channels.^256^ It is also unclear how clinically significant AF is for CPVT patients. However, the failure to recognize and treat AF can result in inappropriate shocks, pro-arrhythmia, and death.^232^^,^^233^

These issues highlight the need for AF recognition, ICD programming to reduce the risk of inappropriate shocks, and preventative treatment. Because of the small cohort sizes and lack of systematic studies, it is difficult to prospectively estimate AF risk. Invasive EP studies evaluating atrial refractory periods, conduction time, and AF inducibility have been inconclusive^236^^,^^237^ and either not systematically evaluated in large populations or are contraindicated (LQTS and CPVT).^123^ We support vigilant non-invasive surveillance in these conditions. For patients with ICD, close follow-up is needed to decipher and to adjudicate if atrial arrhythmias are present and proactively increase the rate cut-off for VF detection and turn SVT discriminators on, if available. Patients without ICD, but suggestive symptoms, should undergo ambulatory monitoring and asymptomatic patients should have surveillance monitoring done every 1–2 years. Treatment is not the focus of this article, but it should be recognized that many AADs can worsen the electrical substrate for inherited arrhythmia patients (i.e. LQTS, Brugada Syndrome) and care should be taken when choosing antiarrhythmic drugs.

## How to assess risk for adverse outcomes in patients with atrial fibrillation

### Risk assessment for stroke/transient ischaemic attack/cognitive decline

**Table:** 

Risk assessment for stroke/TIA/Cognitive decline	Class	References
A risk factor-based approach is recommended for stroke risk assessment in patients with AF.		^8^ ^,^ ^257^
Cognitive assessment should be performed in AF patients where there is suspicion of cognitive impairment.		^258^ ^,^ ^259^
Assessment of cognitive function may be multifaceted, and cognitive impairment screening by available tools is just one component.		^258^
Risk reduction of cognitive dysfunction and its comorbidities in AF may include risk assessment for vascular disease and/or Alzheimer’s disease.		^258^ ^,^ ^260^
General health measures may reduce the concomitant risks of AF and stroke, with a putative benefit on cognitive function.		^1^ ^,^ ^2^

Patients with AF have increased mortality and morbidity compared with non-AF patients and may experience significant adverse events. Stroke and thrombo-embolic events are well known complications that can be avoided by oral anticoagulation. Since the risk of individual patient differs significantly, an individual risk assessment is necessary. Several stroke risk scores, including ABC-stroke (age, biomarker, clinical history), ATRIA (Anticoagulation and Risk Factors in Atrial Fibrillation), GARFIELD (Global Anticoagulant Registry in the FIELD), and Qstroke have been proposed as support tools for the decision on oral anticoagulation.^261–264^ However, the one currently most widely applied and recommended by international guidelines is the CHA_2_DS_2_-VASc risk scheme. According to CHA_2_DS_2_-VASc, patients with score of ≥1 in a male or ≥2 in a female should be considered for stroke prevention strategies.^265–268^ Nevertheless, it has to be kept in mind that no stroke risk scheme has perfect predictive accuracy.

Another major adverse effect of AF is impairment of cognitive function.^258^^,^^259^ Multiple risk factors for dementia have been identified in the general population, including modifiable and non-modifiable ones.^269^ Apart from these AF-non-specific risk factors, AF may lead to cognitive impairment by multiple mechanisms. These include apparent stroke, silent stroke but also other mechanisms that are independent of thromboembolism.^270^ A detailed description of the association between AF and cognitive impairment and possible preventive mechanisms has been provided recently in an expert consensus document.^258^ In terms of prevention of cognitive impairment in AF patients, there is evidence that early and effective use of oral anticoagulation in patients with stroke risk factors reduces the rate of cognitive decline and currently, this represents the most important preventive strategy. Consequently, the main risk assessment for cognitive impairment in AF patients is the assessment of stroke risk factors, preferably by use of the CHA_2_DS_2_-VASc risk scheme that can guide the decision on oral anticoagulation. When cognitive impairment is suspected, brief screening tools such as General Practitioner Assessment of Cognition (GPCOG), Mini Mental State Examination (MMSE) and Montreal Cognitive Assessment (MOCA), and Informant Questionnaire on Cognitive Decline in the Elderly (IQCODE) may be applicable.^258^ In addition, more comprehensive assessments may be done after appropriate referral to a psychiatrist, geriatrician, or neurologist.^258^

### Risk assessment for stroke/transient ischaemic attack status post-left atrial appendage occlusion/ligation

Left atrial appendage (LAA) occlusion/ligation using one of several devices or surgical techniques has been developed as an alternative to anticoagulation in high-risk patients with non-valvular AF.^279–281^ The maximum experience has been with the Watchman device (Boston Scientific), which has been found to be non-inferior to warfarin in patients who are still candidates for short-term warfarin treatment.^282–284^ Results of comparison between LAA occlusion/ligation and NOACs are awaited. Current guidelines recommend use of LAA occlusion as a possible strategy in patients having contraindications to long-term anticoagulation.^261^

**Table:** 

Risk assessment for stroke/TIA after LAA occlusion/ligation	Class	References
TOE after 6 weeks and if necessary after 1 year is useful for detecting peri-device residual flow, incomplete appendage ligation, or device-related thrombus to identify patients at higher risk of stroke.		^271^ ^,^ ^272^
Clinical features such as previous TIA/stroke, persistent AF, low LVEF, vascular disease, and early discontinuation of anticoagulation may be helpful to guide decisions regarding imaging for device related thrombus.		^273^ ^,^ ^274^
Multi-detector CT and cardiac CT angiography have been found to be equivalent to TOE to detect peri-device flow.		^275^ ^,^ ^276^
After surgical occlusion or exclusion of the left atrial appendage, imaging may be useful to look for a residual appendage and its function or a residual leak after ligation to guide decisions regarding anticoagulation.		^277^ ^,^ ^278^ ^,^ ^535^

The residual risk of stroke/TIA following LAA occlusion/ligation can be related to procedural or patient related risk factors. Among the procedure related factors, peri-device leak, and device-related thrombus are important factors for thrombo-embolic events in short and medium term after the procedure. Stroke risk is significantly elevated in patients in whom LAA ligation fails after surgical^285^ or percutaneous approaches.^286^

Post-procedure surveillance is therefore important to assess long-term risk of stroke and need for continued anticoagulation. These may be detected on TOE immediately or after few weeks/months.^271^^,^^272^ Multidetector CT and cardiac CT angiography have been compared with TOE and found to be an effective alternative technique to detect peri-device flow.^275^^,^^276^ Device-related thrombus is seen in 3–7% of patients after LAA closure, and leads to a 3–4 fold higher risk of stroke.^273^^,^^274^ Factors predicting device-related thrombus are previous TIA/stroke, persistent AF, low LVEF, vascular disease, and early discontinuation of anticoagulation.^273^^,^^274^

If surgical LAA ligation fails or is incomplete, stroke rates are significantly increased. Similarly, with percutaneous closure devices, residual LAA leaks were associated with increased risk of thromboembolism in excess of that associated with baseline risk factors or echocardiogram findings.^285^

### Risk for heart failure incidence and progression

**Table:** 

Risk for heart failure incidence and prognosis	Class	References
Screening for AF in patients with HF should be performed because of the increased risk of adverse cardiovascular outcomes in combination more than the risk conveyed by either disease state alone.		^287^ ^,^ ^288^
Interval use of echocardiography and arrhythmia directed monitoring for development of AF-induced cardiomyopathy and risk assessment over time should be part of standard follow-up for patients with AF.		^289^ ^,^ ^290^

Atrial fibrillation and HF are conditions that coexist in many patients, and sometimes it will be difficult to establish if HF was the cause of AF or AF caused HF (tachycardia-induced cardiomyopathy).^287^^,^^291^ In the Framingham study, 41% of patients with AF and HF developed HF first, 38% developed AF first, and in the remaining 21%, AF and HF occurred at the same time.^288^ AF is associated with a three-fold increased risk of incident HF.^292^ In trials of patients with chronic systolic heart failure, the prevalence of AF was 4% in patients with Class I symptoms, 10–27% in patients with Class II–III symptoms, and 50% for those with Class IV HF symptoms.^290^ Additionally, aging and the structural and neurohormonal changes in HF make the development and progression of AF much more likely. The risks of developing an AF-induced cardiomyopathy appear to be related to the ventricular rate during AF and the duration of AF. However, the precise incidence of tachycardia-induced cardiomyopathy with AF, in patients with and without SHD is unknown.

The mechanisms and pathophysiology of AF and HF share several risk factors and common pathophysiologic processes. Hypertension, smoking, obesity, diabetes, renal impairment, sleep apnoea, and CAD are all associated with an increased risk of developing both HF and AF, and each condition increases morbidity and mortality when associated with the other. All types of HF (HFpEF or HFrEF) are associated with an increase prevalence of AF.^293^^,^^294^ There are no studies examining the role of monitoring to detect AF in asymptomatic patients with HF or the management of AF if detected. For patients with cardiac implantable electronic devices, remote monitoring is a tool for determining AF burden and is part of routine device follow-up. In patients with HF, the risk of AF is increased by several mechanisms, remodelling of atrial structure and increased fibrosis, ectopy promoted by atrial stretch, increased spontaneous firing in the pulmonary veins and alterations in calcium current handling in the atrial muscle and sarcoplasmic reticulum calcium content.^289^

The loss of atrial systole in AF impairs LV filling and can result in left ventricular dilatation, decrease in myocardial blood flow and increase in LV wall stress and end-diastolic pressure. Atrial fibrillation can decrease cardiac output by 25% particularly in patients with diastolic dysfunction. The mechanisms for reduction in cardiac output include loss of atrial contribution to ventricular filling, increased mitral regurgitation and decreased left ventricular filling time. The irregular and rapid ventricular contraction in AF can lead to LV dysfunction in an unknown percentage of patients and in some patients a tachycardia-induced cardiomyopathy results.^290^ The irregular ventricular response also compromises ventricular performance through changes in calcium handling and reduced expression of Serca and phospholamban phosphorylation. Management can vary widely according to presentation and should be individualized since treatments shown to be effective in one or other condition alone, may give rise to safety or efficacy issues in an individual patient. Several recent trials have suggested a preferential role for primary catheter ablation of AF in select AF patients with HF compared to medical therapy alone.^295–297^ Treatment of AF by either rate or rhythm control may reverse the cardiomyopathy and improve clinical HF substantially in selected patients.

### Risk for death in atrial fibrillation patients

**Table:** 

Risk for death in AF patients (including risk for SCD)	Class	References
Clinical characteristics of the patient including presence of advanced age, cognitive dysfunction or dementia, diabetes mellitus, hypertension, prior stroke, vascular. disease, and HF should be used as important risk markers of higher mortality in patients with AF.		^298^ ^,^ ^299^

**Figure 5:**
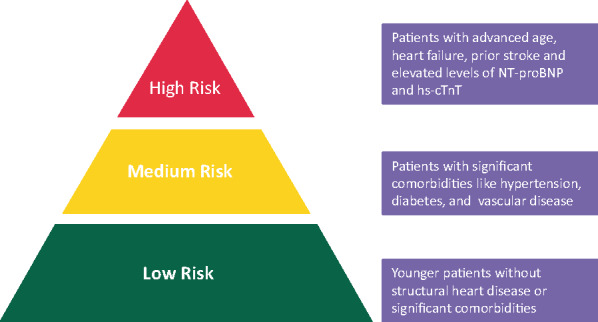
Mortality risk in patients with atrial fibrillation.

Atrial fibrillation is associated with 1.5- to 2-fold higher risk of all-cause mortality which may result from stroke, HF, or SCD.^261^ Of the mortality associated with AF, only 1 in 10 deaths are stroke, and >7 out of 10 are cardiovascular.^300^ A multipronged strategy incorporating stroke prevention, better symptom control, and cardiovascular risk optimization is associated with improved outcomes, including a reduction in mortality.^3^^,^^4^ Females with AF have slightly higher mortality compared to male patients. Ethnic or racial differences exist in mortality risk, with one study showing highest risk in African Americans among all racial/ethnic groups.^301^ Also, presence of comorbidities increases the risk compared with ‘lone’ AF. Advanced age, renal failure, pulmonary disease, and HF have been found to be most important risk factors for higher mortality in AF (*Figure 5*).^298^^,^^299^

Numerous risk scores have been designed to assess the mortality risk in AF. The CHA_2_DS_2_-VASc score was designed to assess stroke risk, but given it is a cluster of common risk factors for cardiovascular mortality also predicts mortality risk.^302^ More complex clinical risk scores designed to predict mortality, such as an integrated GARFIELD-AF risk tool, statistically improves mortality prediction, being superior to the CHA_2_DS_2_-VASc score.^303^ All clinical risk scores only have modest predictive value (c-indexes 0.6–0.7) but can always be statistically improved by the inclusion of cardiac biomarkers, such as NT-proBNP and hs-TnT. Both biomarkers (and others) have been found to be independently associated with increased midterm mortality in AF patients presenting to emergency room.^304^ Indeed, risk scores incorporating biomarkers have been proposed, such as the ABC-death risk score, which utilizes age, biomarkers, and clinical history. The ABC-death score achieved a c-index of 0.74 [95% confidence interval (CI) 0.72–0.76], while the CHA_2_DS_2_-VASc score achieved a c-index of 0.58 (95% CI 0.56–0.61).^305^ However, the clinical usefulness of any risk-prediction score for mortality has not been established, and further validation studies are needed. Indeed, many risk factors or biomarkers are based on measurements done at baseline, and follow-up events occur many years later. Cardiovascular risk is not static but changing with increasing age and incident risk factor(s), thus repeat risk re-assessment is more appropriate given that a change in risk scores is more highly predictive of adverse outcomes.

Importantly, many biomarkers are non-specific, more likely reflecting a patient with significant comorbidities and significant underlying heart disease, and are predictive of various endpoints apart from death, including stroke, heart failure, etc.^306^^,^^307^ Indeed, biomarker-based scores like ABC-death were derived from a highly selected clinical trial cohort which was anticoagulated, and values were determined at study entry (baseline). Many biomarkers also have a diurnal variation and inter/intra laboratory variability and are predictive of non-cardiovascular outcomes. Real-world studies investigating the usefulness of sequential addition of biomarkers have shown limited value over conventional clinical risk scores.^10^^,^^308^^,^^309^ Thus, statistically significant improved prediction should not be confused with clinically improved risk prediction. A balance is therefore needed between (statistically) improved risk prediction and simplicity or practicality for everyday clinical use in busy clinical settings. In summary, any novel biomarker (or biomarker-based scores) would need to be validated in large non-anticoagulated cohorts. This is the starting point of risk stratification with the newly diagnosed AF patient in any patient care pathway, and be simple, practical and adequately validated to account for the dynamic nature of risk factors and changes in drug therapies (including the use of antithrombotic drugs) over time.

Stroke resulting from AF has significant medium-term mortality, which can be as high as 30.5% at 1 year.^310^ An 8-point GPS-GF score utilizing variables including Glasgow Coma Scale, pneumonia, midline shift on brain images, blood glucose, and female sex has been developed and was found useful to predict 30-day mortality in patients with AF-related stroke.^311^

Spontaneous AF is associated with an increased risk of SCD in patients with Wolff–Parkinson–White (WPW) syndrome, HCM, and channelopathies such as Brugada syndrome.^261^ Several recent studies on HF and LVH and those on the general population have reported that AF is linked to an increased risk of SCD.^312–314^ Mechanisms for SCD due to AF are well understood for WPW syndrome or HCM, but are unclear regarding other cardiac disorders. A meta-analysis demonstrated a significant association between AF and SCD in the general population as well as in patients with CAD, congestive HF, HCM, Brugada syndrome, and implanted rhythm devices.^315^ In a nationwide cohort study from Taiwan, 352 656 patients were identified. Among AF patients, age ≥75 years, congestive heart failure, hypertension, diabetes mellitus, previous stroke/TIA, vascular diseases, chronic kidney disease, and chronic obstructive pulmonary disease were important risk factors for SCD or ventricular arrhythmias.^316^ A recent study suggested that optimal pharmacological treatment, in addition to anticoagulant therapy, can reduce SCD rates in patients with AF.^317^ Since pharmacological rhythm control has so far been relatively ineffective in preventing SCD in AF patients with low LVEF,^318^ catheter ablation may be more appropriate for improving prognosis in patients with AF.^296^ To assess the risk of SCD in patients with AF, recognizing the presence of CAD, HF, LVH/HCM, pre-excitation, Brugada syndrome, and implanted rhythm devices is crucial. Examinations including 12-lead ECG, echocardiography, and other imaging modalities such as cardiac MRI are useful for detecting various cardiac disorders. Electrophysiological testing is useful for identifying risks in patients with WPW syndrome and paroxysmal AF.

### Risk of adverse outcomes in patients treated with catheter ablation

Radiofrequency (RF) ablation has emerged as a main therapeutic option for treatment of AF patients since 1998 after the observation that AF mostly initiates from arrhythmogenic triggers in muscular sleeves in the pulmonary veins.^326^ There is abundant evidence that AF ablation is an effective method for AF suppression leading to significant reduction of AF episodes and burden accompanied by substantial improvement in symptoms and quality of life if performed in symptomatic patients. For this reason, AF ablation is mainly recommended by current guidelines as a method for symptom improvement in symptomatic AF patients.^261^

**Table:** 

Risk of adverse outcomes in patients treated with catheter ablation	Class	References
Patients that undergo an AF ablation should be monitored closely in the first 30 days after the procedure due to a higher risk of neurological, gastrointestinal, cardiovascular, vascular and peripheral complications.		^319–324^
Wolff–Parkinson–White syndrome patients following radiofrequency ablation may benefit from additional follow-up due to a persistent elevated risk of developing AF compared to the general population.		^295^ ^,^ ^325^

#### Post-ablation atrial fibrillation recurrence

Post-ablation AF recurrence is one of the most important and frequent adverse outcomes, which occurs in 30–50% of cases.^327^^,^^328^ In fact, although the acute success rate of AF catheter ablation seems high, achieving a durable treatment efficacy has remained a main challenge.^261,^^328^ Different factors including female gender, older age, traditional cardiac risk factors, left ventricular dysfunction, increased epicardial adipose tissue, myocardial fibrosis, and atrial enlargement have been proposed as possible predictors of post-ablation AF recurrence.^329–331^ Moreover, diverse AF recurrence risk-prediction scores, including APPLE, ALARMEc, ATLAS, BASE-AF2, CAAP-AF, DR-FLASH, and MB-LATER have been introduced; however, their integration into the daily clinical practice needs further support by healthcare systems.^332–341^

#### Other adverse outcomes

Apart from AF recurrence, according to the available real-world data, around 5–15% of patients undergoing AF catheter ablation experience complications, mainly during the index hospitalization and early in the post-procedure course.^319–324^ A variety of complications, including neurological, gastrointestinal, cardiovascular, vascular and peripheral, as well as pulmonary complications have been reported to occur after ablation procedures.^319–324^^,^^342–346^ Although different modifiable factors such as metabolic syndrome, hypertension, alcohol consumption, sleep apnoea, and obesity have been proposed to be related with arrhythmia-free survival after catheter ablation,^347–350^ their impact on the ablation adverse outcomes is not clear yet, and requires further investigations.

##### Mortality and morbidity

The impact of the ablation on hard clinical endpoints is much less evident. Previous findings from observational studies indicated a positive effect of the procedure on mortality and morbidity.^351^ These, however, were not confirmed in the recent large randomized Catheter Ablation vs Antiarrhythmic Drug Therapy for Atrial Fibrillation Trial (CABANA) that had as primary endpoint a composite of death, disabling stroke, serious bleeding, or cardiac arrest.^295^ In contrast, positive effects on hard clinical endpoints including mortality have been reported in patients with HF. In the CASTLE-AF trial, patients with impaired LVEF <35% and previous ICD implantation who were treated with ablation therapy had a lower rate of death from any cause or hospitalization for worsening HF compared to patients undergoing medical treatment.^296^

##### Stroke

Regarding the impact of AF ablation on stroke and in particular the validity of stroke risk schemes for stroke risk stratification after ablation, observational data suggest a reduced stroke risk after AF ablation and a possibly safe termination of anticoagulation, at least in selected patients.^352^^,^^353^ Conclusive evidence is expected from ongoing randomized trials as the Optimal Anticoagulation for Higher Risk Patients Post-Catheter Ablation for Atrial Fibrillation Trial (OCEAN) (NCT02168829) and the Prevention of Silent Cerebral Thromboembolism by Oral Anticoagulation with Dabigatran After Pulmonary Vein Isolation for Atrial Fibrillation (ODIn-AF) trial (NCT02067182). Until now, one randomized trial showed that ablation therapy for AF in patients with impaired LVEF was associated with significantly lower rate of death from any cause and worsening HF.^274^ Subgroup recommendations may change after the completion of trials studying the effect of ablation on stroke and the need for anticoagulation. Particularly in HF patients, it remains to be seen in which subgroups of patients the data indicating mortality reduction after AF ablation are applicable.

#### Catheter ablation in Wolff–Parkinson–White patients

Careful attention must be given in WPW patients who underwent RF ablation, as it was demonstrated that they had an increased risk of AF at follow-up when compared to general population, though an increased risk of death was not reported.^296^^,^^325^

### Risk of adverse outcomes in patients treated with surgical Maze

The surgical Cox–Maze operation was introduced in 1987 to treat patients with refractory AF.^354^ This surgical approach carries more risk of complications than the catheter ablation procedure, and is suitable for selected patients only. In this setting, we can observe three different case-scenarios.

#### Atrial fibrillation surgery

A simplification of the Cox–Maze procedure was proposed by replacing the ‘cut and sew’ lesions by different ablation devices and minimally invasive access.^355^ In the recent years, bipolar RF clamping devices guided on a beating heart, by thoracoscopic epicardial approaches have been introduced.^277^^,^^356^ This evolution has allowed the implementation of this surgery for stand-alone persistent and long-standing persistent AF ablation, after an ineffective antiarrhythmic drug treatment or a previous endocardial ablation failure with a IIa (Level of Evidence B) indication.^278^ On another hand, this invasive approach carries some potential risks that need to be anticipated and discussed. Ideally, this step should involve an arrhythmia team in order to discuss the risk–benefit balance of the procedure on a case by case basis.^357^

#### Surgical Maze in patients with concomitant heart surgery

An AF surgical ablation procedure is reasonable for selected patients with AF undergoing cardiac surgery for other indications.^261^ In patients that may receive a concomitant Maze procedure, a shared decision-making strategy should be used with an AF heart team to make the best decision available for the patient and their heart condition.^357^ Mortality or major morbidity was not affected by concomitant AF surgery [adjusted odds ratio (OR) 1.00; 95% CI 0.83–1.20], but pacemaker implantation was more frequent (adjusted OR 1.26; 95% CI 1.07–1.49).^358^ Stiff LA syndrome was also reported after surgical Maze procedure, presenting with dyspnoea, pulmonary hypertension, and elevated left ventricular end-diastolic pressure attributed to reduced LA compliance.^359^

Predictors of AF recurrence after surgery include left atrial dilatation, older age, over 10-year history of AF, and non-paroxysmal AF.^360–364^

#### Stand-alone surgical Maze

A stand-alone AF surgical ablation procedure may be reasonable for selected patients with highly symptomatic AF not well managed with other approaches (e.g. after a failed catheter ablation, longstanding AF, dilated left atrium).^365^ After Cox–Maze IV stand-alone procedure, overall operatory mortality was 1–1.8%, overall complication rate was 10%, 8% required pacemaker placement, and 12-month freedom from atrial tachyarrhythmias was 89% (78% without antiarrhythmic drugs).^366^^,^^367^

#### Left atrial appendage exclusion or removal during surgical Maze

The prospective randomized trial comparing the efficacy and safety of LAA exclusion or removal with surgical Maze procedure is lacking. However, epicardial LAA Atriclip occlusion showed a high rate of complete left atrial appendage occlusion and reduces the incidence of stroke in patients with AF undergoing cardiac surgery.^281^ After surgical occlusion or exclusion of the LAA, it is recommended to continue anticoagulation in at-risk patients with AF for stroke prevention.^261^ If surgical LAA ligation fails or is incomplete, stroke rates are significantly increased compared to patients with complete closure.^285^

## How to assess risk for ventricular tachyarrhythmia in specific populations

### Patients with ischaemic heart disease

Ventricular tachyarrhythmia/ventricular fibrillation events are closely related to the risk of SCD in patients with ICM. For this reason, the risk of VT/VF is commonly used as a surrogate for the risk of SCD. In addition, in ICM, myocardial ischaemia is the most common trigger for VF and SCD.

For primary prevention, our current approach to SCD risk stratification relies mainly on the evaluation of LVEF: values below 30–35% allow the identification of ICD candidates, who are at highest relative risk of SCD. On the other hand, patients with a LVEF >35% account for the highest absolute number of SCDs.^368^ For this reason, many researchers emphasize that EF is an inadequate marker for detecting patients who are at high risk for SCD despite having a normal or sub-normal EF. It seems also to have very limited value to identify amongst patients with a low LVEF those who will benefit the most from an ICD. In other words, many patients with EF ≤35% are unnecessarily implanted with an ICD for primary prevention, while some others, having a EF >35% and a high risk of VT/VF, are not protected. In this setting, new markers are needed to optimize screening and patient selection for ICD implantation. For secondary prevention, SCD risk is significantly higher, and risk stratification is certainly more standardized.^61^^,^^74^

#### Secondary prevention of ventricular tachyarrhythmia/ventricular fibrillation in patients with ICM

**Table:** 

Secondary prevention of VT/VF in patients with ICM	Class	References
ICM substrate and ischaemic triggers for VT/VF must be evaluated when appropriate (coronary angiogram, functional ischaemic evaluation by nuclear scan, stress-echocardiography, or MRI).		^54^ ^,^ ^70^ ^,^ ^71^
Cardiac MRI with a LGE can be considered in order to evaluate arrhythmogenic substrate including myocardial scarring to include in risk assessment, and guide a possible VT ablation procedure. This investigation should be preferably performed before ICD implantation to avoid artefacts due to the presence of an implanted device.		^369^

For more than 20 years, patients with a history of sustained VT/VF have been recognized to be at high risk of recurrence.^370^ Nowadays, these patients are given a Class I (Level of Evidence A) indication for ICD implantation.^70^ For this reason, the practical usefulness of VT/VF recurrence risk assessment is questionable, as additional testing is likely not going to influence decision pathways (i.e. catheter ablation or antiarrhythmic drug therapy as an alternative to ICD implantation), and patient outcomes in a secondary prevention setting.

#### Primary prevention of ventricular tachyarrhythmia/ventricular fibrillation in patients with ICM and a left ventricular ejection fraction ≤35%

Patients presenting with ICM, in NYHA Class II–III, with EF ≤35% after 3 months of optimized heart failure pharmacological treatment, are given a Class I/A indication for ICD implantation for the primary prevention of SCD.^70^ Nonetheless, it is widely recognized that only a small subgroup of these patients will present with VT/VF during follow-up, and consequently will benefit from the device. A better risk stratification of these patients would be crucial to help identify those who would indeed benefit from an ICD. Most of the numerous investigations assessed in this setting, like programmed ventricular stimulation (PVS), heart rate variability (HRV), late ventricular potentials (LVP), baroreflex sensitivity, QT interval dispersion, T-wave alternans, and heart rate turbulence have been largely abandoned because none of them have influenced routine clinical practice.^46^^,^^73^^,^^371^^,^^372^ However, some of these explorations, like T-wave alternans, have shown some value for SCD prediction in ICM patients.^42^ It is still uncertain whether biochemical markers as B-type natriuretic peptide and N-terminal pro-BNP will prove useful in assessing risk for VT/VF. Cardiac MRI with LGE should also help to improve VT/VF and SCD risk stratification by analysing cardiac structure and myocardial scarring.^375^ Finally, a recent randomized trial suggests that assessment for hibernating myocardium performed routinely is of no use to decrease the risk of SCD.^68^

**Table:** 

Primary prevention of VT/VF in patients with ICM and LVEF ≤ 35%	Class	References
ICM substrate and ischaemic triggers for VT/VF must be evaluated when appropriate (coronary angiogram, functional ischaemic evaluation by nuclear scan, stress-echocardiography or MRI).		^54^ ^,^ ^70^ ^,^ ^71^
Cardiac MRI with a LGE can be considered in order to evaluate arrhythmogenic substrate including myocardial scarring to include in risk assessment and guide a possible VT ablation procedure. This investigation should be preferably performed before ICD implantation to avoid artefacts due to the presence of an implanted device.		^369^

#### Primary prevention of ventricular tachyarrhythmia/ventricular fibrillation in patients with ICM and left ventricular ejection fraction > 35%

This group of patients should be the priority for VT/VF risk assessment: in absolute numbers, it represents by far the highest number of those at risk of VT/VF and SCD.^368^ In addition, these patients are currently non-protected, as they are not targeted for ICD implantation in guidelines, due to their LVEF value.^70^ In this setting, MRI with LGE could be an option to better understand the diagnosis, prediction, and treatment of VT/VF.^369^ This investigation could possibly help improve VT/VF and SCD risk stratification by analysing cardiac structure and myocardial scarring, particularly when EF is relatively preserved. In this setting, a large prospective trial documenting that treatment guided by MRI-based risk stratification improves outcomes in this patient group is still very much expected.^375^

**Table:** 

Primary prevention of VT/VF in patients with ICM and LVEF > 35%	Class	References
ICM substrate and ischaemic triggers for VT/VF must be evaluated when appropriate (coronary angiogram, functional ischaemic evaluation by nuclear scan, stress-echocardiography or MRI).		^54^ ^,^ ^70^ ^,^ ^71^
EPS and non-sustained VT evaluation could be considered to improve VT/VF risk stratification in patients with relatively preserved LVEF, particularly in the convalescent phase (first 2 months) after an acute coronary syndrome.		^311^ ^,^ ^373^ ^,^ ^374^
Heart rate variability (HRV), LVP, baroreflex sensitivity, QT-interval dispersion, T-wave alternans and heart rate turbulence have not been evaluated adequately in this population for generalized use.	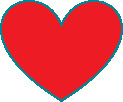	^73^ ^,^ ^371^ ^,^ ^372^

Otherwise, the MUSTT Trial suggested the value of EPS for improving the SCD risk stratification, in the subgroup of ICM patients with a residual EF comprised between 30 and 40%.^376^

In addition, other non-invasive investigations like tissue Doppler Imaging (TDI) seem also to be of potential value in predicting VT/VF in ICM. Late diastolic velocity assessed by TDI, particularly when detected in the inferior myocardial wall, seems to be a sensitive marker of future VT/VF.^373^ Finally, it is well known that non-sustained ventricular tachycardia (NSVT) is a marker of increased risk of VT/VF and arrhythmic death. During the convalescent phase after an acute coronary syndrome, NSVT seems to be associated with an increased risk of cardiovascular death, most marked within the first 2 months after detection.^374^ The use of such investigations could help to detect those patients at higher risk of VT/VF, more particularly during the early phase after an acute coronary event. Specific measures like prolonged monitoring or use of wearable cardiac defibrillator could be undertaken on an individual patient-case basis. However, more solid data are needed to support such recommendations broadly.

### Patients with non-ischaemic heart failure

**Table:** 

Patients with non-ischaemic heart failure	Class	References
MRI may be considered for further risk stratification of sudden death in patients with non-ICM who do not otherwise meet an indication for ICD implantation.		^377^
EPS may be considered for further risk stratification of sudden death in selected patients with non-ICM who do not otherwise meet an indication for ICD implantation.		^377^

Patients with non-ischaemic HF represent a broad and diverse group of patients including those with progressive and infiltrative forms of cardiomyopathies. For this reason, the risk of developing VT in non-ischaemic HF is difficult to accurately predict in this group of patients. Subsequent sections in this document will address specific conditions that have unique risk profiles including inflammatory cardiomyopathies, congenital heart disease, ACM, and Chagas’ disease.

Prior investigations into identification of the risk of developing VT in non-ICM focused on the risk of SCD and the role of the implanted defibrillator for primary prevention. The DANISH trial^61^ reported no survival benefit from prophylactic ICD implantation in the overall cohort. Implantable cardioverter-defibrillator reduced SCD to half, and subgroup analysis showed that in patients younger than 68 years, survival was prolonged with an ICD. Although pooled analysis of the five primary prevention trials (DEFINITE, SCD-HeFT, CAT, AMIOVIRT, COMPANION, and DANISH; *n* = 2970) revealed that ICD therapy was superior to medical therapy in patients with non-ICM with decreased cardiac function, these trials were judged globally negative.^378^

In a limited number of studies outside of these clinical trials, the role of EPS or non-invasive programmed stimulation has revealed inconsistent results.^377^ More recently, the role of cardiac MRI for definition of scar and potential substrate has emerged as a powerful risk stratification tool in observational studies.^49^^,^^379^^,^^380^ Genetic testing is also useful in patients with decreased cardiac function with conduction disturbance (i.e. LMNA mutations).

In summary, non-ischaemic HF includes a diverse group of patients with reduced ventricular function due to cardiomyopathies from different aetiologies, and at high risk for VT. Reduced cardiac function remains a powerful predictor of VT and appropriate ICD therapy in these patients as a primary prevention. Cardiac MRI and EP testing shows promise in some subsets. Further characterization based on the type of cardiomyopathy leading to HF shows the most promise for accurate assessment of VT risk.

### Patients with inflammatory cardiomyopathies

**Table:** 

Patients with inflammatory cardiomyopathies	Class	References
In patients with non-ischaemic heart disease who present with ventricular arrhythmias, use of cardiac MRI or cardiac PET can help delineate aetiology of non-ICM, initiate aetiology-driven treatment, and evaluate prognosis.		^52^ ^,^ ^53^ ^,^ ^379^

Inflammatory cardiomyopathies encompass a broad spectrum of disorders characterized by myocardial inflammation as the primary cause of cardiac dysfunction. This includes viral myocarditis (commonest cause), cardiac sarcoidosis, giant cell myocarditis, autoimmune myocarditis associated with underlying connective tissue diseases, eosinophilic cardiomyopathies, and Chagas disease (addressed in a separate chapter).

In patients who present with ventricular arrhythmias and diagnosed with non-ICM, the incidence of inflammatory cardiomyopathy may be as high as 50%.^381^ Therefore, it is important to consider inflammatory cardiomyopathies as an underlying cause, given that these conditions may benefit from specific aetiology-driven treatments. Infectious causes of myocarditis include viral (e.g. parvovirus B19 and human herpes virus 6 genomes that predominate in inflammatory cardiomyopathies, other cardiotropic viruses include enteroviruses, adenoviruses, hepatitis C, and human immunodeficiency viruses) and uncommonly bacterial and other causes depending on the geographical area and immunosuppression status. Myocarditis associated with connective tissue and autoimmune diseases encompass systemic lupus erythematosus, scleroderma, rheumatoid arthritis, dermatomyositis, polymyositis, cardiac sarcoidosis and giant cell myocarditis. Drug reactions may also cause hypersensitivity myocarditis.^381^^,^^382^ In cases of an established cause of inflammatory cardiomyopathy, the focus should be on treating the underlying inflammatory condition. In the case of cardiac sarcoidosis, retrospective series have shown that specific treatment with immunosuppressive therapy can increase VT free survival.^52^

Cardiac MRI scan is the gold standard for diagnosing myocarditis and inflammatory cardiomyopathies. Oedema, hyperaemia, and LGE form the diagnosis of acute myocarditis. Further diagnostic information is gleaned from T1 and T2 mapping techniques. Although no specific LGE pattern on MRI is diagnostic of cardiac sarcoidosis, LGE is most often observed in basal segments, particularly of the septum and lateral wall, and usually in the mid-myocardium and epicardium of the myocardium^383–385^

The presence of LGE is significantly associated with increased risk of adverse cardiac events. The presence of LGE on cardiac MRI was associated with increased risk of ventricular arrhythmias and death by greater than 20 fold in patients with EF >35% and extracardiac sarcoidosis compared to sarcoid patients without evidence of LGE on MRI, and the burden of LGE was associated with higher rates of death/VT.^386^ In a meta-analysis of 155 patients with systemic sarcoidosis who underwent cardiac MRI for work-up of cardiac sarcoidosis, the presence of LGE was associated with hazard ratio of 31.6 for death, aborted SCD, or appropriate ICD discharge and provided superior prognostic information as to compared to other clinical and functional characteristics, including LVEF.^51^

In addition, the distribution of LGE confers important prognostic information, with mid-wall anteroseptal LGE representing a more malignant form compared to a sub-epicardial inferolateral wall LGE pattern.^387^^,^^388^ Inflammatory biomarkers, such as C-reactive protein, are typically lower in this group with septal LGE, but biomarkers of myocardial damage such as troponin are typically higher, suggestive of a subset with less inflammation but greater myocardial injury. F-fluorodeoxyglucose (FDG)-PET is advantageous for detecting active inflammation in cardiac sarcoidosis, and a mismatch of FDG and perfusion and involvement of the right ventricle predicts adverse cardiac events and ventricular arrhythmias, respectively.^53^ Endomyocardial biopsy is performed in cases where a histological diagnosis is required to confirm cardiac sarcoidosis or giant cell myocarditis, with its yield enhanced by electrogram guidance. Active viral genomes may also be identified by biopsy, which can differ significantly from peripheral serological tests.^382^^,^^389^

Little data exist on how to assess risk of VT/VF in inflammatory cardiomyopathies. Besides EF, which is used for all non-ischaemic aetiologies, no randomized studies have evaluated other parameters or even EF as a predictor of VT in different inflammatory cardiomyopathies. In particular, certain inflammatory cardiomyopathies may carry higher risk than others (sarcoidosis vs. viral myocarditis). Risk of ICD therapy may be as high as 15% per year in biopsy proven cardiac sarcoidosis patients.^390^ Although randomized data on use of higher EF in these patient populations is lacking, given risk of VT noted in retrospective studies, use of MRI and cardiac PET to evaluate aetiology of non-ischaemic heart disease is warranted, and treatment of inflammation to reduce risk of VT is advised. Furthermore, cardiac PET and MRI can be used to assess for recurrent inflammation or progression of disease on treatment.

### Patients with congenital heart disease

Ventricular arrhythmias in patients with congenital heart disease (CHD) may be observed in two different groups: the paediatric age group and adults with repaired congenital defects group.^397^ In the paediatric age, life-threatening VT is rare both prior to and after surgery. Ventricular tachyarrhythmia is seen in only 1.8% of children undergoing an EPS,^391^ is usually associated with structurally normal heart and most frequently comes from the right outflow tract and left outflow tract and sinuses of Valsalva.

**Table:** 

Risk for ventricular arrhythmias in patients with congenital heart disease	Class	References
In the paediatric patient with CHD, ventricular overload, surgical scars and patches or baffles, ventricular dysfunction, and previous conduction defects are recognized risk factors for VT.		^391–393^
In adult patients with CHD, older age at surgery, poor haemodynamic status, and prolonged QRS represent the most common risk factors for ventricular arrhythmias.		^392–394^
In adult patients with CHD, VTs are mainly observed after correction of tetralogy of Fallot (TOF) and left ventricular outflow tract defects.		^394–396^
In patients with TOF, residual haemodynamic lesions and ventricular dysfunction represent the most important risk factors for VT or SCD.		^394–396^
In patients with TOF, frequent PVCs, QRS >180 ms, palliative systemic to pulmonary shunts, syncope, atrial tachycardia, decreased LVEF, dilated right ventricle, severe pulmonary stenosis or regurgitation, are risk factors for sustained VT.		^394–396^

In paediatric patients with CHD, the haemodynamic and electrophysiologic factors related to each disease state and associated therapeutic interventions play an important role in the development of VT, with ventricular overload, surgical scars and patches, baffles and conducts, ventricular dysfunction, and previous conduction defects among the most relevant.^392^ In the early post-operative stage, Van Hare *et al*. reported only 3 patients with VT out of 580 undergoing paediatric surgery and the most important risk factor was the surgical procedure.^391^ Sustained VT may arise in the setting of myocardial ischaemia or infarction and may be facilitated by disruption of the ventricular myocardium caused by scar due to ventriculotomy, fibrotic tissue, or ventricular dilatation.^393^

In adult patients with CHD, VTs are mainly observed after correction of tetralogy of Fallot (TOF) and left ventricular outflow tract defects but may also arise in other defects as transposition of the great arteries with atrial switch, univentricular hearts, double-outlet RV, and ventricular septal defects. Older age at surgery, poor haemodynamic status, and prolongation of the QRS represent the most common risk factors. In patients with TOF, the correlation of residual haemodynamic lesions and right ventricular dysfunction with risk of VT or SCD has been extensively established.^394^^,^^395^ Potentially treatable residual haemodynamic problems, pulmonary hypertension, elevated end-diastolic pressures, and reduced ventricular function should be treated as part of the arrhythmia management. Particularly in this group, frequent PVCs, QRS 180 ms or more, palliative systemic to pulmonary shunts, syncope, atrial tachycardia, decreased LVEF, dilated right ventricle, severe pulmonary stenosis or regurgitation are risk factors for sustained VT, and inducible sustained VT correlates with increased risk of SCD.^396^^,^^398^ EPS might be considered for risk assessment of VT/VF in this group of patients with high-risk clinical characteristics and frequent ventricular arrhythmias.^327^

### Patients with inherited arrhythmia diseases (inherited channelopathies and inherited structural diseases including arrhythmogenic right ventricular cardiomyopathy)

**Table:** 

Risk for ventricular arrhythmias in patients with inherited arrhythmia diseases	Class	References
Patients with primary inherited arrhythmia syndromes and cardiomyopathies should undergo risk stratification that integrates clinical presentation, family history, and non-invasive diagnostic testing.		^399^
Select patients with primary inherited arrhythmia syndromes and cardiomyopathies may benefit from electrophysiologic testing to refine non-invasive risk stratification.		^536^

Patients with inherited arrhythmia disease are without doubt at increased risk for ventricular arrhythmias, including SCD. The extent to which this is pertinent and predictable is different for the various conditions.

The main primary inherited arrhythmia syndromes, i.e. the ‘channelopathies’ are LQTS, Brugada syndrome and CPVT.^400^ Patients that are symptomatic (syncope, cardiac arrest) at the time of presentation are at highest risk, with arrhythmic syncope representing a sentinel sign of risk, and resuscitated cardiac arrest reflecting the highest risk cohort.^97^ Despite major social impact on perceived risk, family history is not of major importance in all three diseases.

In LQTS, clearly defined disease-specific risk factors are the extent of resting QT prolongation, documentation of arrhythmias and gene and even mutation specific associated risk.^401^ In CPVT, the extent of the arrhythmic response of an exercise test predicts events, including breakthrough symptoms on therapy.^402^ It follows that risk assessment requires a baseline ECG and an exercise test in both conditions, with potential value of ambulatory monitoring. Assessment should include asymptomatic patients often identified during family screening or after incidental unrelated medical evaluation.

In Brugada syndrome, there is uncontested agreement that symptomatic patients (arrhythmic syncope, cardiac arrest) are at high risk for SCD, requiring aggressive therapy with an ICD in most circumstances. Risk stratification in asymptomatic individuals with a spontaneous type 1 ECG is much less clear, involving a variety of ECG characteristics and potential value of programmed electrical stimulation (PES).^403^^,^^404^ ECG parameters that have been associated with increased risk include QRS fragmentation, early repolarization, Brugada type changes in non-anterior precordial leads and a positive signal-averaged ECG. Programmed electrical stimulation with a non-aggressive stimulation protocol may be of importance, although the risk of an inducible patient is only marginally different from a non-inducible patient.^77^ In LQTS, CPVT, and Early Repolarization syndrome, PES is of no importance. The presence of a SCN5a mutation may contribute to risk in Brugada syndrome.^405^ Early repolarization syndrome, short-coupled idiopathic VF (SCIVF), and SQTS are uncommon causes of cardiac arrest and sudden death. Though the early repolarization pattern conveys a small increase in risk, the only patients where the risk is substantive to consider intervention are those with prior cardiac arrest or syncope with a positive family history. There are no validated risk models in SQTS and SCIVF.

In the cardiomyopathies, i.e. the secondary inherited arrhythmia syndromes, risk stratification is also disease specific. In hypertrophic cardiomyopathy (HCM) septal thickness, the hallmark of the disease is an important contributor to risk. Other risk factors include left atrial dimension, left ventricular outflow tract gradient (all echocardiographic parameters), the presence of ventricular arrhythmias on ambulatory monitoring (Holter) or documentation otherwise, symptoms (i.e. unexplained syncope, palpitations associated with near syncope), demographic factors (age in particular), and family history. All these factors are included in the ESC risk score calculator,^406^ which is readily available in an online tool (http://www.doc2do.com/hcm/webHCM.html), and applied after standard imaging, exercise testing and ambulatory monitoring. Validation of the ESC risk calculator is not compelling, and consideration of imaging and exercise blood pressure response parameters have also been used in borderline cases. In inherited, i.e. non-ischaemic, dilated cardiomyopathy (DCM), the genetic background is very important, with LMNA (Lamin A/C) and PLN (Phospolamban) leading to highly arrhythmic substrates.^123^^,^^407^^,^^408^ Of course, reduced LVEF and the presence of ventricular arrhythmias during ambulatory monitoring are important risk factors as well. In arrhythmogenic right ventricular cardiomyopathy (ARVC or ACM), symptomatic arrhythmic events identify the patient at highest risk, and major risk factors include age, male sex, unexplained syncope, non-sustained VT, number of anterior precordial leads with T wave inversion, and severe right or left ventricular dysfunction.^409^ Hence, as for the other cardiomyopathies, echocardiographic imaging, and Holter monitoring is required for risk assessment. In all cardiomyopathies, MRI is becoming increasingly important, in particular to show the presence of fibrosis (HCM, DCM, ACM) and assess left and right ventricular function. Genetic testing should be considered in any patient with a phenotype suggesting an inherited cardiomyopathy and in DCM with a suggestive family history or onset at an early age that is otherwise unexplained (i.e. not myocarditis, sarcoidosis etc.). Genetic testing is largely for diagnosis, and only informs risk when a high-risk form of cardiomyopathy is diagnosed, such as PLN or LMNA.

### Risk stratification in patients with ACM, specified for arrhythmogenic right ventricular cardiomyopathy

**Table:** 

Risk stratification of ventricular arrhythmias in ARVC	Class	References
In patients with ARVC, history of aborted sudden death, sustained ventricular arrhythmias, and severe right and/or left ventricular dysfunction identify a high risk of cardiac death.		^410^ ^,^ ^411^
In patients with ARVC, advice to not perform high-level or endurance exercise should be given.		^412^ ^,^ ^413^
Clinical factors including age, male sex, unexplained syncope, non-sustained VT, number of anterior precordial leads with T wave inversion, and genetic mutation status can be used for prognostic stratification of patients with ARVC.		^410^ ^,^ ^411^
In patients with confirmed ARVC, regular Holter monitoring and imaging for assessment of ventricular function may be useful.		^412^ ^,^ ^413^
A detailed history of exercise intensity and duration may be helpful in patients with ARVC as exercise level may represent a modified risk factor of adverse cardiovascular events and disease progression.		^414^

In arrhythmogenic right ventricular cardiomyopathy (ARVC or ACM), the most important features characterized as the high arrhythmic risk include the electric instability (i.e. sustained ventricular arrhythmia [VA]), genotype-positive, extent of structural involvement, cardiac syncope, the presence of multiple mutations, and the history of competitive or endurance exercise.^410^^,^^411^ In patients without prior VA, an available online prediction model, derived from the largest cohort of ARVC patients, using readily available clinical parameters was devised to estimate the risk of VA and to guide the decisions of ICD implantation as primary prevention (www.arvcrisk.com).^415^

There is a dose-dependent relationship between endurance exercise and the disease onset and progression in confirmed ARVC patients. Exercise restriction is recommended to prevent disease progression and SCD in confirmed ARVC patients with ICD^412^ and genotype-positive relatives.^413^ In general, high-level or endurance exercise is not recommended in confirmed ARVC patients or at risk.

Ambulatory ECG monitoring is crucial to detect the PVCs burden or the presence of non-sustained VT, which also provide prognostic information in ARVC.^414^ All positive criteria of signal-averaged ECG non-invasively identifies the slow conduction of myocardium and has been proven for risk stratification in patients with suspicion or confirmed ARVC.^416^

Echocardiography and cardiac MRI provide accurate measurements of right ventricular global and regional dysfunction and right ventricular volume and regional/global ventricular function, as the important variable for assessment of right and left ventricular disease. The Task Force Criteria did not include cardiac MRI measures of right ventricular myocardial fat or LGE in order to risk stratify the ARVC.^417^ In summary, abnormal cardiac MRI was an independent predictor of clinical events with a cumulative effect of the abnormalities including morphology, wall motion, and fat/fibrosis in ARVC patients.^416^

An EPS may provide help distinguish ARVC from idiopathic right ventricular outflow tract (RVOT) VT. Additionally, positive inducibility on program ventricular simulation is not a perfect surrogate marker neither for ARVC diagnosis, nor the decision of ICD implantation.^410^^,^^411^ EPS may be beneficial to identity patients that may benefit from ablation. In this setting, EPS with high-dose isoproterenol may help differentiate patients with idiopathic VT or ventricular premature beats from those with ARVC.^418^ The positive inducibility of EPS can predict any ICD therapy, including VF, and can be an important parameter for risk stratification in patients with ARVC.

ARVC is considered to have desmosome dysfunction. Genetic causes of isolated or predominantly RV arrhythmia and structural abnormalities are most commonly associated with desmosomal gene variants. Positive genetic test contributes up to 50% of the diagnosis of ARVC, however, in confirmed ARVC patients, limited evidence of clinical actionable risk stratification or use of management of disease. Several gene variants have been reported in patients with left ventricular or biventricular arrhythmia. Left ventricular dysfunction is most often present in patients with ARVC with pathogenic variants in Lamin A/C, or variants in the PLN and TMEM43 genes, and followed by variants in DSP, DSG2/DSC2.^123,^^399^^,^^419^^,^^420^

### Patients with Chagas’s disease

Chagas disease is an infectious disease affecting 10 million people around the world and 100 million more are at risk of this infection. Due to migration, it is estimated 750 000 infected carriers live in the USA or Europe.^531^^,^^532^ VA, especially sustained VT is closely related to high mortality, sudden death (SCD) happening in 17–50% of chronically ill patients.^533^ Based on the identification of different risk factors, Rassi *et al*. developed a mortality risk score (*Table 3*).^523^ Patients with HF, NYHA Class III/IV and NSVT on Holter and patients in NYHA Class I/II, with left ventricular dysfunction and NSVT on Holter are at the highest risk of death and should be regarded as candidates for aggressive therapeutic management.

**Table:** 

Patients with Chagas’ disease	Class	References
The Rassi score is useful in assessing risk of death in Chagas’ disease patients.		^523^ ^,^ ^524^
In patients with syncope and a BBB, an invasive EPS is useful in assessing risk of sustained ventricular arrhythmias.		^525^ ^,^ ^526^
When available, cardiac MRI with LGE should be considered to evaluate for arrhythmogenic substrate as part of a risk stratification strategy in those patients with cardiomyopathy.		^527–530^

**Table 3:** Rassi score

Risk factor				Points
NYHA Classes III or IV				5
Cardiomegaly (chest radiograph)				5
Segmental or global wall motion abnormality (2D echocardiogram)				3
Non-sustained ventricular tachycardia (24-h Holter)				3
Low QRS voltage (ECG)				2
Male sex				2
Total points	Total mortality (%)		Risk	
	5 years	10 years		
0–6	2	10	Low	
7–11	18	44	Intermediate	
12–20	63	84	High	

Conversely, patients with an abnormal ECG (right or left bundle branch conduction disorders) but in NYHA Class I/II HF without left ventricular dysfunction or NSVT on Holter are at lower risk of death. These patients should be followed up annually or biannually. Between these two extremes, some patients are at intermediate risk and their treatment strategies should be individualized.

Sustained VT has been reported as the main cause of syncope in patients with non-documented recurrent syncope and bundle branch block (BBB). In these cases, an EPS has been recommended for diagnosis elucidation.^525^ A finding of scar by LGE by cardiac MRI in patients with Chagas disease is considered a strong predictor of a combination of sustained VT and death.^534^

## How to assess risk for adverse outcomes in patients with ventricular tachyarrhythmia

### Risk for appropriate and inappropriate implantable cardioverter-defibrillator therapies

ICD therapies are associated with an increase in mortality.^370^^,^^422–424^ A single ICD shock is associated with a two- to five-fold increase in mortality, and progressive heart failure has been reported the most common cause of mortality among these patients.^425–427^ ICD therapies are classified as appropriate, inappropriate, avoidable, and phantom.^370^^,^^428^^,^^429^ Approximately 12–17% of patients receive inappropriate ICD shocks.^422^^,^^425–427^ Both appropriate and inappropriate shocks area associated with an increase in mortality and can significantly lower quality of life. Thus, identifying predictors of ICD therapies may improve quality of life and long-term outcomes in patients with ICDs.

#### Appropriate shock predictors

A previous episode of sustained VT correlates with high rate of appropriate shocks.^430–433^ A higher risk of appropriate therapy was seen in a secondary prevention ICD group when compared with a primary prevention ICD group at 5-year follow-up, while the rate of inappropriate therapy was comparable.^434^ Several studies have shown male sex as an independent risk factor for appropriate ICD therapies.^435^ Women are 30–50% less likely to receive an appropriate shock,^436–439^ and this difference is more pronounced among CRT-D recipients.^440–442^ However, most of studies have shown similar mortality rates in both genders after ICD implantation.^435–442^ AF is common in patients with left ventricular dysfunction; the prevalence can increase up to 50%. Worsening AF subtype increases the risk for both appropriate shocks and overall mortality.^443–446^

Other risk factors implicated to increase the risk of appropriate shocks are diabetes,^443^^,^^447^ elevated baseline NT-proBNP and BNP,^448^ NSVT,^445^^,^^449^ left atrial diameter,^443^^,^^449^ and impaired renal function.^450^ Data from SCD-HeFT and MADIT II trials have found a higher NYHA class, a lower LVEF, lack of use of beta-blocker therapy and single-chamber ICD as significant independent predictors for appropriate ICD shocks.^451^ Data from the Danish ICD Registry showed that LVEF <25% predicted an increased risks of both appropriate and inappropriate therapies.^452^

#### Inappropriate shock predictors

The presence of supraventricular tachycardias, in particular AF, has been reported as the most common risk factor for inappropriate ICD shocks.^426^^,^^444^^,^^445^ Another risk factor associated with inappropriate shock is younger age.^448^^,^^450^^,^^451^ Inappropriate shocks secondary to AF/atrial flutter are associated with increased mortality while inappropriate shocks related to sinus tachycardia or non-arrhythmic events like noise, artefact, and oversensing have shown similar survival as compared to those who do not receive a shock.^453^ Studies have failed to establish the superiority of dual-chamber ICD over the single chamber in reducing inappropriate shocks.^454^^,^^455^ The Danish ICD Registry showed a two-fold increase in the risk of inappropriate shocks associated with a dual-chamber ICD.^456^ Device technologies and programming, i.e. prolonged detection time, high rate programming, and better discrimination algorithms have markedly reduced the risk of inappropriate therapies.^370^^,^^456^^,^^457^

### Risk for heart failure incidence and progression

**Table:** 

Risk for heart failure incidence and progression	Class	References
Periodic monitoring of PVC burden (every 6 months) and LVEF and dimensions are useful in patients with frequent, asymptomatic PVCs and a normal LVEF and dimensions.		^458^
PVC burden exceeding 20% is associated with a higher risk of PVC-related cardiomyopathy.		^459–461^
PVC burden lower than 10% is associated with a lower risk of PVC-related cardiomyopathy.		^462^ ^,^ ^463^
In patients with PVC-related cardiomyopathy, absence of LGE on cardiac MRI may be used to identify patients with a favourable prognosis of left ventricular systolic function recovery.		^464–466^

Tachycardia-induced cardiomyopathy is a reversible cause of HF and impaired left ventricular function. Ventricular rhythms causing tachycardia-induced cardiomyopathy include VT, fascicular tachycardia, PVCs, and even persistent rapid DDD pacing. Left ventricular systolic function improves or normalizes and symptoms resolve, when tachycardia is corrected or controlled with medication or pharmacologic or non-pharmacologic rhythm control strategies.

Sustained monomorphic VT less commonly causes tachycardia-induced cardiomyopathy as compared to supraventricular tachycardias, since sustained VT is most often associated with some form of structural heart disease. When VT does lead to tachycardia-induced cardiomyopathy, it is by definition idiopathic and most commonly originates from the RVOT, left ventricular outflow tract, or coronary cusps. If these arrhythmias become persistent or high burden, they may cause reversible left ventricular dysfunction.^467^

A single centre series reported that 11% of patients who presented with frequent PVCs also had sustained monomorphic VT and 7% of those patients had tachycardia-induced cardiomyopathy. The presence of repetitive monomorphic VT was a significant predictor of tachycardia-induced cardiomyopathy development, particularly when it was the predominant arrhythmia on 24-h Holter monitoring.^461^

PVCs are very common and usually do not require treatment in the absence of symptoms. However, in the clinical setting of troublesome symptoms, or when PVCs trigger polymorphic VT or cause cardiomyopathy, proper treatment is critical. The concept of PVC-induced cardiomyopathy was first proposed by Duffee *et al.*,^460^ who observed a small group of patients with cardiomyopathy recover normal left ventricular function after pharmacological suppression of frequent PVCs.

Baman *et al.*^459^ reported on 174 consecutive patients referred for PVC ablation, 54 of whom had depressed left ventricular function. The authors concluded that although PVC-related cardiomyopathy may occur in patients with less PVCs, “in the presence of a PVC burden ≥24%, it may be helpful to suppress the PVCs by catheter ablation or drug therapy to avoid the development of cardiomyopathy.” However, Aki Lee *et al*., demonstrated a high rate of resolution of frequent PVCs among untreated patients with normal left ventricular function and minimal symptoms. A strategy of active surveillance is appropriate for the majority of patients with frequent idiopathic PVCs in association with preserved LVEF, owing to the low risk of developing left ventricular systolic dysfunction and the high rate of spontaneous resolution. Periodic monitoring of PVC burden and LVEF and dimensions can be useful in patients with frequent, asymptomatic PVCs and a normal LVEF and dimensions.^458^

It has become clear that comparative effectiveness trials are needed to understand what the best treatment approach is for patients with frequent PVCs and cardiomyopathy. A pilot multicentre study (PAPS: Prospective Assessment of PVC Suppression in Cardiomyopathy) is ongoing to better understand the prevalence of frequent PVCs and CM, and prove the feasibility of a large-scale randomized clinical trial (not yet published).^468^

Several circumstances have been associated with PVC-induced cardiomyopathy, including the PVC burden, asymptomatic status, duration of a high PVC burden, PVC QRS width >150 ms, interpolated PVCs, epicardial origin, and male gender. However, no prospective longitudinal assessments have been conducted that definitively prove their causal relation to PVC-induced cardiomyopathy.^469^

The diagnosis of tachycardia-induced cardiomyopathy or PVC-related cardiomyopathy can be challenging and the role of imaging modalities in the characterization of myocardial tissue as part of the diagnostic workup is limited.^464^ Cardiac MRI with LGE can accurately identify the presence and extent of myocardial scar and has become a first-line non-invasive imaging modality for the aetiologic assessment of primary cardiomyopathies and/or left ventricular systolic dysfunction, and could identify early stage of the structural heart disease.

### Risk for death in ventricular tachyarrhythmia patients

Risk prediction of death in VT patients has used numerous non-invasive and invasive markers including: clinical markers, mode of initial clinical presentation (e.g. sustained stable monomorphic VT, ventricular flutter, or VF), biomarkers, ECG abnormalities (e.g. left bundle branch block), heart rate variability, signal-averaged ECG, ambulatory ECG-based frequency domain T wave, microvolt level-T wave alternans, heart rate turbulence, heart rate deceleration, QT dispersion, cardiac autonomic function, echocardiographic evaluation of LVEF, left ventricular diameter, left ventricular mechanical dispersion by tissue Doppler, strain and velocity parameters to evaluate regional LV function, exercise testing to evaluate functional status, MRI to measure scar burden, and EPS to assess for inducibility of VT. Most of these tests and markers were applied to patients at risk of SCD and not patients who already have VT. Thus, their use for predicting death in a patient with VT is unknown.

**Table:** 

Risk for death in VT patients (including risk for SCD)	Class	References
Risk for SCD should be judged in each patient on a case-by-case basis and risk considered as a continuous variable rather than a dichotomized variable (high or low risk may change).		^71^ ^,^ ^470^ ^,^ ^471^
Individual risk assessment needs to be dynamic as the type and severity of risks can change over time (repeated measurements need to be made over time).		^472^
Risk assessment may include consideration of mode of death as the relative risk of non-sudden, non-cardiac death, sudden cardiac death, and non-sudden cardiac death is influenced by aging and worsening cardiomyopathy and cardiovascular risk factors.		^369^ ^,^ ^473^ ^,^ ^474^

The main sources of information about risk for SCD in patients with VT are from two studies from the era prior to widespread ICD use,^475^^,^^476^ the control groups (patients who did not receive ICDs) in the primary prevention ICD studies (MUSTT, MADIT, MADIT II, SCD-HeFT, DANISH, DEFINITE, CABage-PATCH, IRIS, DINAMIT) as well as analysis of large data samples from registries since ICD approval from Europe, Canada, and the USA.^70^^,^^477^ These data have been extensively reviewed to better characterize which variables predict the development of SCD and death in high-risk patients. Data from secondary prevention studies (AVID, CIDS, CASH) provide additional information about risk of death in patients who have had VT. Another source of information is the International VT Ablation Center Collaborative Study Group which analysed a large group of patients with VT (approximately 2000 patients from 12 international sites) undergoing catheter ablation.^478^ Finally, a third useful source of data is the Seattle Heart Failure model developed by Wayne Levy and his colleagues who analysed data from a large sample of heart failure patients to predict risk of death and SCD as well as create a model for predicting benefit from ICD therapy.^479^ This model has been prospectively validated among five additional study cohorts of almost 10 000 heart failure patients. It is important to recognize that the causes of death can change over time. For example, the risk of death in a patient with post-MI VT may be largely due to mechanical problems (VSD, mitral regurgitation, heart failure) in the first several weeks to months after MI and then 3–6 months later the risk of arrhythmic death may be much higher due to matured scar-mediated substrate.

Based on these studies, the risk factors for death in VT patients include increasing NYHA class, old age, female gender, electrical storm, frailty, diabetes mellitus, AF, chronic kidney disease, chronic obstructive lung disease, peripheral arterial disease, advanced HF, non-ICM, lower EF, multiple different VT morphologies, use of haemodynamic support devices during VT ablation, and poor functional status. These risk factors can be divided into risk factors related to non-cardiac disease (e.g. renal function, diabetes, COPD, peripheral arterial disease) which are powerful and determine mortality, and cardiac risk factors (ischaemic vs. non-ischaemic aetiology, multiple morphologies of VT, EF, and functional status). There was an interaction between variables, such as higher rates of both VT recurrence and mortality, which was observed in patients with lower EF and worse NYHA failure status.^478^^,^^479^

### Risk of adverse outcomes in patients treated with catheter ablation

**Table:** 

Risk of adverse outcomes in patients treated with catheter ablation	Class	References
The aetiology and severity of cardiomyopathy and inducibility of arrhythmias after VT ablation are useful in determining risk of recurrence of VT after catheter ablation.		^480^
Risk scores in combination with procedural characteristics may be useful for assessing adverse outcomes associated with catheter ablation of VT.		^481–483^

Risk of death or acute haemodynamic compromise in patients who undergo catheter ablation of ventricular arrhythmias is driven by patient-specific factors (comorbidities), procedural factors, and presentation of the patient. In a large retrospective multicentre registry, factors such as low EF, chronic kidney disease, VT storm, and unmappable VTs were associated with early mortality.^484^ As mentioned above, male sex is associated with occurrence of VT/VF and ICD shocks.^485^ As procedural factors are often difficult to determine prior to the procedure, various risk scores have been developed to assess risk of acute haemodynamic compromise and/or death in patients undergoing catheter ablation of VT. Of these, a modified version of the Seattle HF Model and PAINESD score have been used in single centre and multicentre retrospective studies to evaluate risk of acute haemodynamic compromise or death post-procedure.^481^^,^^482^^,^^484^ The Seattle HF Model incorporates, amongst other variables, age, EF, blood pressure, weight, gender, HF medications, blood electrolyte, and haemoglobin levels as well as NYHA to predict mortality. A modified version of this model which incorporates VT storm and ICD shocks was recently reported to be potentially more useful in predicting 6 months survival in patients who undergo VT ablation.^483^ The PAINESD score incorporates pulmonary disease, age, presence of ICM, NYHA, EF, VT storm, and diabetes and assigns a score between 3 and 6 to each of these patient characteristics. In retrospective studies, patients with a PAINESD score greater than 15 had a 24% risk of acute haemodynamic compromise and a significantly higher risk of mortality.^482^^,^^484^ Use of these risk scores can be important in discussion of risks and benefits in patients undergoing catheter ablation and may help determine need for haemodynamic support during the procedure. However, larger multicentre prospective studies are required. It is important to note that patients with lower EF and NYHA Class IV HF may still benefit from successful catheter ablation of VT, and freedom from VT after successful ablation is associated with improved mortality.^478^^,^^486^

With regard to VT recurrence, in addition to patient related comorbidities, large single centre and multicentre studies have shown that the risk of VT recurrence is driven by the underlying aetiology, particularly in patients with non-ischaemic heart disease, even after adjusting for other patient comorbidities.^487–489^ In particular, patients with Lamin A/C cardiomyopathy, hypertrophic cardiomyopathy, cardiac sarcoidosis, and valvular cardiomyopathy appear to be at higher risk for VT recurrence after catheter ablation as compared to idiopathic DCM.^480^^,^^487^ In addition, location of scar seems to determine risk of VT recurrence post-catheter ablation.^490^ In this regard, endocardial ablation alone may be insufficient in many non-ischaemic cardiomyopathies. In arrhythmogenic right ventricular cardiomyopathy, epicardial presence of scar can serve as the substrate for VT and combined endo-epicardal mapping and ablation or adjuvant epicardial ablation after endocardial ablation is often required.^491–494^ Cardiac MRI with LGE can be used in assessment of scar location and may be beneficial in diagnosis and peri-procedural planning of VT ablation.^495^

Retrospective studies have shown that inducibility of VT at the end of ablation is associated with adverse outcomes, even after adjusting for other patient comorbidities. Non-inducibility of VT in ICM patients was shown to be associated with improved arrhythmia-free survival rates and all-cause mortality,^496^^,^^497^ even after adjusting for other comorbidities. In addition, inducible clinical VT during non-invasive programmed electrical ventricular stimulation (PES) is associated with decreased 1-year VT free survival as compared with those who are not inducible (<30% vs. >80%)^498^

Patients who were non-inducible during non-invasive PES after ablation had a VT recurrence rate of only 9% at 1 year of follow-up when both acute (at the end of the procedure) and late (at 6 days post-procedure) programmed stimulation were negative.^499^ Therefore, PES may be used to guide redo ablation and address ICD programming.

Finally, although catheter ablation is generally performed after the occurrence of ICD therapies, two clinical trials reported the value of catheter ablation prior to or in conjunction with ICD implantation. The Prophylactic Catheter Ablation for Prevention of Defibrillator Therapy clinical trial randomized patients with spontaneous ventricular tachycardia or fibrillation and history of myocardial infarction to ICD or ICD and catheter ablation. In this trial, 30-day mortality was zero along with a significant reduction in ICD therapies from 31% to 9% between the control (ICD) and intervention arms (ICD + catheter ablation).^500^ The Catheter Ablation of Stable Ventricular Tachycardia before Defibrillator Implantation in Patients with Coronary Heart Disease (VTACH) trial randomized patients with history of myocardial infarction and stable VT to catheter ablation followed by ICD implantation vs. ICD implantation alone and showed that catheter ablation reduced occurrence of VT or VF by 18% at 2 years of follow-up. These data imply that in patients who receive ICD for secondary prevention and have ischaemic heart disease, catheter ablation can be considered earlier, at the time of ICD implantation, to reduce future ICD therapies and prior to potential presentation with VT storm.^501^ The impact of early ablation (at the time of ICD implantation) on mortality was the subject of the BERLIN-VT clinical trial, early results of which have indicated a lack of a difference in death or hospitalization for VT/VF in the deferred group (ablation after occurrence of third appropriate shock) vs. those who underwent prophylactic ablation at the time of ICD implantation.^502^ It is important to note that in these studies, patients had a history of VT or VF. In patients with ischaemic heart disease undergoing ICD implantation for primary prevention of sudden cardiac death, prophylactic substrate modification of scar by catheter ablation requires further investigation. In the Substrate Modification Study, patients randomized to ICD implantation plus VT ablation had similar time to VT recurrence as those who underwent ICD implantation only. However, catheter ablation at the time of ICD implantation was associated with a greater than 50% reduction in total number of ICD therapies throughout the follow-up period.^503^

## How to assess risk for adverse outcome in patients with other specific cardiac conditions

### Patients with ventricular premature contractions

**Table:** 

Patients with ventricular premature contractions	Class	References
An evaluation of cardiac function and screening for heart failure symptoms should be considered in patients with frequent ventricular ectopy (>10 000 PVCs within 24 h or >10% over a more extended timeframe).		^504^
An evaluation of cardiac function and screening for heart failure symptoms may be considered in patients with frequent multiform PVCs, PVCs with a QRS duration > 150 ms or PVCs with a coupling interval of <450 ms.		^505^ ^,^ ^506^

Frequent PVCs can lead to cardiomyopathy and HF, and are associated with increased mortality.^504^ In addition, in some patients with an inherited ACM, PVCs may be the initial clinical manifestation that leads to this diagnosis. An initial case series describing four patients who had reversal of cardiomyopathy after amiodarone successfully suppressed a high PVC burden has resulted in the recognition for the potential reversibility of this condition.^460^ However, only a minority of patients with PVCs will develop symptoms or adverse sequelae. The factors that can potentially predict development of HF and increased risk of adverse outcomes include PVC frequency as well as characteristics of the PVC morphology and timing of the PVC coupling interval.

#### Premature ventricular complex frequency

In a large cohort of patients, increased PVC frequency was associated with reduced LV function, a higher incidence of heart failure, and a higher risk of death. Specifically, compared to the lowest quartile of PVC frequency (<0.002%), the highest quartile (0.123% to 17.7%) in this cohort of patients with a structurally normal heart at baseline had a 31% increased risk of death over a follow-up of >13 years.^504^ Other studies correlating frequency with PVC-induced cardiomyopathy suggested a threshold effect observed at >20%, though there is no accepted cut-off that appears to be protective.^459^^,^^505^ In a study of 239 consecutive patients with apparently normal hearts, a PVC burden of >20 000 in 24 h was associated with a reduced LVEF, whereas >10 000 but <20 000 showed LV dilation with preserved LVEF.^507^

#### Premature ventricular complex morphology

In addition to PVC burden, the morphological features of the PVC have been evaluated. The width of the PVC QRS complex, perhaps reflective of dyssynchrony, has been associated with increased risk of developing PVC-induced cardiomyopathy.^505^^,^^506^ In these retrospective studies, patients with a PVC duration of >150 ms appeared to require a lower burden for development of a cardiomyopathy. A PVC duration of >153 ms in patients with a > 10% burden, was associated with an 82% sensitivity and 75% specificity for subsequent development of a cardiomyopathy. The presence of multiform PVCs has also been associated with the development of new onset heart failure.^508^

#### Premature ventricular complex coupling interval

One mechanism of PVC-induced cardiomyopathy may be due to ineffective mechanical contraction leading to adverse remodelling, possibly related to the timing of the PVC. However, there are only a few small studies evaluating this. In a retrospective cohort study of 510 patients, a PVC coupling interval of <450 ms was associated with a reduced LVEF.^509^ Another smaller study of 70 patients did not show any association, though its power was limited.^510^ Another study specifically identified the presence of interpolated PVCs regardless of coupling interval as associated with reduced LVEF.^511^ A short PVC coupling interval may also be an important determinant of VF, especially in patients with genetic or acquired early or abnormal repolarization.^42^^,^^512^^,^^513^

While the promise of effective treatment for reversing the potential adverse cardiac effects of frequent PVCs remains a possibility, it remains unclear whether such patients can easily be identified. Most cardiologists accept the dose–response relationship of PVC burden and reduced cardiac function, although the precise threshold for this effect remains unknown. There also is the potential for other factors aside from frequency alone, such as PVC QRS duration and coupling intervals, to influence adverse events associated with frequent PVCs.

### Patients with supraventricular tachyarrhythmia such as Wolff–Parkinson–White syndrome and focal atrial tachycardia

**Table:** 

Patients with supraventricular tachyarrhythmia such as WPW syndrome and focal atrial tachycardia	Class	References
EPS, with the use of isoprenaline, is recommended to risk stratify individuals with asymptomatic pre-excitation who have high-risk occupations/hobbies, and those who participate in competitive athletics.		^514–516^
EPS should be considered for risk stratification in asymptomatic pre-excitation patients without high-risk occupations or those who are not competitive athletes.		^514^ ^,^ ^516^ ^,^ ^517^
Non-invasive screening with exercise testing, drug testing, and ambulatory monitoring may be considered for risk stratification in asymptomatic pre-excitation patients without high-risk occupations or those who are not competitive athletes.		^514^ ^,^ ^516^ ^,^ ^517^
High-risk features to consider at EPS with or without catecholamine challenge are accessory pathways with an antegrade refractory period ≤250 ms, shortest pre-excited RR interval during AF ≤250 ms, inducible atrioventricular re-entrant tachycardia, and multiple accessory pathways.		^514^ ^,^ ^518^ ^,^ ^519^
Observation without treatment may be reasonable in asymptomatic WPW patients who are considered to be at low risk following EPS, abrupt loss of pre-excitation during exercise testing, or due to intermittent pre-excitation on a resting ECG or during ambulatory monitoring.		^514^ ^,^ ^516^

Patients with WPW may experience dramatic adverse events including SCD due to VF.^516^ The estimate for the frequency of SCD ranges up to 4% with more recent studies reporting a rate of 2%.^514^ Alarmingly, in approximately half of the patients SCD is the first clinical manifestation of the syndrome rendering appropriate risk stratification essential.^515^

Risk assessment strategies have been recently reviewed in the 2019 ESC Guidelines for the management of patients with supraventricular tachycardia.^520^ Main risk factors for the development of malignant arrhythmias and SCD in patients with pre-excitation are: (i) a short anterograde refractory period of the accessory pathway with the optimal cut-off reported to be at 250 ms and (ii) inducible atrioventricular reentrant tachycardia triggering pre-excited AF. A short pre-excited RR interval during AF ≤ 250 ms and the presence of multiple accessory pathways have been also reported as risk markers. For these reasons, EPS is recommended for risk stratification in subjects with asymptomatic ventricular pre-excitation who either have high-risk occupations or are competitive athletes. In patients without high-risk occupations or those who are not competitive athletes, EPS should be considered for risk stratification of patients with asymptomatic pre-excitation that can derive a prognostic benefit from prophylactic catheter ablation of the accessory pathway.^520^ Permanent Junctional Reciprocating Tachycardia (PJRT) re-presents a rare form of atrioventricular reciprocating tachycardia using a concealed accessory pathway. The incessant behaviour of PJRT may result in tachycardia-induced cardiomyopathy that usually resolves after successful treatment by RF catheter ablation.

Non-invasive testing may also be helpful. Non-invasive findings that identify a pathway not capable of maintaining rapid conduction during AF include intermittent loss of conduction over the accessory pathway on the resting ECG or during ambulatory monitoring, and abrupt loss of pre-excitation during exercise testing.^518^^,^^519^

Focal atrial tachycardias are characterized by regular atrial activation from atrial areas with centrifugal spread and can be classified as sustained or non-sustained. Sustained focal atria tachycardia in the adult population is usually associated with a benign prognosis, although tachycardia-mediated cardiomyopathy has been reported in up to 10% of patients referred for ablation of incessant SVT.^521^ Non-sustained atrial tachycardia is frequently found on Holter recordings and often does not require treatment; however, we should consider that patients with a high premature atrial complex burden (>500/24 h) are at increased risk for developing of AF and be educated on the symptoms of AF.^522^

## Summary

In clinical practice and for scientific purposes, cardiologists and primary care physicians perform risk assessment in patients with cardiac diseases or conditions with high risk of developing such.

The European Heart Rhythm Association (EHRA), Heart Rhythm Society (HRS), Asia Pacific Heart Rhythm Society (APHRS), and the Latin American Heart Rhythm Society (LAHRS) set down this expert consensus statement task force to summarize the consensus regarding risk assessment in cardiac arrhythmias. Objectives were to raise awareness of using the right risk assessment tool for a given outcome in a given population, and to provide physicians with practical proposals that may lead to rational and evidence-based risk assessment and improvement of patient care in this regard. A large variety of methods are used for risk assessment and choosing the best methods and tools hereof in a given situation is not simple. Even though parameters and test results found associated with increased risk of one outcome (e.g. death) may also be associated with higher risk of other adverse outcomes, specific risk assessment strategies should be used only for the purposes for which they are validated.

The work of this task force is summarized in a row of consensus statement tables.

## Supplementary material

Supplementary material is available at *Europace* online. 

**Conflict of interest**: J.C.N.: Personal research funding from Novo-Nordisk Foundation: Research in arrhythmia and device therapy (2018). Y.-J.L.: None declared. M.J.d.O.F.: Direct personal payment from Boehringer-Ingelheim: Dabigatran (2018) and Daiichi Sankyo: Edoxaban (2018). A.S.S.: None declared. A.A.: None declared. S.B.: Direct personal payment from Medtronic: Arrhythmias (ablation) (2018); Microport: Arrhythmias (ICD) (2018); Boston Scientific: Arrhythmias (S-ICD) (2018); Zoll Medical: WCD (2018). N.D.: Research funding from Abbott, Biotronik, Medtronic, Boston Scientific: Electrophysiology, arrhythmias (2018). D.D.T.: None declared. L.L.E.: Direct personal payment from Up to Date: Chapter on Cellular Arrhythmia Mechanisms (2018). K.E.: Direct personal payment from Biosense Webster: Research Grants, Consultant (2018); Biotronik: Speaker, Consultant (2018); Medtronic: Speaker, Consultant, Honoraria (2018); Boston Science: Speaker, Consultant, Honoraria (2018). Payment to department or institution from Abbott: Honoraria, Consulting (2018). Research funding from Biosense Webster: Ablation (2018); Medtronic: Device Research (2018); Boston Scientific: Research ablation and devices (2018). C.H.: Direct personal payment from Johnson & Johnson: Biosense Webster (2018). T.I.: Direct personal payment from Boehringer-Ingelheim: Honoraria (2018); Daiichi Sankyo: Honoraria (2018); Bayer Healthcare: Honoraria (2018); Bristol Myers Squibb: Honoraria (2018). A.J.: Direct personal payment from Abbott: CIED (2018); Medtronic: CIED (2018); Abbott Laboratories: Drugs (2018); Employment with Fortis Escorts Heart Institute, New Delhi (2018). E.K.: Research funding from Boehringer-Ingelheim: anticoagulation (2018); General Electric: electrocardiographic analysis (2018); Member of the Heart Rhythm Society, the American College of Cardiology, and the American Heart Association. A.K.: Direct personal payment from Medtronic: CIED (2018); Research funding from Medtronic: Sudden death (2018); President, Canadian Cardiovascular Society Secretary Treasurer, Heart Rhythm Society (2018). K.K.: Direct personal payment from Bayer: anticoagulation (2018); Daiichi Sankyo: anticoagulation (2018); Pfizer: anticoagulation (2018); Bristol Myers Squibb: anticoagulation (2018); Boston Scientific: Pacemaker/ICD (2018); Biotronik: Pacemaker/ICD (2018); Medtronic: Pacemkaer/ICD (2018); Payment from Boston Scientific: pacemaker/ICD (2018) and Medtronic: pacemaker/ICD (2018). V.K.: Direct personal payment from Biotronik: Home Monitoring (2018) and Zoll Medical: Women Initiative (2018); Payment from Duke Clinical Research Institute: CRT (2018); Research funding from Biotronik: CRT (2018); Boston Scientific: S-ICD (2018); Zoll Medical: WCD (2018). H.S.L.: Research funding from St Jude Medical: Research Support to Hospital (2018). G.L.: Payment to department or institution from Daiichi-Sankyo: Anticoagulation (2018); Bayer/Janssen: Anticoagulation (2018); Verseon: Anticoagulation development (2018); Boehringer Ingelheim: Anticoagulation; Registries; Steering Committees (2018); Pfizer: Anticoagulation; Registries (2018); BMS: Antithrombotic therapy (2018); Research funding from Boehringer-Ingelheim: AF registries [unrestricted educational grant] (2018); BMS/Pfizer: AF registries [unrestricted educational grant] (2018); Daiichi-Sankyo: Systematic reviews [unrestricted educational grant] (2018); Shares in private limited company (a legal separate entity in UK), but no salary/dividends/income/personal renumeration received (2018). S.N.T.: Direct personal payment from Biosense Webster: Electrophysiology (2018); Cook Medical: lead extraction (2018); Abbott: Pacing and Defibrillation. (2018). H.-N.P.: Secretary General, Asian Pacific Heart Rhythm Society, (2019-Present) Director of Policy and Insurance, Korean Heart Rhythm Society, (2017-Present) (2018); Director of Cardiac Intervention and Electrophysiology Laboratory, Severance Cardiovascular Hospital, (2014-Present) Outside Cooperation Committee Chair, Severance Cardiovascular Hospital, (2017-Present) (2018). G.R.D.: Direct personal payment from Medtronic: Cryoballon ablation proctoring (2018); Pfizer: Oral Anticoagulation (2018); Bayer Schering Pharma: Oral Anticoagulation (2018). W.S.: Direct personal payment from Boston Scientific: Catheter Ablation (2018); St Jude Medical: Catheter Ablation (2018); Biosense Webster: Catheter Ablation (2018); Research funding from Biosense Webster: Catheter Ablation (2018). A.S.: Direct personal payment from Abbott: Consultancy, Speaker Fees (2018); Bayer: Consultancy, Speaker Fees (2018); Boehringer-Ingelheim: Consultancy, Speaker Fees (2018); Boston Scientific: Consultancy, Speaker Fees (2018); Medtronic: Consultancy, Speaker Fees (2018); Novartis: Consultancy, Speaker Fees (2018); Pfizer: Consultancy, Speaker Fees (2018). J.H.S.: Direct personal payment from Medtronic: ICDs and pacemakers (2018); Research funding from Gilead: Antiarrhythmic medication (2018) and Medtronic: ICDs and pacemakers (2018); Consultant for Insurance company (2018). D.V.: Direct personal payment from St Jude Medical: Implantable Loop Recorder (2018). M.V.: None declared. A.W.: Payment to department or institution from Audentes (in 2017): gene therapy (2018); Coordinator of the European Reference Network (on rare cardiac diseases, GUARD-Heart) (2018). T.J.B.: Research funding from Boehringer-Ingelheim: Research Grant - Cognitive Atrial Fibrillation Trial (2018) and Boston Scientific: Research Grant - PLUG MRI trial (2018).

ESC Scientific Document Group: A.E.B.: Direct personal payment from Boston Scientific: Implantable Defibrillators (2018); Royalties from Wiley Blackwell: Cardiac Arrhythmia (2018). G.C.: None Declared. T.-F.C.: None Declared. L.E.: Direct Personal Payment From Abbott: Speaker Fees, Honoraria, Consultancy, Advisory Board Fees, Investigator, Committee Member (2018); Boehringer-Ingelheim: Speaker Fees, Honoraria, Consultancy, Advisory Board Fees, Investigator, Committee Member (2018); Boston Scientific: Speaker Fees, Honoraria, Consultancy, Advisory Board Fees, Investigator, Committee Member (2018); Daiichi Sankyo: Speaker Fees, Honoraria, Consultancy, Advisory Board Fees, Investigator, Committee Member (2018); Medtronic: Speaker Fees, Honoraria, Consultancy, Advisory Board Fees, Investigator, Committee Member (2018); Biotronik: Speaker Fees, Honoraria, Consultancy, Advisory Board Fees, Investigator, Committee Member (2018); Bayer Healthcare: Speaker Fees, Honoraria, Consultancy, Advisory Board Fees, Investigator, Committee Member (2018); Bristol Myers Squibb: Speaker Fees, Honoraria, Consultancy, Advisory Board Fees, Investigator, Committee Member (2018); Research Funding From Deutsche Forschungsgemeinschaft (2018) And German Cardiac Society (2018). H.E.: Direct personal payment from Boston Scientific: Honoraria fee (2018) and German Cardiac Society: Honoraria fee (2018). A.M.G.: Direct personal payment from Medtronic: Implantable Devices (2018); American Heart Association, Associate Editor Circulation Arrhythmias and Electrophysiology (2018). R.I.: None declared. J.K.: Direct personal payment from EPIX: ablation catheter (2018); Biosense Webster: catheters, mapping system (2018); Bayer: drugs (2018); Boehringer-Ingelheim: drugs (2018); Daiichi Sankyo: drugs (2018); Pfizer: drugs (2018); Bayer Healthcare: drugs (2018); MSD: drugs (2018); Boston Scientific: pacemakers, ICDs (2018); Biotronik: pacemakers, ICDs, catheters (2018); Liva nova (Sorin): pacemakers, ICDs, catheters (2018); Medtronic: pacemakers, ICDs, catheters, mapping system (2018); St Jude Medical (Abbott): pacemakers, ICDs, catheters, mapping system (2018). P.M.: Direct personal payment from Boston Scientific: EP (2018). J.D.M.: Direct personal payment from Boston Scientific: Electrophysiology (2018); Biosense Webster: Electrophysiology (2018); Vytronus, Inc: Electrophysiology (2018). G.-B.N.: None declared. B.O.: Direct personal payment from Lundbeck: Droxidopa (2018); Respironics: phrenic nerve stimulator (2018); Boehringer-Ingelheim: Pradaxa (2018). L.F.P.M.: None declared. M.Pi.: Direct personal payment from Daiichi Sankyo: Edoxaban (2018) and Bayer: Rivaroxaban (2018). M.Pr.: None declared. W.S.T.: Direct personal payment from Biotronik: CRM (2018); Abbott: CRM and Electrophysiology (2018); Boston Scientific: CRM and Electrophysiology (2018); Medtronic: CRM and Electrophysiology (2018); Biosense Webster: Electrophysiology mapping and ablation (2018); Electrophysiology Mapping and Ablation (2018); Payment to department or institution from Biosense Webster: Catheter Ablation (2018) and Abbott: Catheter Ablation and CRM (2018); Research funding from Abbott: Catheter Ablation (2018) and Boston Scientific: CRM (2018). P.S.: Direct personal payment from Abbott: Ablation (2018); Biosense Webster: Ablation (2018); Boehringer-Ingelheim: NOAC (2018); Daiichi Sankyo: NOAC (2018); Bayer Healthcare: NOAC (2018); Bristol Myers Squibb: NOAC (2018); Payment to department or institution from Abbott: Innovation (2018); Biosense Webster: Innovation (2018) J.S.: None declared. A.V.: None declared. T.D.: Direct personal payment from Herzklinik Bad Neustadt: Catheter ablation (2018); Research Funding from Herzklinik Bad Neustadt: Infrared Thermography Probe (2018).

## Supplementary Material

euaa065_Supplementary_Data

## Notes

Developed in partnership with and endorsed by the European Heart Rhythm Association (EHRA), a branch of the European Society of Cardiology (ESC), the Heart Rhythm Society (HRS), the Asia Pacific Heart Rhythm Society (APHRS), and the Latin American Heart Rhythm Society (LAHRS). This article has been co-published with permission in *EP Europace, Journal of Arrhythmia, Heart Rhythm, and Journal of Interventional Cardiac Electrophysiology*. © European Heart Rhythm Association, Asia Pacific Heart Rhythm Society, Heart Rhythm Society and Latin American Heart Rhythm Society, 2020. These articles are identical except for minor stylistic and spelling differences in keeping with each journal's style. Either citation can be used when citing this article.
