# Supplementary material for: European Heart Rhythm Association (EHRA)/Heart Rhythm Society (HRS)/Asia Pacific Heart Rhythm Society (APHRS)/Latin American Heart Rhythm Society (LAHRS) expert consensus on risk assessment in cardiac arrhythmias: use the right tool for the right outcome, in the right population
Source: Europace. 2020 Jun 15;22(8):1147–8. doi: 10.1093/europace/euaa065 (PMC7400488; doi:10.1093/europace/euaa065)
Supplement: euaa065_Supplementary_Data [file euaa065_supplementary_data.zip › DOI_Reviewed EHRA Consensus Doc on risk Assessment in Cardiac Arr CHAIR 2019.docx]

	Deneke Thomas 1- Financial Declaration  A - DIRECT PERSONAL PAYMENT AS IT APPLIES TO YOU AND YOUR SPOUSE/PARTNER OR ANY OTHER MEMBER OF YOUR HOUSEHOLD, OR ANY ENTITY CONTROLLED DIRECTLY OR INDIRECTLY BY ANY OF ABOVE PERSONS: SPEAKER FEES, HONORARIA, CONSULTANCY, ADVISORY BOARD FEES, INVESTIGATOR, COMMITTEE MEMBER, ETC. FROM HEALTHCARE INDUSTRY.                 - Herzklinik Bad Neustadt : Catheter ablation (2018)  D - RESEARCH FUNDING UNDER YOUR DIRECT/PERSONAL RESPONSIBILITY (TO DEPARTMENT OR INSTITUTION) FROM HEALTHCARE INDUSTRY.                 - Herzklinik Bad Neustadt : Infrared Thermography Probe (2018)	
Deneke Thomas	1- Financial Declaration
	A - DIRECT PERSONAL PAYMENT AS IT APPLIES TO YOU AND YOUR SPOUSE/PARTNER OR ANY OTHER MEMBER OF YOUR HOUSEHOLD, OR ANY ENTITY CONTROLLED DIRECTLY OR INDIRECTLY BY ANY OF ABOVE PERSONS: SPEAKER FEES, HONORARIA, CONSULTANCY, ADVISORY BOARD FEES, INVESTIGATOR, COMMITTEE MEMBER, ETC. FROM HEALTHCARE INDUSTRY.                 - Herzklinik Bad Neustadt : Catheter ablation (2018)
	D - RESEARCH FUNDING UNDER YOUR DIRECT/PERSONAL RESPONSIBILITY (TO DEPARTMENT OR INSTITUTION) FROM HEALTHCARE INDUSTRY.                 - Herzklinik Bad Neustadt : Infrared Thermography Probe (2018)
	
		
