# Supplementary material for: European Heart Rhythm Association (EHRA)/Heart Rhythm Society (HRS)/Asia Pacific Heart Rhythm Society (APHRS)/Latin American Heart Rhythm Society (LAHRS) expert consensus on risk assessment in cardiac arrhythmias: use the right tool for the right outcome, in the right population
Source: Europace. 2020 Jun 15;22(8):1147–8. doi: 10.1093/europace/euaa065 (PMC7400488; doi:10.1093/europace/euaa065)
Supplement: euaa065_Supplementary_Data [file euaa065_supplementary_data.zip › DOI_ReviewedDoiSummary EHRA HRS APHRS LAHRS Cons. doc on Risk Assessment- CHAIR 2019.docx]

	Nielsen Jens Cosedis 1- Financial Declaration  E - RESEARCH FUNDING (PERSONAL).                 - Novo-Nordisk Foundation : Research in arrhythmia and device therapy (2018)	
Nielsen Jens Cosedis	1- Financial Declaration
	E - RESEARCH FUNDING (PERSONAL).                 - Novo-Nordisk Foundation : Research in arrhythmia and device therapy (2018)
	
		
