# Supplementary material for: European Heart Rhythm Association (EHRA)/Heart Rhythm Society (HRS)/Asia Pacific Heart Rhythm Society (APHRS)/Latin American Heart Rhythm Society (LAHRS) expert consensus on risk assessment in cardiac arrhythmias: use the right tool for the right outcome, in the right population
Source: Europace. 2020 Jun 15;22(8):1147–8. doi: 10.1093/europace/euaa065 (PMC7400488; doi:10.1093/europace/euaa065)
Supplement: euaa065_Supplementary_Data [file euaa065_supplementary_data.zip › DOI_ReviewedDoiSummary EHRA HRS APHRS LAHRS Cons. doc on Risk Assessment- Writers 2019.docx]

	Alfie Alberto   Nothing to be declared (2018) Boveda Serge 1- Financial Declaration  A - DIRECT PERSONAL PAYMENT AS IT APPLIES TO YOU AND YOUR SPOUSE/PARTNER OR ANY OTHER MEMBER OF YOUR HOUSEHOLD, OR ANY ENTITY CONTROLLED DIRECTLY OR INDIRECTLY BY ANY OF ABOVE PERSONS: SPEAKER FEES, HONORARIA, CONSULTANCY, ADVISORY BOARD FEES, INVESTIGATOR, COMMITTEE MEMBER, ETC. FROM HEALTHCARE INDUSTRY.                 - Medtronic : Arrhythmias (ablation) (2018)                  - Microport : Arrhythmias (ICD) (2018)                  - Boston Scientific : Arrhythmias (S-ICD) (2018)                  - Zoll Medical : WCD (2018) Bunch T Jared 1- Financial Declaration  D - RESEARCH FUNDING UNDER YOUR DIRECT/PERSONAL RESPONSIBILITY (TO DEPARTMENT OR INSTITUTION) FROM HEALTHCARE INDUSTRY.                 - Boehringer-Ingelheim : Research Grant - Cognitive Atrial Fibrillation Trial (2018)                  - Boston Scientific : Research Grant - PLUG MRI trial (2018) Dagres Nikolaos 1- Financial Declaration  D - RESEARCH FUNDING UNDER YOUR DIRECT/PERSONAL RESPONSIBILITY (TO DEPARTMENT OR INSTITUTION) FROM HEALTHCARE INDUSTRY.                 - Abbott, Biotronik, Medtronic, Boston Scientific : Electrophysiology, arrhythmias (2018) Di Toro Dario   Nothing to be declared (2018) Eckhardt Lee 1- Financial Declaration  A - DIRECT PERSONAL PAYMENT AS IT APPLIES TO YOU AND YOUR SPOUSE/PARTNER OR ANY OTHER MEMBER OF YOUR HOUSEHOLD, OR ANY ENTITY CONTROLLED DIRECTLY OR INDIRECTLY BY ANY OF ABOVE PERSONS: SPEAKER FEES, HONORARIA, CONSULTANCY, ADVISORY BOARD FEES, INVESTIGATOR, COMMITTEE MEMBER, ETC. FROM HEALTHCARE INDUSTRY.                 - Up to Date : Chapter on Cellular Arrhythmia Mechanisms (2018) Ellenbogen Kenneth 1- Financial Declaration  A - DIRECT PERSONAL PAYMENT AS IT APPLIES TO YOU AND YOUR SPOUSE/PARTNER OR ANY OTHER MEMBER OF YOUR HOUSEHOLD, OR ANY ENTITY CONTROLLED DIRECTLY OR INDIRECTLY BY ANY OF ABOVE PERSONS: SPEAKER FEES, HONORARIA, CONSULTANCY, ADVISORY BOARD FEES, INVESTIGATOR, COMMITTEE MEMBER, ETC. FROM HEALTHCARE INDUSTRY.                 - Biosense Webster : Research Grants, Consultant (2018)                  - Biotronik : Speaker, Consultant (2018)                  - Medtronic : Speaker, Consultant, Honoraria (2018)                  - Boston Science : Speaker, Consultant, Honoraria (2018)  B - PAYMENT TO YOUR DEPARTMENT OR INSTITUTION OR ANY OTHER BODY LIKE AN ASSOCIATION OR SIMILAR FOR YOUR PERSONAL SERVICES: SPEAKER FEES, HONORARIA, CONSULTANCY, ADVISORY BOARD FEES, INVESTIGATOR, COMMITTEE MEMBER, ETC. FROM HEALTHCARE INDUSTRY.                 - Abbott : Honoraria, Consulting (2018)  D - RESEARCH FUNDING UNDER YOUR DIRECT/PERSONAL RESPONSIBILITY (TO DEPARTMENT OR INSTITUTION) FROM HEALTHCARE INDUSTRY.                 - Biosense Webster : Ablation  (2018)                  - Medtronic : Device Research (2018)                  - Boston Scientific : Research ablation and devices (2018) Figueiredo Marcio Jansen De Oliveira 1- Financial Declaration  A - DIRECT PERSONAL PAYMENT AS IT APPLIES TO YOU AND YOUR SPOUSE/PARTNER OR ANY OTHER MEMBER OF YOUR HOUSEHOLD, OR ANY ENTITY CONTROLLED DIRECTLY OR INDIRECTLY BY ANY OF ABOVE PERSONS: SPEAKER FEES, HONORARIA, CONSULTANCY, ADVISORY BOARD FEES, INVESTIGATOR, COMMITTEE MEMBER, ETC. FROM HEALTHCARE INDUSTRY.                 - Boehringer-Ingelheim : Dabigatran (2018)                  - Daiichi Sankyo : Edoxaban (2018) Hardy Carina Abigail 1- Financial Declaration  A - DIRECT PERSONAL PAYMENT AS IT APPLIES TO YOU AND YOUR SPOUSE/PARTNER OR ANY OTHER MEMBER OF YOUR HOUSEHOLD, OR ANY ENTITY CONTROLLED DIRECTLY OR INDIRECTLY BY ANY OF ABOVE PERSONS: SPEAKER FEES, HONORARIA, CONSULTANCY, ADVISORY BOARD FEES, INVESTIGATOR, COMMITTEE MEMBER, ETC. FROM HEALTHCARE INDUSTRY.                 - Johnson & Johnson : Biosense Webster  (2018) Ikeda Takanori 1- Financial Declaration  A - DIRECT PERSONAL PAYMENT AS IT APPLIES TO YOU AND YOUR SPOUSE/PARTNER OR ANY OTHER MEMBER OF YOUR HOUSEHOLD, OR ANY ENTITY CONTROLLED DIRECTLY OR INDIRECTLY BY ANY OF ABOVE PERSONS: SPEAKER FEES, HONORARIA, CONSULTANCY, ADVISORY BOARD FEES, INVESTIGATOR, COMMITTEE MEMBER, ETC. FROM HEALTHCARE INDUSTRY.                 - Boehringer-Ingelheim : Honoraria (2018)                  - Daiichi Sankyo : Honoraria (2018)                  - Bayer Healthcare : Honoraria (2018)                  - Bristol Myers Squibb : Honoraria (2018) Jaswal Aparna 1- Financial Declaration  A - DIRECT PERSONAL PAYMENT AS IT APPLIES TO YOU AND YOUR SPOUSE/PARTNER OR ANY OTHER MEMBER OF YOUR HOUSEHOLD, OR ANY ENTITY CONTROLLED DIRECTLY OR INDIRECTLY BY ANY OF ABOVE PERSONS: SPEAKER FEES, HONORARIA, CONSULTANCY, ADVISORY BOARD FEES, INVESTIGATOR, COMMITTEE MEMBER, ETC. FROM HEALTHCARE INDUSTRY.                 - Abbott : CIED (2018)                  - Medtronic : CIED (2018)                  - Abbott Laboratories : Drugs (2018)  2- Other Positions of Influence  2.2 - Employment in healthcare industry (including part time) during the year for which you are declaring.                 - Fortis Escorts Heart Institute, New Delhi (2018) Kaufman Elizabeth 1- Financial Declaration  D - RESEARCH FUNDING UNDER YOUR DIRECT/PERSONAL RESPONSIBILITY (TO DEPARTMENT OR INSTITUTION) FROM HEALTHCARE INDUSTRY.                 - Boehringer-Ingelheim : anticoagulation (2018)                  - General Electric : electrocardiographic analysis (2018)  2- Other Positions of Influence  2.3 - Membership or affiliation in political or advocacy groups  working in the field of cardiology.                 - I am a member of the Heart Rhythm Society, the American College of Cardiology, and the American Heart Association. All of these organizations do some political/advocacy work although I am not specifically involved in these activities. (2018) Krahn Andrew 1- Financial Declaration  A - DIRECT PERSONAL PAYMENT AS IT APPLIES TO YOU AND YOUR SPOUSE/PARTNER OR ANY OTHER MEMBER OF YOUR HOUSEHOLD, OR ANY ENTITY CONTROLLED DIRECTLY OR INDIRECTLY BY ANY OF ABOVE PERSONS: SPEAKER FEES, HONORARIA, CONSULTANCY, ADVISORY BOARD FEES, INVESTIGATOR, COMMITTEE MEMBER, ETC. FROM HEALTHCARE INDUSTRY.                 - Medtronic : CIED (2018)  D - RESEARCH FUNDING UNDER YOUR DIRECT/PERSONAL RESPONSIBILITY (TO DEPARTMENT OR INSTITUTION) FROM HEALTHCARE INDUSTRY.                 - Medtronic : Sudden death (2018)  2- Other Positions of Influence  2.3 - Membership or affiliation in political or advocacy groups  working in the field of cardiology.                 - President, Canadian Cardiovascular Society Secretary Treasurer, Heart Rhythm Society (2018) Kusano Kengo 1- Financial Declaration  A - DIRECT PERSONAL PAYMENT AS IT APPLIES TO YOU AND YOUR SPOUSE/PARTNER OR ANY OTHER MEMBER OF YOUR HOUSEHOLD, OR ANY ENTITY CONTROLLED DIRECTLY OR INDIRECTLY BY ANY OF ABOVE PERSONS: SPEAKER FEES, HONORARIA, CONSULTANCY, ADVISORY BOARD FEES, INVESTIGATOR, COMMITTEE MEMBER, ETC. FROM HEALTHCARE INDUSTRY.                 - Bayer : anticoagulation (2018)                  - Daiichi Sankyo : anticoagulation (2018)                  - Pfizer : anticoagulation (2018)                  - Bristol Myers Squibb : anticoagulation (2018)                  - Boston Scientific : Pacemaker/ICD (2018)                  - Biotronik : Pacemaker/ICD (2018)                  - Medtronic : Pacemkaer/ICD (2018)  B - PAYMENT TO YOUR DEPARTMENT OR INSTITUTION OR ANY OTHER BODY LIKE AN ASSOCIATION OR SIMILAR FOR YOUR PERSONAL SERVICES: SPEAKER FEES, HONORARIA, CONSULTANCY, ADVISORY BOARD FEES, INVESTIGATOR, COMMITTEE MEMBER, ETC. FROM HEALTHCARE INDUSTRY.                 - Boston Scientific : pacemaker/ICD (2018)                  - Medtronic : pacemaker/ICD (2018) Kutyifa Valentina 1- Financial Declaration  A - DIRECT PERSONAL PAYMENT AS IT APPLIES TO YOU AND YOUR SPOUSE/PARTNER OR ANY OTHER MEMBER OF YOUR HOUSEHOLD, OR ANY ENTITY CONTROLLED DIRECTLY OR INDIRECTLY BY ANY OF ABOVE PERSONS: SPEAKER FEES, HONORARIA, CONSULTANCY, ADVISORY BOARD FEES, INVESTIGATOR, COMMITTEE MEMBER, ETC. FROM HEALTHCARE INDUSTRY.                 - Biotronik : Home Monitoring (2018)                  - Zoll Medical : Women Initiative (2018)  B - PAYMENT TO YOUR DEPARTMENT OR INSTITUTION OR ANY OTHER BODY LIKE AN ASSOCIATION OR SIMILAR FOR YOUR PERSONAL SERVICES: SPEAKER FEES, HONORARIA, CONSULTANCY, ADVISORY BOARD FEES, INVESTIGATOR, COMMITTEE MEMBER, ETC. FROM HEALTHCARE INDUSTRY.                 - Duke Clinical Research Institute : CRT (2018)  D - RESEARCH FUNDING UNDER YOUR DIRECT/PERSONAL RESPONSIBILITY (TO DEPARTMENT OR INSTITUTION) FROM HEALTHCARE INDUSTRY.                 - Biotronik : CRT (2018)                  - Boston Scientific : S-ICD (2018)                  - Zoll Medical : WCD (2018) Lim Han Sung 1- Financial Declaration  D - RESEARCH FUNDING UNDER YOUR DIRECT/PERSONAL RESPONSIBILITY (TO DEPARTMENT OR INSTITUTION) FROM HEALTHCARE INDUSTRY.                 - St Jude Medical : Research Support to Hospital (2018) Lin Yenn-Jiang   Nothing to be declared (2018) Lip Gregory Yh 1- Financial Declaration  B - PAYMENT TO YOUR DEPARTMENT OR INSTITUTION OR ANY OTHER BODY LIKE AN ASSOCIATION OR SIMILAR FOR YOUR PERSONAL SERVICES: SPEAKER FEES, HONORARIA, CONSULTANCY, ADVISORY BOARD FEES, INVESTIGATOR, COMMITTEE MEMBER, ETC. FROM HEALTHCARE INDUSTRY.                 - Daiichi-Sankyo : Anticoagulation (2018)                  - Bayer/Janssen : Anticoagulation (2018)                  - Verseon : Anticoagulation development (2018)                  - Boehringer Ingelheim : Anticoagulation;  Registries; Steering Committees (2018)                  - Pfizer : Anticoagulation; Registries (2018)                  - BMS : Antithrombotic therapy (2018)  D - RESEARCH FUNDING UNDER YOUR DIRECT/PERSONAL RESPONSIBILITY (TO DEPARTMENT OR INSTITUTION) FROM HEALTHCARE INDUSTRY.                 - Boehringer-Ingelheim : AF registries [unrestricted educational grant] (2018)                  - BMS/Pfizer : AF registries [unrestricted educational grant] (2018)                  - Daiichi-Sankyo : Systematic reviews [unrestricted educational grant] (2018)  2- Other Positions of Influence  2.4 - Any other interest (financial or otherwise) that should be declared in view of holding an ESC position.                 - Shares in private limited company (a legal separate entity in UK), but no salary/dividends/income/personal renumeration received. (2018) Nava Townsend Santiago 1- Financial Declaration  A - DIRECT PERSONAL PAYMENT AS IT APPLIES TO YOU AND YOUR SPOUSE/PARTNER OR ANY OTHER MEMBER OF YOUR HOUSEHOLD, OR ANY ENTITY CONTROLLED DIRECTLY OR INDIRECTLY BY ANY OF ABOVE PERSONS: SPEAKER FEES, HONORARIA, CONSULTANCY, ADVISORY BOARD FEES, INVESTIGATOR, COMMITTEE MEMBER, ETC. FROM HEALTHCARE INDUSTRY.                 - Biosense Webster : Electrophysiology (2018)                  - Cook Medical : lead extraction (2018)                  - Abbott : Pacing and Defibrillation.  (2018) Pak Hui-Nam 2- Other Positions of Influence  2.3 - Membership or affiliation in political or advocacy groups  working in the field of cardiology.                 - Secretary General, Asian Pacific Heart Rhythm Society, (2019-Present.) Director of Policy and Insurance, Korean Heart Rhythm Society, (2017-Present.) (2018)  2.4 - Any other interest (financial or otherwise) that should be declared in view of holding an ESC position.                 - Director of Cardiac Intervention and Electrophysiology Laboratory, Severance Cardiovascular Hospital, (2014-Present.) Outside Cooperation Committee Chair, Severance Cardiovascular Hospital, (2017-Present.)  (2018) Rodriguez-Diez Gerardo 1- Financial Declaration  A - DIRECT PERSONAL PAYMENT AS IT APPLIES TO YOU AND YOUR SPOUSE/PARTNER OR ANY OTHER MEMBER OF YOUR HOUSEHOLD, OR ANY ENTITY CONTROLLED DIRECTLY OR INDIRECTLY BY ANY OF ABOVE PERSONS: SPEAKER FEES, HONORARIA, CONSULTANCY, ADVISORY BOARD FEES, INVESTIGATOR, COMMITTEE MEMBER, ETC. FROM HEALTHCARE INDUSTRY.                 - Medtronic : Cryoballon ablation proctoring (2018)                  - Pfizer : Oral Anticoagulation (2018)                  - Bayer Schering Pharma : Oral Anticoagulation (2018) Sauer William 1- Financial Declaration  A - DIRECT PERSONAL PAYMENT AS IT APPLIES TO YOU AND YOUR SPOUSE/PARTNER OR ANY OTHER MEMBER OF YOUR HOUSEHOLD, OR ANY ENTITY CONTROLLED DIRECTLY OR INDIRECTLY BY ANY OF ABOVE PERSONS: SPEAKER FEES, HONORARIA, CONSULTANCY, ADVISORY BOARD FEES, INVESTIGATOR, COMMITTEE MEMBER, ETC. FROM HEALTHCARE INDUSTRY.                 - Boston Scientific : Catheter Ablation (2018)                  - St Jude Medical : Catheter Ablation (2018)                  - Biosense Webster : Catheter Ablation (2018)  D - RESEARCH FUNDING UNDER YOUR DIRECT/PERSONAL RESPONSIBILITY (TO DEPARTMENT OR INSTITUTION) FROM HEALTHCARE INDUSTRY.                 - Biosense Webster : Catheter Ablation (2018) Saxena Anil 1- Financial Declaration  A - DIRECT PERSONAL PAYMENT AS IT APPLIES TO YOU AND YOUR SPOUSE/PARTNER OR ANY OTHER MEMBER OF YOUR HOUSEHOLD, OR ANY ENTITY CONTROLLED DIRECTLY OR INDIRECTLY BY ANY OF ABOVE PERSONS: SPEAKER FEES, HONORARIA, CONSULTANCY, ADVISORY BOARD FEES, INVESTIGATOR, COMMITTEE MEMBER, ETC. FROM HEALTHCARE INDUSTRY.                 - Abbott : Consultancy, Speaker Fees (2018)                  - Bayer : Consultancy, Speaker Fees (2018)                  - Boehringer-Ingelheim : Consultancy, Speaker Fees (2018)                  - Boston Scientific : Consultancy, Speaker Fees (2018)                  - Medtronic : Consultancy, Speaker Fees (2018)                  - Novartis : Consultancy, Speaker Fees (2018)                  - Pfizer : Consultancy, Speaker Fees (2018) Sepehri Shamloo Alireza   Nothing to be declared (2018) Svendsen Jesper Hastrup 1- Financial Declaration  A - DIRECT PERSONAL PAYMENT AS IT APPLIES TO YOU AND YOUR SPOUSE/PARTNER OR ANY OTHER MEMBER OF YOUR HOUSEHOLD, OR ANY ENTITY CONTROLLED DIRECTLY OR INDIRECTLY BY ANY OF ABOVE PERSONS: SPEAKER FEES, HONORARIA, CONSULTANCY, ADVISORY BOARD FEES, INVESTIGATOR, COMMITTEE MEMBER, ETC. FROM HEALTHCARE INDUSTRY.                 - Medtronic : ICDs and pacemakers (2018)  D - RESEARCH FUNDING UNDER YOUR DIRECT/PERSONAL RESPONSIBILITY (TO DEPARTMENT OR INSTITUTION) FROM HEALTHCARE INDUSTRY.                 - Gilead : Antiarrhythmic medication (2018)                  - Medtronic : ICDs and pacemakers (2018)  2- Other Positions of Influence  2.2 - Employment in healthcare industry (including part time) during the year for which you are declaring.                 - Consultant for Insurance company (2018) Vanegas Diego 1- Financial Declaration  A - DIRECT PERSONAL PAYMENT AS IT APPLIES TO YOU AND YOUR SPOUSE/PARTNER OR ANY OTHER MEMBER OF YOUR HOUSEHOLD, OR ANY ENTITY CONTROLLED DIRECTLY OR INDIRECTLY BY ANY OF ABOVE PERSONS: SPEAKER FEES, HONORARIA, CONSULTANCY, ADVISORY BOARD FEES, INVESTIGATOR, COMMITTEE MEMBER, ETC. FROM HEALTHCARE INDUSTRY.                 - St Jude Medical : Implantable Loop Recorder (2018) Vaseghi Marmar   Nothing to be declared (2018) Wilde Arthur 1- Financial Declaration  B - PAYMENT TO YOUR DEPARTMENT OR INSTITUTION OR ANY OTHER BODY LIKE AN ASSOCIATION OR SIMILAR FOR YOUR PERSONAL SERVICES: SPEAKER FEES, HONORARIA, CONSULTANCY, ADVISORY BOARD FEES, INVESTIGATOR, COMMITTEE MEMBER, ETC. FROM HEALTHCARE INDUSTRY.                 - Audentes (in 2017) : gene therapy (2018)  2- Other Positions of Influence  2.3 - Membership or affiliation in political or advocacy groups  working in the field of cardiology.                 - Coordinator of the European Reference Network (on rare cardiac diseases, GUARD-Heart) (2018)	
Alfie Alberto	
	Nothing to be declared (2018)
Boveda Serge	1- Financial Declaration
	A - DIRECT PERSONAL PAYMENT AS IT APPLIES TO YOU AND YOUR SPOUSE/PARTNER OR ANY OTHER MEMBER OF YOUR HOUSEHOLD, OR ANY ENTITY CONTROLLED DIRECTLY OR INDIRECTLY BY ANY OF ABOVE PERSONS: SPEAKER FEES, HONORARIA, CONSULTANCY, ADVISORY BOARD FEES, INVESTIGATOR, COMMITTEE MEMBER, ETC. FROM HEALTHCARE INDUSTRY.                 - Medtronic : Arrhythmias (ablation) (2018)
	- Microport : Arrhythmias (ICD) (2018)
	- Boston Scientific : Arrhythmias (S-ICD) (2018)
	- Zoll Medical : WCD (2018)
Bunch T Jared	1- Financial Declaration
	D - RESEARCH FUNDING UNDER YOUR DIRECT/PERSONAL RESPONSIBILITY (TO DEPARTMENT OR INSTITUTION) FROM HEALTHCARE INDUSTRY.                 - Boehringer-Ingelheim : Research Grant - Cognitive Atrial Fibrillation Trial (2018)
	- Boston Scientific : Research Grant - PLUG MRI trial (2018)
Dagres Nikolaos	1- Financial Declaration
	D - RESEARCH FUNDING UNDER YOUR DIRECT/PERSONAL RESPONSIBILITY (TO DEPARTMENT OR INSTITUTION) FROM HEALTHCARE INDUSTRY.                 - Abbott, Biotronik, Medtronic, Boston Scientific : Electrophysiology, arrhythmias (2018)
Di Toro Dario	
	Nothing to be declared (2018)
Eckhardt Lee	1- Financial Declaration
	A - DIRECT PERSONAL PAYMENT AS IT APPLIES TO YOU AND YOUR SPOUSE/PARTNER OR ANY OTHER MEMBER OF YOUR HOUSEHOLD, OR ANY ENTITY CONTROLLED DIRECTLY OR INDIRECTLY BY ANY OF ABOVE PERSONS: SPEAKER FEES, HONORARIA, CONSULTANCY, ADVISORY BOARD FEES, INVESTIGATOR, COMMITTEE MEMBER, ETC. FROM HEALTHCARE INDUSTRY.                 - Up to Date : Chapter on Cellular Arrhythmia Mechanisms (2018)
Ellenbogen Kenneth	1- Financial Declaration
	A - DIRECT PERSONAL PAYMENT AS IT APPLIES TO YOU AND YOUR SPOUSE/PARTNER OR ANY OTHER MEMBER OF YOUR HOUSEHOLD, OR ANY ENTITY CONTROLLED DIRECTLY OR INDIRECTLY BY ANY OF ABOVE PERSONS: SPEAKER FEES, HONORARIA, CONSULTANCY, ADVISORY BOARD FEES, INVESTIGATOR, COMMITTEE MEMBER, ETC. FROM HEALTHCARE INDUSTRY.                 - Biosense Webster : Research Grants, Consultant (2018)
	- Biotronik : Speaker, Consultant (2018)
	- Medtronic : Speaker, Consultant, Honoraria (2018)
	- Boston Science : Speaker, Consultant, Honoraria (2018)
	B - PAYMENT TO YOUR DEPARTMENT OR INSTITUTION OR ANY OTHER BODY LIKE AN ASSOCIATION OR SIMILAR FOR YOUR PERSONAL SERVICES: SPEAKER FEES, HONORARIA, CONSULTANCY, ADVISORY BOARD FEES, INVESTIGATOR, COMMITTEE MEMBER, ETC. FROM HEALTHCARE INDUSTRY.                 - Abbott : Honoraria, Consulting (2018)
	D - RESEARCH FUNDING UNDER YOUR DIRECT/PERSONAL RESPONSIBILITY (TO DEPARTMENT OR INSTITUTION) FROM HEALTHCARE INDUSTRY.                 - Biosense Webster : Ablation  (2018)
	- Medtronic : Device Research (2018)
	- Boston Scientific : Research ablation and devices (2018)
Figueiredo Marcio Jansen De Oliveira	1- Financial Declaration
	A - DIRECT PERSONAL PAYMENT AS IT APPLIES TO YOU AND YOUR SPOUSE/PARTNER OR ANY OTHER MEMBER OF YOUR HOUSEHOLD, OR ANY ENTITY CONTROLLED DIRECTLY OR INDIRECTLY BY ANY OF ABOVE PERSONS: SPEAKER FEES, HONORARIA, CONSULTANCY, ADVISORY BOARD FEES, INVESTIGATOR, COMMITTEE MEMBER, ETC. FROM HEALTHCARE INDUSTRY.                 - Boehringer-Ingelheim : Dabigatran (2018)
	- Daiichi Sankyo : Edoxaban (2018)
Hardy Carina Abigail	1- Financial Declaration
	A - DIRECT PERSONAL PAYMENT AS IT APPLIES TO YOU AND YOUR SPOUSE/PARTNER OR ANY OTHER MEMBER OF YOUR HOUSEHOLD, OR ANY ENTITY CONTROLLED DIRECTLY OR INDIRECTLY BY ANY OF ABOVE PERSONS: SPEAKER FEES, HONORARIA, CONSULTANCY, ADVISORY BOARD FEES, INVESTIGATOR, COMMITTEE MEMBER, ETC. FROM HEALTHCARE INDUSTRY.                 - Johnson & Johnson : Biosense Webster  (2018)
Ikeda Takanori	1- Financial Declaration
	A - DIRECT PERSONAL PAYMENT AS IT APPLIES TO YOU AND YOUR SPOUSE/PARTNER OR ANY OTHER MEMBER OF YOUR HOUSEHOLD, OR ANY ENTITY CONTROLLED DIRECTLY OR INDIRECTLY BY ANY OF ABOVE PERSONS: SPEAKER FEES, HONORARIA, CONSULTANCY, ADVISORY BOARD FEES, INVESTIGATOR, COMMITTEE MEMBER, ETC. FROM HEALTHCARE INDUSTRY.                 - Boehringer-Ingelheim : Honoraria (2018)
	- Daiichi Sankyo : Honoraria (2018)
	- Bayer Healthcare : Honoraria (2018)
	- Bristol Myers Squibb : Honoraria (2018)
Jaswal Aparna	1- Financial Declaration
	A - DIRECT PERSONAL PAYMENT AS IT APPLIES TO YOU AND YOUR SPOUSE/PARTNER OR ANY OTHER MEMBER OF YOUR HOUSEHOLD, OR ANY ENTITY CONTROLLED DIRECTLY OR INDIRECTLY BY ANY OF ABOVE PERSONS: SPEAKER FEES, HONORARIA, CONSULTANCY, ADVISORY BOARD FEES, INVESTIGATOR, COMMITTEE MEMBER, ETC. FROM HEALTHCARE INDUSTRY.                 - Abbott : CIED (2018)
	- Medtronic : CIED (2018)
	- Abbott Laboratories : Drugs (2018)
	2- Other Positions of Influence
	2.2 - Employment in healthcare industry (including part time) during the year for which you are declaring.                 - Fortis Escorts Heart Institute, New Delhi (2018)
Kaufman Elizabeth	1- Financial Declaration
	D - RESEARCH FUNDING UNDER YOUR DIRECT/PERSONAL RESPONSIBILITY (TO DEPARTMENT OR INSTITUTION) FROM HEALTHCARE INDUSTRY.                 - Boehringer-Ingelheim : anticoagulation (2018)
	- General Electric : electrocardiographic analysis (2018)
	2- Other Positions of Influence
	2.3 - Membership or affiliation in political or advocacy groups  working in the field of cardiology.                 - I am a member of the Heart Rhythm Society, the American College of Cardiology, and the American Heart Association. All of these organizations do some political/advocacy work although I am not specifically involved in these activities. (2018)
Krahn Andrew	1- Financial Declaration
	A - DIRECT PERSONAL PAYMENT AS IT APPLIES TO YOU AND YOUR SPOUSE/PARTNER OR ANY OTHER MEMBER OF YOUR HOUSEHOLD, OR ANY ENTITY CONTROLLED DIRECTLY OR INDIRECTLY BY ANY OF ABOVE PERSONS: SPEAKER FEES, HONORARIA, CONSULTANCY, ADVISORY BOARD FEES, INVESTIGATOR, COMMITTEE MEMBER, ETC. FROM HEALTHCARE INDUSTRY.                 - Medtronic : CIED (2018)
	D - RESEARCH FUNDING UNDER YOUR DIRECT/PERSONAL RESPONSIBILITY (TO DEPARTMENT OR INSTITUTION) FROM HEALTHCARE INDUSTRY.                 - Medtronic : Sudden death (2018)
	2- Other Positions of Influence
	2.3 - Membership or affiliation in political or advocacy groups  working in the field of cardiology.                 - President, Canadian Cardiovascular Society Secretary Treasurer, Heart Rhythm Society (2018)
Kusano Kengo	1- Financial Declaration
	A - DIRECT PERSONAL PAYMENT AS IT APPLIES TO YOU AND YOUR SPOUSE/PARTNER OR ANY OTHER MEMBER OF YOUR HOUSEHOLD, OR ANY ENTITY CONTROLLED DIRECTLY OR INDIRECTLY BY ANY OF ABOVE PERSONS: SPEAKER FEES, HONORARIA, CONSULTANCY, ADVISORY BOARD FEES, INVESTIGATOR, COMMITTEE MEMBER, ETC. FROM HEALTHCARE INDUSTRY.                 - Bayer : anticoagulation (2018)
	- Daiichi Sankyo : anticoagulation (2018)
	- Pfizer : anticoagulation (2018)
	- Bristol Myers Squibb : anticoagulation (2018)
	- Boston Scientific : Pacemaker/ICD (2018)
	- Biotronik : Pacemaker/ICD (2018)
	- Medtronic : Pacemkaer/ICD (2018)
	B - PAYMENT TO YOUR DEPARTMENT OR INSTITUTION OR ANY OTHER BODY LIKE AN ASSOCIATION OR SIMILAR FOR YOUR PERSONAL SERVICES: SPEAKER FEES, HONORARIA, CONSULTANCY, ADVISORY BOARD FEES, INVESTIGATOR, COMMITTEE MEMBER, ETC. FROM HEALTHCARE INDUSTRY.                 - Boston Scientific : pacemaker/ICD (2018)
	- Medtronic : pacemaker/ICD (2018)
Kutyifa Valentina	1- Financial Declaration
	A - DIRECT PERSONAL PAYMENT AS IT APPLIES TO YOU AND YOUR SPOUSE/PARTNER OR ANY OTHER MEMBER OF YOUR HOUSEHOLD, OR ANY ENTITY CONTROLLED DIRECTLY OR INDIRECTLY BY ANY OF ABOVE PERSONS: SPEAKER FEES, HONORARIA, CONSULTANCY, ADVISORY BOARD FEES, INVESTIGATOR, COMMITTEE MEMBER, ETC. FROM HEALTHCARE INDUSTRY.                 - Biotronik : Home Monitoring (2018)
	- Zoll Medical : Women Initiative (2018)
	B - PAYMENT TO YOUR DEPARTMENT OR INSTITUTION OR ANY OTHER BODY LIKE AN ASSOCIATION OR SIMILAR FOR YOUR PERSONAL SERVICES: SPEAKER FEES, HONORARIA, CONSULTANCY, ADVISORY BOARD FEES, INVESTIGATOR, COMMITTEE MEMBER, ETC. FROM HEALTHCARE INDUSTRY.                 - Duke Clinical Research Institute : CRT (2018)
	D - RESEARCH FUNDING UNDER YOUR DIRECT/PERSONAL RESPONSIBILITY (TO DEPARTMENT OR INSTITUTION) FROM HEALTHCARE INDUSTRY.                 - Biotronik : CRT (2018)
	- Boston Scientific : S-ICD (2018)
	- Zoll Medical : WCD (2018)
Lim Han Sung	1- Financial Declaration
	D - RESEARCH FUNDING UNDER YOUR DIRECT/PERSONAL RESPONSIBILITY (TO DEPARTMENT OR INSTITUTION) FROM HEALTHCARE INDUSTRY.                 - St Jude Medical : Research Support to Hospital (2018)
Lin Yenn-Jiang	
	Nothing to be declared (2018)
Lip Gregory Yh	1- Financial Declaration
	B - PAYMENT TO YOUR DEPARTMENT OR INSTITUTION OR ANY OTHER BODY LIKE AN ASSOCIATION OR SIMILAR FOR YOUR PERSONAL SERVICES: SPEAKER FEES, HONORARIA, CONSULTANCY, ADVISORY BOARD FEES, INVESTIGATOR, COMMITTEE MEMBER, ETC. FROM HEALTHCARE INDUSTRY.                 - Daiichi-Sankyo : Anticoagulation (2018)
	- Bayer/Janssen : Anticoagulation (2018)
	- Verseon : Anticoagulation development (2018)
	- Boehringer Ingelheim : Anticoagulation;  Registries; Steering Committees (2018)
	- Pfizer : Anticoagulation; Registries (2018)
	- BMS : Antithrombotic therapy (2018)
	D - RESEARCH FUNDING UNDER YOUR DIRECT/PERSONAL RESPONSIBILITY (TO DEPARTMENT OR INSTITUTION) FROM HEALTHCARE INDUSTRY.                 - Boehringer-Ingelheim : AF registries [unrestricted educational grant] (2018)
	- BMS/Pfizer : AF registries [unrestricted educational grant] (2018)
	- Daiichi-Sankyo : Systematic reviews [unrestricted educational grant] (2018)
	2- Other Positions of Influence
	2.4 - Any other interest (financial or otherwise) that should be declared in view of holding an ESC position.                 - Shares in private limited company (a legal separate entity in UK), but no salary/dividends/income/personal renumeration received. (2018)
Nava Townsend Santiago	1- Financial Declaration
	A - DIRECT PERSONAL PAYMENT AS IT APPLIES TO YOU AND YOUR SPOUSE/PARTNER OR ANY OTHER MEMBER OF YOUR HOUSEHOLD, OR ANY ENTITY CONTROLLED DIRECTLY OR INDIRECTLY BY ANY OF ABOVE PERSONS: SPEAKER FEES, HONORARIA, CONSULTANCY, ADVISORY BOARD FEES, INVESTIGATOR, COMMITTEE MEMBER, ETC. FROM HEALTHCARE INDUSTRY.                 - Biosense Webster : Electrophysiology (2018)
	- Cook Medical : lead extraction (2018)
	- Abbott : Pacing and Defibrillation.  (2018)
Pak Hui-Nam	2- Other Positions of Influence
	2.3 - Membership or affiliation in political or advocacy groups  working in the field of cardiology.                 - Secretary General, Asian Pacific Heart Rhythm Society, (2019-Present.) Director of Policy and Insurance, Korean Heart Rhythm Society, (2017-Present.) (2018)
	2.4 - Any other interest (financial or otherwise) that should be declared in view of holding an ESC position.                 - Director of Cardiac Intervention and Electrophysiology Laboratory, Severance Cardiovascular Hospital, (2014-Present.) Outside Cooperation Committee Chair, Severance Cardiovascular Hospital, (2017-Present.)  (2018)
Rodriguez-Diez Gerardo	1- Financial Declaration
	A - DIRECT PERSONAL PAYMENT AS IT APPLIES TO YOU AND YOUR SPOUSE/PARTNER OR ANY OTHER MEMBER OF YOUR HOUSEHOLD, OR ANY ENTITY CONTROLLED DIRECTLY OR INDIRECTLY BY ANY OF ABOVE PERSONS: SPEAKER FEES, HONORARIA, CONSULTANCY, ADVISORY BOARD FEES, INVESTIGATOR, COMMITTEE MEMBER, ETC. FROM HEALTHCARE INDUSTRY.                 - Medtronic : Cryoballon ablation proctoring (2018)
	- Pfizer : Oral Anticoagulation (2018)
	- Bayer Schering Pharma : Oral Anticoagulation (2018)
Sauer William	1- Financial Declaration
	A - DIRECT PERSONAL PAYMENT AS IT APPLIES TO YOU AND YOUR SPOUSE/PARTNER OR ANY OTHER MEMBER OF YOUR HOUSEHOLD, OR ANY ENTITY CONTROLLED DIRECTLY OR INDIRECTLY BY ANY OF ABOVE PERSONS: SPEAKER FEES, HONORARIA, CONSULTANCY, ADVISORY BOARD FEES, INVESTIGATOR, COMMITTEE MEMBER, ETC. FROM HEALTHCARE INDUSTRY.                 - Boston Scientific : Catheter Ablation (2018)
	- St Jude Medical : Catheter Ablation (2018)
	- Biosense Webster : Catheter Ablation (2018)
	D - RESEARCH FUNDING UNDER YOUR DIRECT/PERSONAL RESPONSIBILITY (TO DEPARTMENT OR INSTITUTION) FROM HEALTHCARE INDUSTRY.                 - Biosense Webster : Catheter Ablation (2018)
Saxena Anil	1- Financial Declaration
	A - DIRECT PERSONAL PAYMENT AS IT APPLIES TO YOU AND YOUR SPOUSE/PARTNER OR ANY OTHER MEMBER OF YOUR HOUSEHOLD, OR ANY ENTITY CONTROLLED DIRECTLY OR INDIRECTLY BY ANY OF ABOVE PERSONS: SPEAKER FEES, HONORARIA, CONSULTANCY, ADVISORY BOARD FEES, INVESTIGATOR, COMMITTEE MEMBER, ETC. FROM HEALTHCARE INDUSTRY.                 - Abbott : Consultancy, Speaker Fees (2018)
	- Bayer : Consultancy, Speaker Fees (2018)
	- Boehringer-Ingelheim : Consultancy, Speaker Fees (2018)
	- Boston Scientific : Consultancy, Speaker Fees (2018)
	- Medtronic : Consultancy, Speaker Fees (2018)
	- Novartis : Consultancy, Speaker Fees (2018)
	- Pfizer : Consultancy, Speaker Fees (2018)
Sepehri Shamloo Alireza	
	Nothing to be declared (2018)
Svendsen Jesper Hastrup	1- Financial Declaration
	A - DIRECT PERSONAL PAYMENT AS IT APPLIES TO YOU AND YOUR SPOUSE/PARTNER OR ANY OTHER MEMBER OF YOUR HOUSEHOLD, OR ANY ENTITY CONTROLLED DIRECTLY OR INDIRECTLY BY ANY OF ABOVE PERSONS: SPEAKER FEES, HONORARIA, CONSULTANCY, ADVISORY BOARD FEES, INVESTIGATOR, COMMITTEE MEMBER, ETC. FROM HEALTHCARE INDUSTRY.                 - Medtronic : ICDs and pacemakers (2018)
	D - RESEARCH FUNDING UNDER YOUR DIRECT/PERSONAL RESPONSIBILITY (TO DEPARTMENT OR INSTITUTION) FROM HEALTHCARE INDUSTRY.                 - Gilead : Antiarrhythmic medication (2018)
	- Medtronic : ICDs and pacemakers (2018)
	2- Other Positions of Influence
	2.2 - Employment in healthcare industry (including part time) during the year for which you are declaring.                 - Consultant for Insurance company (2018)
Vanegas Diego	1- Financial Declaration
	A - DIRECT PERSONAL PAYMENT AS IT APPLIES TO YOU AND YOUR SPOUSE/PARTNER OR ANY OTHER MEMBER OF YOUR HOUSEHOLD, OR ANY ENTITY CONTROLLED DIRECTLY OR INDIRECTLY BY ANY OF ABOVE PERSONS: SPEAKER FEES, HONORARIA, CONSULTANCY, ADVISORY BOARD FEES, INVESTIGATOR, COMMITTEE MEMBER, ETC. FROM HEALTHCARE INDUSTRY.                 - St Jude Medical : Implantable Loop Recorder (2018)
Vaseghi Marmar	
	Nothing to be declared (2018)
Wilde Arthur	1- Financial Declaration
	B - PAYMENT TO YOUR DEPARTMENT OR INSTITUTION OR ANY OTHER BODY LIKE AN ASSOCIATION OR SIMILAR FOR YOUR PERSONAL SERVICES: SPEAKER FEES, HONORARIA, CONSULTANCY, ADVISORY BOARD FEES, INVESTIGATOR, COMMITTEE MEMBER, ETC. FROM HEALTHCARE INDUSTRY.                 - Audentes (in 2017) : gene therapy (2018)
	2- Other Positions of Influence
	2.3 - Membership or affiliation in political or advocacy groups  working in the field of cardiology.                 - Coordinator of the European Reference Network (on rare cardiac diseases, GUARD-Heart) (2018)
	
