# Supplementary material for: European Heart Rhythm Association (EHRA)/Heart Rhythm Society (HRS)/Asia Pacific Heart Rhythm Society (APHRS)/Latin American Heart Rhythm Society (LAHRS) expert consensus on risk assessment in cardiac arrhythmias: use the right tool for the right outcome, in the right population
Source: Europace. 2020 Jun 15;22(8):1147–8. doi: 10.1093/europace/euaa065 (PMC7400488; doi:10.1093/europace/euaa065)
Supplement: euaa065_Supplementary_Data [file euaa065_supplementary_data.zip › DOI_Reviewed EHRA Consensus Doc on risk Assessment in Cardiac Arr Reviewers 2019DoiSummary.docx]

	Buxton Alfred 1- Financial Declaration  A - DIRECT PERSONAL PAYMENT AS IT APPLIES TO YOU AND YOUR SPOUSE/PARTNER OR ANY OTHER MEMBER OF YOUR HOUSEHOLD, OR ANY ENTITY CONTROLLED DIRECTLY OR INDIRECTLY BY ANY OF ABOVE PERSONS: SPEAKER FEES, HONORARIA, CONSULTANCY, ADVISORY BOARD FEES, INVESTIGATOR, COMMITTEE MEMBER, ETC. FROM HEALTHCARE INDUSTRY.                 - Boston Scientific : Implantable Defibrillators (2018)  C - RECEIPT OF ROYALTIES FOR INTELLECTUAL PROPERTY.                 - Wiley Blackwell : Cardiac Arrhythmia (2018) Calvimontes Gonzalo   Nothing to be declared (2018) Chao Tze-Fan   Nothing to be declared (2018) Eckardt Lars 1- Financial Declaration  A - DIRECT PERSONAL PAYMENT AS IT APPLIES TO YOU AND YOUR SPOUSE/PARTNER OR ANY OTHER MEMBER OF YOUR HOUSEHOLD, OR ANY ENTITY CONTROLLED DIRECTLY OR INDIRECTLY BY ANY OF ABOVE PERSONS: SPEAKER FEES, HONORARIA, CONSULTANCY, ADVISORY BOARD FEES, INVESTIGATOR, COMMITTEE MEMBER, ETC. FROM HEALTHCARE INDUSTRY.                 - Abbott : SPEAKER FEES, HONORARIA, CONSULTANCY, ADVISORY BOARD FEES, INVESTIGATOR, COMMITTEE MEMBER (2018)                  - Boehringer-Ingelheim : SPEAKER FEES, HONORARIA, CONSULTANCY, ADVISORY BOARD FEES, INVESTIGATOR, COMMITTEE MEMBER (2018)                  - Boston Scientific : SPEAKER FEES, HONORARIA, CONSULTANCY, ADVISORY BOARD FEES, INVESTIGATOR, COMMITTEE MEMBER (2018)                  - Daiichi Sankyo : SPEAKER FEES, HONORARIA, CONSULTANCY, ADVISORY BOARD FEES, INVESTIGATOR, COMMITTEE MEMBER (2018)                  - Medtronic : SPEAKER FEES, HONORARIA, CONSULTANCY, ADVISORY BOARD FEES, INVESTIGATOR, COMMITTEE MEMBER (2018)                  - Biotronik : SPEAKER FEES, HONORARIA, CONSULTANCY, ADVISORY BOARD FEES, INVESTIGATOR, COMMITTEE MEMBER (2018)                  - Bayer Healthcare : SPEAKER FEES, HONORARIA, CONSULTANCY, ADVISORY BOARD FEES, INVESTIGATOR, COMMITTEE MEMBER (2018)                  - Bristol Myers Squibb : SPEAKER FEES, HONORARIA, CONSULTANCY, ADVISORY BOARD FEES, INVESTIGATOR, COMMITTEE MEMBER (2018)  D - RESEARCH FUNDING UNDER YOUR DIRECT/PERSONAL RESPONSIBILITY (TO DEPARTMENT OR INSTITUTION) FROM HEALTHCARE INDUSTRY.                 - Deutsche Forschungsgemeinschaft : Research Funding (2018)                  - German Cardiac Society : Research Funding (2018) Estner Heidi 1- Financial Declaration  A - DIRECT PERSONAL PAYMENT AS IT APPLIES TO YOU AND YOUR SPOUSE/PARTNER OR ANY OTHER MEMBER OF YOUR HOUSEHOLD, OR ANY ENTITY CONTROLLED DIRECTLY OR INDIRECTLY BY ANY OF ABOVE PERSONS: SPEAKER FEES, HONORARIA, CONSULTANCY, ADVISORY BOARD FEES, INVESTIGATOR, COMMITTEE MEMBER, ETC. FROM HEALTHCARE INDUSTRY.                 - Boston Scientific : Honoraria fee (2018)                  - German Cardiac Society : Honoraria fee (2018) Gillis Anne 1- Financial Declaration  D - RESEARCH FUNDING UNDER YOUR DIRECT/PERSONAL RESPONSIBILITY (TO DEPARTMENT OR INSTITUTION) FROM HEALTHCARE INDUSTRY.                 - Medtronic : Implantable Devices (2018)  2- Other Positions of Influence  2.4 - Any other interest (financial or otherwise) that should be declared in view of holding an ESC position.                 - American Heart Association, Associate Editor Circulation Arrhythmias and Electrophysiology (2018) Isa Rodrigo   Nothing to be declared (2018) Kautzner Josef 1- Financial Declaration  A - DIRECT PERSONAL PAYMENT AS IT APPLIES TO YOU AND YOUR SPOUSE/PARTNER OR ANY OTHER MEMBER OF YOUR HOUSEHOLD, OR ANY ENTITY CONTROLLED DIRECTLY OR INDIRECTLY BY ANY OF ABOVE PERSONS: SPEAKER FEES, HONORARIA, CONSULTANCY, ADVISORY BOARD FEES, INVESTIGATOR, COMMITTEE MEMBER, ETC. FROM HEALTHCARE INDUSTRY.                 - EPIX : ablation catheter (2018)                  - Biosense Webster : catheters, mapping system (2018)                  - Bayer : drugs (2018)                  - Boehringer-Ingelheim : drugs (2018)                  - Daiichi Sankyo : drugs (2018)                  - Pfizer : drugs (2018)                  - Bayer Healthcare : drugs (2018)                  - MSD : drugs (2018)                  - Boston Scientific : pacemakers, ICDs (2018)                  - Biotronik : pacemakers, ICDs, catheters (2018)                  - Liva nova (Sorin) : pacemakers, ICDs, catheters (2018)                  - Medtronic : pacemakers, ICDs, catheters, mapping system (2018)                  - St Jude Medical (Abbott) : pacemakers, ICDs, catheters, mapping system (2018) Maury Philippe 1- Financial Declaration  B - PAYMENT TO YOUR DEPARTMENT OR INSTITUTION OR ANY OTHER BODY LIKE AN ASSOCIATION OR SIMILAR FOR YOUR PERSONAL SERVICES: SPEAKER FEES, HONORARIA, CONSULTANCY, ADVISORY BOARD FEES, INVESTIGATOR, COMMITTEE MEMBER, ETC. FROM HEALTHCARE INDUSTRY.                 - Boston Scientific : EP (2018) Moss Joshua 1- Financial Declaration  A - DIRECT PERSONAL PAYMENT AS IT APPLIES TO YOU AND YOUR SPOUSE/PARTNER OR ANY OTHER MEMBER OF YOUR HOUSEHOLD, OR ANY ENTITY CONTROLLED DIRECTLY OR INDIRECTLY BY ANY OF ABOVE PERSONS: SPEAKER FEES, HONORARIA, CONSULTANCY, ADVISORY BOARD FEES, INVESTIGATOR, COMMITTEE MEMBER, ETC. FROM HEALTHCARE INDUSTRY.                 - Boston Scientific : Electrophysiology (2018)                  - Biosense Webster : Electrophysiology (2018)                  - Vytronus, Inc : Electrophysiology (2018) Nam Gi-Byoung   Nothing to be declared (2018) Olshansky Brian 1- Financial Declaration  A - DIRECT PERSONAL PAYMENT AS IT APPLIES TO YOU AND YOUR SPOUSE/PARTNER OR ANY OTHER MEMBER OF YOUR HOUSEHOLD, OR ANY ENTITY CONTROLLED DIRECTLY OR INDIRECTLY BY ANY OF ABOVE PERSONS: SPEAKER FEES, HONORARIA, CONSULTANCY, ADVISORY BOARD FEES, INVESTIGATOR, COMMITTEE MEMBER, ETC. FROM HEALTHCARE INDUSTRY.                 - Lundbeck : Droxidopa (2018)                  - Respironics : phrenic nerve stimulator (2018)                  - Boehringer-Ingelheim : Pradaxa (2018) Pava Luis   Nothing to be declared (2018) Pimentel Mauricio 1- Financial Declaration  A - DIRECT PERSONAL PAYMENT AS IT APPLIES TO YOU AND YOUR SPOUSE/PARTNER OR ANY OTHER MEMBER OF YOUR HOUSEHOLD, OR ANY ENTITY CONTROLLED DIRECTLY OR INDIRECTLY BY ANY OF ABOVE PERSONS: SPEAKER FEES, HONORARIA, CONSULTANCY, ADVISORY BOARD FEES, INVESTIGATOR, COMMITTEE MEMBER, ETC. FROM HEALTHCARE INDUSTRY.                 - Daiichi Sankyo : Edoxaban (2018)                  - Bayer : Rivaroxaban (2018) Prabhu Mukundaprabhu   Nothing to be declared (2018) Sommer Philipp 1- Financial Declaration  A - DIRECT PERSONAL PAYMENT AS IT APPLIES TO YOU AND YOUR SPOUSE/PARTNER OR ANY OTHER MEMBER OF YOUR HOUSEHOLD, OR ANY ENTITY CONTROLLED DIRECTLY OR INDIRECTLY BY ANY OF ABOVE PERSONS: SPEAKER FEES, HONORARIA, CONSULTANCY, ADVISORY BOARD FEES, INVESTIGATOR, COMMITTEE MEMBER, ETC. FROM HEALTHCARE INDUSTRY.                 - Abbott : Ablation (2018)                  - Biosense Webster : Ablation (2018)                  - Boehringer-Ingelheim : NOAC (2018)                  - Daiichi Sankyo : NOAC (2018)                  - Bayer Healthcare : NOAC (2018)                  - Bristol Myers Squibb : NOAC (2018)  B - PAYMENT TO YOUR DEPARTMENT OR INSTITUTION OR ANY OTHER BODY LIKE AN ASSOCIATION OR SIMILAR FOR YOUR PERSONAL SERVICES: SPEAKER FEES, HONORARIA, CONSULTANCY, ADVISORY BOARD FEES, INVESTIGATOR, COMMITTEE MEMBER, ETC. FROM HEALTHCARE INDUSTRY.                 - Abbott : Innovation  (2018)                  - Biosense Webster : Innovation (2018) Swampillai Janice   Nothing to be declared (2018) Tzou Wendy 1- Financial Declaration  A - DIRECT PERSONAL PAYMENT AS IT APPLIES TO YOU AND YOUR SPOUSE/PARTNER OR ANY OTHER MEMBER OF YOUR HOUSEHOLD, OR ANY ENTITY CONTROLLED DIRECTLY OR INDIRECTLY BY ANY OF ABOVE PERSONS: SPEAKER FEES, HONORARIA, CONSULTANCY, ADVISORY BOARD FEES, INVESTIGATOR, COMMITTEE MEMBER, ETC. FROM HEALTHCARE INDUSTRY.                 - Biotronik : CRM (2018)                  - Abbott : CRM and Electrophysiology (2018)                  - Boston Scientific : CRM and Electrophysiology (2018)                  - Medtronic : CRM and Electrophysiology (2018)                  - Biosense Webster : Electrophysiology mapping and ablation (2018)                  - BioSig : Electrophysiology Mapping and Ablation (2018)  B - PAYMENT TO YOUR DEPARTMENT OR INSTITUTION OR ANY OTHER BODY LIKE AN ASSOCIATION OR SIMILAR FOR YOUR PERSONAL SERVICES: SPEAKER FEES, HONORARIA, CONSULTANCY, ADVISORY BOARD FEES, INVESTIGATOR, COMMITTEE MEMBER, ETC. FROM HEALTHCARE INDUSTRY.                 - Biosense Webster : Catheter Ablation (2018)                  - Abbott : Catheter Ablation and CRM (2018)  D - RESEARCH FUNDING UNDER YOUR DIRECT/PERSONAL RESPONSIBILITY (TO DEPARTMENT OR INSTITUTION) FROM HEALTHCARE INDUSTRY.                 - Abbott : Catheter Ablation (2018)                  - Boston Scientific : CRM (2018) Vidal Alejandro   Nothing to be declared (2018)	
Buxton Alfred	1- Financial Declaration
	A - DIRECT PERSONAL PAYMENT AS IT APPLIES TO YOU AND YOUR SPOUSE/PARTNER OR ANY OTHER MEMBER OF YOUR HOUSEHOLD, OR ANY ENTITY CONTROLLED DIRECTLY OR INDIRECTLY BY ANY OF ABOVE PERSONS: SPEAKER FEES, HONORARIA, CONSULTANCY, ADVISORY BOARD FEES, INVESTIGATOR, COMMITTEE MEMBER, ETC. FROM HEALTHCARE INDUSTRY.                 - Boston Scientific : Implantable Defibrillators (2018)
	C - RECEIPT OF ROYALTIES FOR INTELLECTUAL PROPERTY.                 - Wiley Blackwell : Cardiac Arrhythmia (2018)
Calvimontes Gonzalo	
	Nothing to be declared (2018)
Chao Tze-Fan	
	Nothing to be declared (2018)
Eckardt Lars	1- Financial Declaration
	A - DIRECT PERSONAL PAYMENT AS IT APPLIES TO YOU AND YOUR SPOUSE/PARTNER OR ANY OTHER MEMBER OF YOUR HOUSEHOLD, OR ANY ENTITY CONTROLLED DIRECTLY OR INDIRECTLY BY ANY OF ABOVE PERSONS: SPEAKER FEES, HONORARIA, CONSULTANCY, ADVISORY BOARD FEES, INVESTIGATOR, COMMITTEE MEMBER, ETC. FROM HEALTHCARE INDUSTRY.                 - Abbott : SPEAKER FEES, HONORARIA, CONSULTANCY, ADVISORY BOARD FEES, INVESTIGATOR, COMMITTEE MEMBER (2018)
	- Boehringer-Ingelheim : SPEAKER FEES, HONORARIA, CONSULTANCY, ADVISORY BOARD FEES, INVESTIGATOR, COMMITTEE MEMBER (2018)
	- Boston Scientific : SPEAKER FEES, HONORARIA, CONSULTANCY, ADVISORY BOARD FEES, INVESTIGATOR, COMMITTEE MEMBER (2018)
	- Daiichi Sankyo : SPEAKER FEES, HONORARIA, CONSULTANCY, ADVISORY BOARD FEES, INVESTIGATOR, COMMITTEE MEMBER (2018)
	- Medtronic : SPEAKER FEES, HONORARIA, CONSULTANCY, ADVISORY BOARD FEES, INVESTIGATOR, COMMITTEE MEMBER (2018)
	- Biotronik : SPEAKER FEES, HONORARIA, CONSULTANCY, ADVISORY BOARD FEES, INVESTIGATOR, COMMITTEE MEMBER (2018)
	- Bayer Healthcare : SPEAKER FEES, HONORARIA, CONSULTANCY, ADVISORY BOARD FEES, INVESTIGATOR, COMMITTEE MEMBER (2018)
	- Bristol Myers Squibb : SPEAKER FEES, HONORARIA, CONSULTANCY, ADVISORY BOARD FEES, INVESTIGATOR, COMMITTEE MEMBER (2018)
	D - RESEARCH FUNDING UNDER YOUR DIRECT/PERSONAL RESPONSIBILITY (TO DEPARTMENT OR INSTITUTION) FROM HEALTHCARE INDUSTRY.                 - Deutsche Forschungsgemeinschaft : Research Funding (2018)
	- German Cardiac Society : Research Funding (2018)
Estner Heidi	1- Financial Declaration
	A - DIRECT PERSONAL PAYMENT AS IT APPLIES TO YOU AND YOUR SPOUSE/PARTNER OR ANY OTHER MEMBER OF YOUR HOUSEHOLD, OR ANY ENTITY CONTROLLED DIRECTLY OR INDIRECTLY BY ANY OF ABOVE PERSONS: SPEAKER FEES, HONORARIA, CONSULTANCY, ADVISORY BOARD FEES, INVESTIGATOR, COMMITTEE MEMBER, ETC. FROM HEALTHCARE INDUSTRY.                 - Boston Scientific : Honoraria fee (2018)
	- German Cardiac Society : Honoraria fee (2018)
Gillis Anne	1- Financial Declaration
	D - RESEARCH FUNDING UNDER YOUR DIRECT/PERSONAL RESPONSIBILITY (TO DEPARTMENT OR INSTITUTION) FROM HEALTHCARE INDUSTRY.                 - Medtronic : Implantable Devices (2018)
	2- Other Positions of Influence
	2.4 - Any other interest (financial or otherwise) that should be declared in view of holding an ESC position.                 - American Heart Association, Associate Editor Circulation Arrhythmias and Electrophysiology (2018)
Isa Rodrigo	
	Nothing to be declared (2018)
Kautzner Josef	1- Financial Declaration
	A - DIRECT PERSONAL PAYMENT AS IT APPLIES TO YOU AND YOUR SPOUSE/PARTNER OR ANY OTHER MEMBER OF YOUR HOUSEHOLD, OR ANY ENTITY CONTROLLED DIRECTLY OR INDIRECTLY BY ANY OF ABOVE PERSONS: SPEAKER FEES, HONORARIA, CONSULTANCY, ADVISORY BOARD FEES, INVESTIGATOR, COMMITTEE MEMBER, ETC. FROM HEALTHCARE INDUSTRY.                 - EPIX : ablation catheter (2018)
	- Biosense Webster : catheters, mapping system (2018)
	- Bayer : drugs (2018)
	- Boehringer-Ingelheim : drugs (2018)
	- Daiichi Sankyo : drugs (2018)
	- Pfizer : drugs (2018)
	- Bayer Healthcare : drugs (2018)
	- MSD : drugs (2018)
	- Boston Scientific : pacemakers, ICDs (2018)
	- Biotronik : pacemakers, ICDs, catheters (2018)
	- Liva nova (Sorin) : pacemakers, ICDs, catheters (2018)
	- Medtronic : pacemakers, ICDs, catheters, mapping system (2018)
	- St Jude Medical (Abbott) : pacemakers, ICDs, catheters, mapping system (2018)
Maury Philippe	1- Financial Declaration
	B - PAYMENT TO YOUR DEPARTMENT OR INSTITUTION OR ANY OTHER BODY LIKE AN ASSOCIATION OR SIMILAR FOR YOUR PERSONAL SERVICES: SPEAKER FEES, HONORARIA, CONSULTANCY, ADVISORY BOARD FEES, INVESTIGATOR, COMMITTEE MEMBER, ETC. FROM HEALTHCARE INDUSTRY.                 - Boston Scientific : EP (2018)
Moss Joshua	1- Financial Declaration
	A - DIRECT PERSONAL PAYMENT AS IT APPLIES TO YOU AND YOUR SPOUSE/PARTNER OR ANY OTHER MEMBER OF YOUR HOUSEHOLD, OR ANY ENTITY CONTROLLED DIRECTLY OR INDIRECTLY BY ANY OF ABOVE PERSONS: SPEAKER FEES, HONORARIA, CONSULTANCY, ADVISORY BOARD FEES, INVESTIGATOR, COMMITTEE MEMBER, ETC. FROM HEALTHCARE INDUSTRY.                 - Boston Scientific : Electrophysiology (2018)
	- Biosense Webster : Electrophysiology (2018)
	- Vytronus, Inc : Electrophysiology (2018)
Nam Gi-Byoung	
	Nothing to be declared (2018)
Olshansky Brian	1- Financial Declaration
	A - DIRECT PERSONAL PAYMENT AS IT APPLIES TO YOU AND YOUR SPOUSE/PARTNER OR ANY OTHER MEMBER OF YOUR HOUSEHOLD, OR ANY ENTITY CONTROLLED DIRECTLY OR INDIRECTLY BY ANY OF ABOVE PERSONS: SPEAKER FEES, HONORARIA, CONSULTANCY, ADVISORY BOARD FEES, INVESTIGATOR, COMMITTEE MEMBER, ETC. FROM HEALTHCARE INDUSTRY.                 - Lundbeck : Droxidopa (2018)
	- Respironics : phrenic nerve stimulator (2018)
	- Boehringer-Ingelheim : Pradaxa (2018)
Pava Luis	
	Nothing to be declared (2018)
Pimentel Mauricio	1- Financial Declaration
	A - DIRECT PERSONAL PAYMENT AS IT APPLIES TO YOU AND YOUR SPOUSE/PARTNER OR ANY OTHER MEMBER OF YOUR HOUSEHOLD, OR ANY ENTITY CONTROLLED DIRECTLY OR INDIRECTLY BY ANY OF ABOVE PERSONS: SPEAKER FEES, HONORARIA, CONSULTANCY, ADVISORY BOARD FEES, INVESTIGATOR, COMMITTEE MEMBER, ETC. FROM HEALTHCARE INDUSTRY.                 - Daiichi Sankyo : Edoxaban (2018)
	- Bayer : Rivaroxaban (2018)
Prabhu Mukundaprabhu	
	Nothing to be declared (2018)
Sommer Philipp	1- Financial Declaration
	A - DIRECT PERSONAL PAYMENT AS IT APPLIES TO YOU AND YOUR SPOUSE/PARTNER OR ANY OTHER MEMBER OF YOUR HOUSEHOLD, OR ANY ENTITY CONTROLLED DIRECTLY OR INDIRECTLY BY ANY OF ABOVE PERSONS: SPEAKER FEES, HONORARIA, CONSULTANCY, ADVISORY BOARD FEES, INVESTIGATOR, COMMITTEE MEMBER, ETC. FROM HEALTHCARE INDUSTRY.                 - Abbott : Ablation (2018)
	- Biosense Webster : Ablation (2018)
	- Boehringer-Ingelheim : NOAC (2018)
	- Daiichi Sankyo : NOAC (2018)
	- Bayer Healthcare : NOAC (2018)
	- Bristol Myers Squibb : NOAC (2018)
	B - PAYMENT TO YOUR DEPARTMENT OR INSTITUTION OR ANY OTHER BODY LIKE AN ASSOCIATION OR SIMILAR FOR YOUR PERSONAL SERVICES: SPEAKER FEES, HONORARIA, CONSULTANCY, ADVISORY BOARD FEES, INVESTIGATOR, COMMITTEE MEMBER, ETC. FROM HEALTHCARE INDUSTRY.                 - Abbott : Innovation  (2018)
	- Biosense Webster : Innovation (2018)
Swampillai Janice	
	Nothing to be declared (2018)
Tzou Wendy	1- Financial Declaration
	A - DIRECT PERSONAL PAYMENT AS IT APPLIES TO YOU AND YOUR SPOUSE/PARTNER OR ANY OTHER MEMBER OF YOUR HOUSEHOLD, OR ANY ENTITY CONTROLLED DIRECTLY OR INDIRECTLY BY ANY OF ABOVE PERSONS: SPEAKER FEES, HONORARIA, CONSULTANCY, ADVISORY BOARD FEES, INVESTIGATOR, COMMITTEE MEMBER, ETC. FROM HEALTHCARE INDUSTRY.                 - Biotronik : CRM (2018)
	- Abbott : CRM and Electrophysiology (2018)
	- Boston Scientific : CRM and Electrophysiology (2018)
	- Medtronic : CRM and Electrophysiology (2018)
	- Biosense Webster : Electrophysiology mapping and ablation (2018)
	- BioSig : Electrophysiology Mapping and Ablation (2018)
	B - PAYMENT TO YOUR DEPARTMENT OR INSTITUTION OR ANY OTHER BODY LIKE AN ASSOCIATION OR SIMILAR FOR YOUR PERSONAL SERVICES: SPEAKER FEES, HONORARIA, CONSULTANCY, ADVISORY BOARD FEES, INVESTIGATOR, COMMITTEE MEMBER, ETC. FROM HEALTHCARE INDUSTRY.                 - Biosense Webster : Catheter Ablation (2018)
	- Abbott : Catheter Ablation and CRM (2018)
	D - RESEARCH FUNDING UNDER YOUR DIRECT/PERSONAL RESPONSIBILITY (TO DEPARTMENT OR INSTITUTION) FROM HEALTHCARE INDUSTRY.                 - Abbott : Catheter Ablation (2018)
	- Boston Scientific : CRM (2018)
Vidal Alejandro	
	Nothing to be declared (2018)
	
